# Supplementary material for: Thermodynamics of consciousness: Non-equilibrium brain dynamics track conscious states
Source: Cell Rep. 2026 Jul 31;45(8):117782. doi: 10.1016/j.celrep.2026.117782 (PMC13506674; doi:10.1016/j.celrep.2026.117782)
Supplement: Supplementary file 1 [file mmc1.pdf]

Cell Reports, Volume *45*

Supplemental information

Thermodynamics of consciousness: Non-equilibrium brain dynamics track conscious states

Tomas Berjaga-Buisan, Juan Manuel Monti, Martina Cortada, Michele A. Colombo, Sebastian M. Geli, Gianluca Gaglioti, Simone Sarasso, Morten L. Kringelbach, Maurizio Corbetta, Maria V. Sanchez-Vives, Marcello Massimini, Yonatan Sanz Perl, and Gustavo Deco

SUPPLEMENTARY INFORMATION

Supplementary Note 1. Illustrative spontaneous traces of electrophysiological recordings

To qualitatively illustrate the temporal structure of brain activity across levels of consciousness, we present representative spontaneous electrophysiological recordings from mice and humans. In mice, local field potential (LFP) traces exhibit a clear progression as anesthesia deepens, with activity increasingly dominated by large, stereotyped Up–Down state transitions. As isoflurane concentration increases, Up states become shorter and occur less frequently, consistent with strengthened cortical bistability and a greater predominance of Down states (Figure S1A).

Human electroencephalography (EEG) recordings reveal a comparable state dependence. During anesthesia, the signal is dominated by large-amplitude slow oscillations, indicative of widespread cortical synchrony and reduced responsiveness. In contrast, wakefulness is characterized by lower-amplitude, desynchronized activity with faster components, consistent with active and differentiated neural processing (Figure S1B). Together, these examples provide qualitative insight into how spontaneous brain activity is reorganized across species as the brain transitions between conscious and anesthetized states.

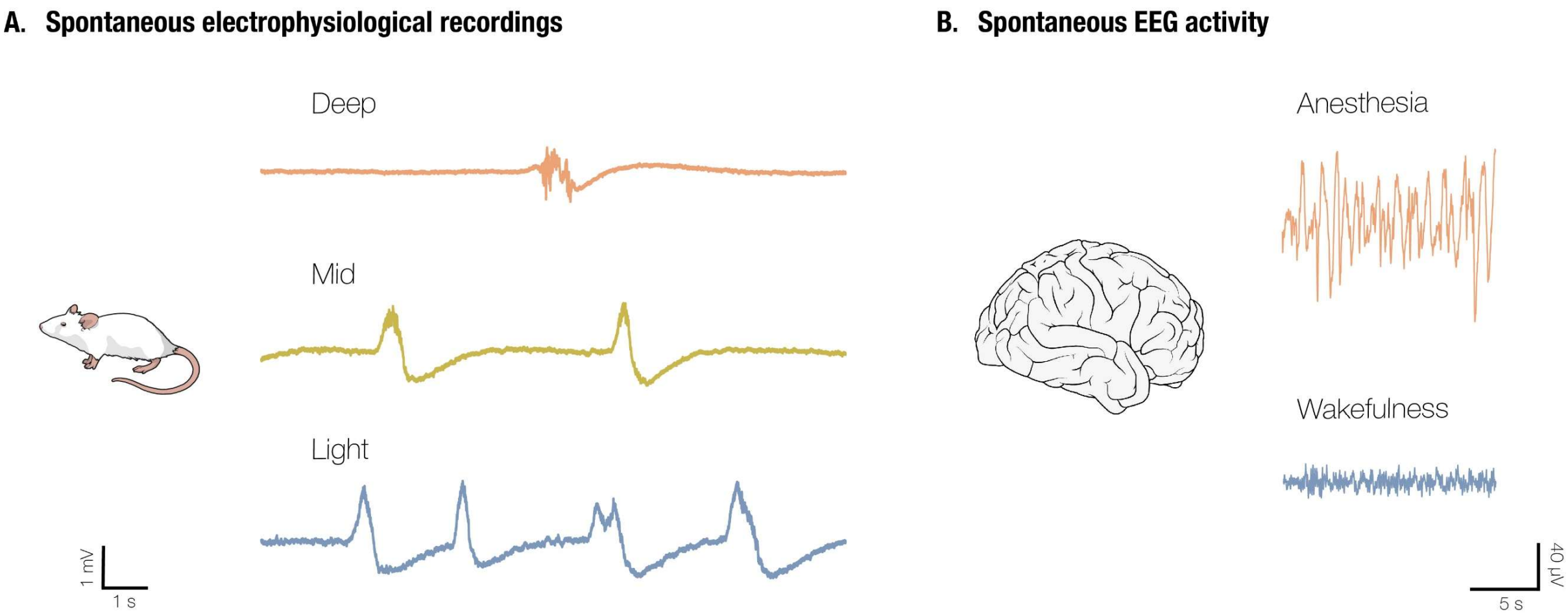

Figure S1. **Illustrative spontaneous electrophysiological recordings. (A)** Spontaneous LFP traces from the same mouse under three different levels of graded anesthesia, defined by increasing isoflurane concentrations. As observed, neural activity under anesthesia becomes dominated by stereotyped Up–Down state transitions characteristic of the anesthetized cortex. **(B)** Representative human EEG patterns during anesthesia, characterized by dominant slow oscillations, and during wakefulness, marked by lower-amplitude, desynchronized activity with prominent alpha rhythms.

Supplementary Note 2. FDT violations correlate with the frequency of the slow oscillation in animal models

Slow oscillations (SO) are a hallmark of brain states associated with reduced complexity. They are prominently observed during non-rapid eye movement sleep, in patients with unresponsive wakefulness syndrome (UWS), even during eyes-open periods, and in pathological conditions such as stroke [S1, S2, S3, S4]. These rhythms reflect cortical bistability, arising from alternating phases of neuronal activity and silence [S5]. Experimental and computational studies across humans, rodents, and cortical slice preparations converge in identifying cortical bistability and OFF-periods as major disruptors of large-scale network integration and complexity [S6, S7, S8, S9, S10]. In this framework, such bistable dynamics are thought to constrain the emergence of rich, differentiated cortical responses, thereby reducing perturbational complexity and FDT violations.

In the mouse anesthesia dataset, the dominant frequency of SO varied systematically across anesthesia levels and showed a strong monotonic relationship with anesthesia depth (Spearman's $\rho = 0.87$, $p < 0.001$), indicating that slower oscillatory dynamics were associated with deeper anesthesia. PCI was significantly correlated with SO frequency (Spearman's $\rho = 0.69$, $p < 0.001$; Pearson's $r = 0.76$, $p < 0.001$; Figure S2A), consistent with the idea that slower and more bistable cortical dynamics are associated with reduced perturbational complexity. Likewise, FDT violations exhibited an even stronger association with SO frequency (Spearman's $\rho = 0.80$, $p < 0.001$; Pearson's $r = 0.83$, $p < 0.001$; Figure S2B), indicating that the dominant frequency of spontaneous slow oscillations is closely linked to the degree of effective departure from equilibrium estimated from the whole-brain model.

We further evaluated robustness using leave-one-subject-out cross-validation. In held-out subjects, the association remained significant for PCI (Spearman's $\rho = 0.6252$, $p = 0.0011$; Pearson's $r = 0.6567$, $p < 0.001$) and for FDT violations (Spearman's $\rho = 0.8139$, $p < 0.001$; Pearson's $r = 0.8169$, $p < 0.001$).

Together, these findings show that the dominant frequency of SO not only tracks graded anesthesia depth but is also tightly linked to both perturbational complexity and non-equilibrium brain dynamics. This result is consistent with the idea that slow oscillatory bistable regimes impose strong constraints on large-scale integration, thereby reducing both the complexity of evoked cortical responses and the degree to which spontaneous activity departs from equilibrium.

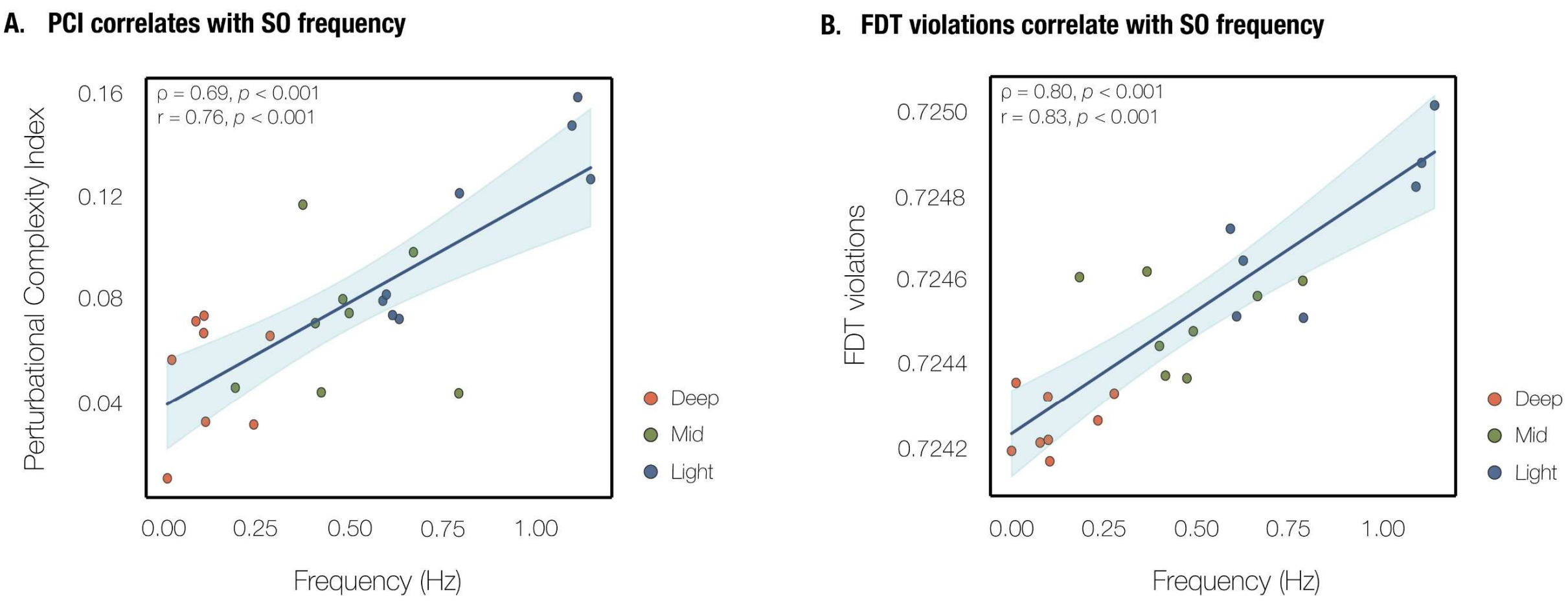

Figure S2. **Intrinsic slow oscillation (SO) frequency correlates with PCI and FDT violations in animal models. (A)** PCI correlates with the intrinsic frequency of the SO (Spearman's ρ = 0.69, $p < 0.001$; Pearson's r = 0.76, $p < 0.001$). It generalizes under leave-one-subject-out cross-validation (Spearman's ρ = 0.6252, $p = 0.0011$; Pearson's r = 0.6567, $p < 0.001$). **(B)** FDT violations also show a strong correlation with the intrinsic frequency of the SO, capturing its spatiotemporal structure with high sensitivity (Spearman's ρ = 0.80, $p < 0.001$; Pearson's r = 0.83, $p < 0.001$). It generalizes under leave-one-subject-out cross-validation (Spearman's ρ = 0.8139, $p < 0.001$; Pearson's r = 0.8169, $p < 0.001$).

Supplementary Note 3. Post-anoxic patients define a boundary condition of the framework

To better define the scope of the main findings, we analyzed the subgroup of patients with post-anoxic etiology separately from the primary non-anoxic disorders of consciousness (DoC) cohort. This subgroup was not included in the main analysis because severe post-anoxic injury is associated with diffuse cortical damage, marked suppression of electrophysiological activity, and low-voltage EEG, conditions under which both perturbational and spontaneous measures may become constrained by cortical integrity rather than providing a meaningful graded index of consciousness capacity [S11, S12].

The post-anoxic subgroup included 9 patients. All patients underwent the same acquisition, preprocessing, PCI computation, and FDT violations estimation procedures as described for the main DoC cohort. In this subgroup, PCI showed a marked floor effect. In particular, all UWS patients had PCI values at floor, consistent with absent or severely reduced perturbational responses. Accordingly, PCI still separated diagnostic categories at the group level (Cliff's $\delta = 1.0$, $p = 0.0108$; AROC = 1.0, Figure S3A). Under these conditions, however, PCI does not provide a meaningful graded variable, and therefore correlations cannot be interpreted in the same way as in the main non-anoxic DoC cohort.

Spontaneous EEG activity in the post-anoxic subgroup was frequently low-voltage, reducing the effective signal-to-noise ratio available for model-based estimation of directional and non-equilibrium structure. Nevertheless, FDT violations could still be estimated and retained variability across patients. At the group level, separation of FDT violations between diagnostic categories was modest and did not reach statistical significance (Cliff's $\delta = 0.700$, $p = 0.11$; Figure S3B). Given the modest separation, the limited sample size, and the likely heterogeneity in cortical injury and residual electrophysiological integrity within this subgroup, these results should be reported descriptively and interpreted cautiously (AROC = 0.85).

Because PCI was floor-compressed in UWS, we do not interpret this subgroup as providing an equivalent graded test of the relationship between PCI and FDT violations. Rather, these findings identify severe post-anoxic injury as a boundary condition of the framework, in which diffuse cortical damage constrains the dynamic range of both perturbational and spontaneous measures.

Taken together, these findings suggest that severe post-anoxic injury defines a clinically important boundary condition for the present framework. In this regime, diffuse cortical damage appears to compress the available electrophysiological repertoire and reduce the discriminatory range of both perturbational and spontaneous measures. Accordingly, the main conclusions of the study should be interpreted as applying to conditions in which spontaneous and perturbational brain dynamics remain sufficiently preserved to support meaningful estimation of PCI and FDT violations.

A. PCI in post-anoxic patients

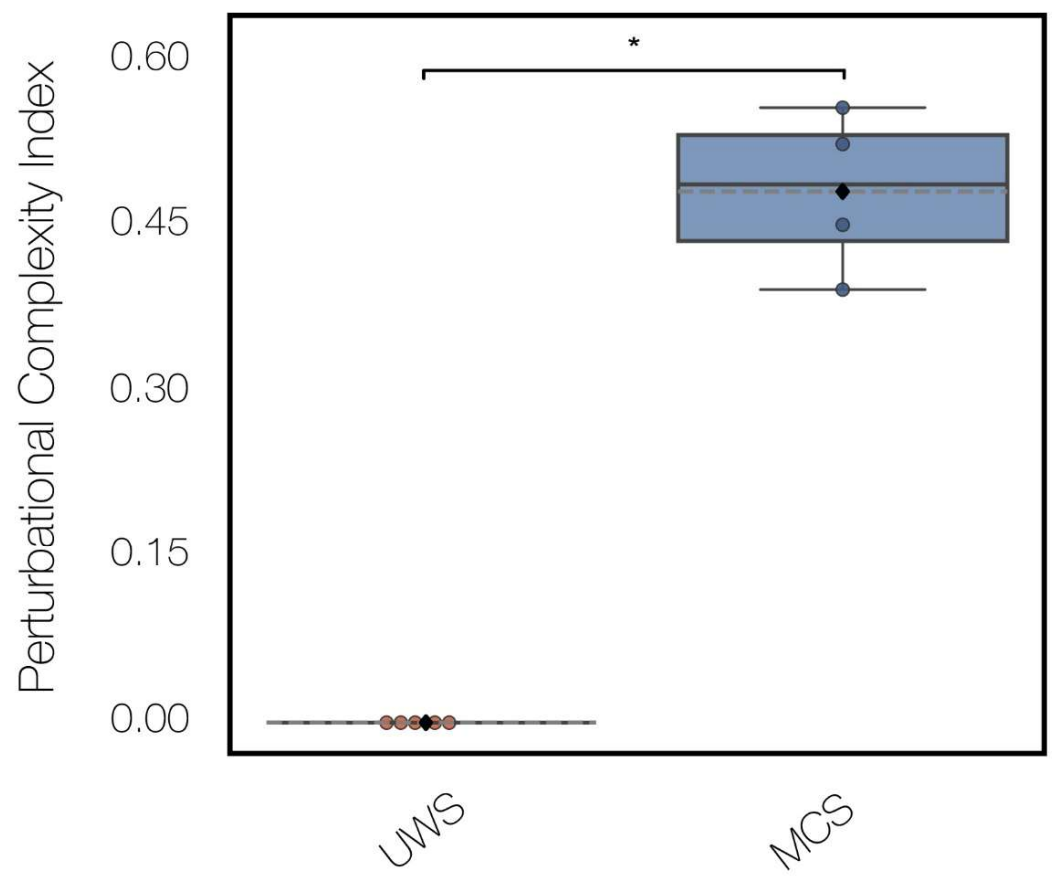

B. FDT violations in post-anoxic patients

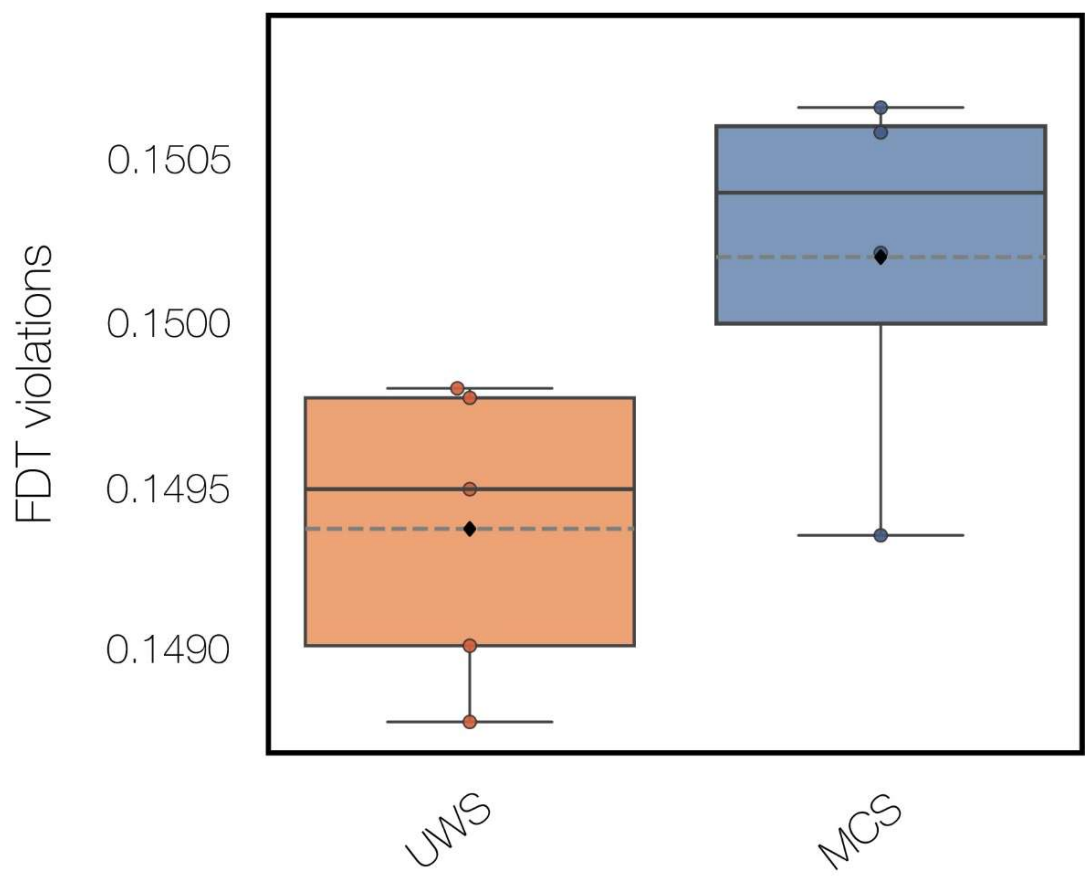

Figure S3. **PCI and FDT violations in the post-anoxic subgroup. (A)** PCI values in post-anoxic patients separated UWS and MCS at the group level (Cliff's $\delta = 1.0$, p = 0.0108; AROC = 1.0), but this separation was driven by a marked floor effect in UWS, with all UWS patients showing PCI values at floor. **(B)** FDT violations retained variability across post-anoxic patients, but separation between diagnostic categories was modest and did not reach statistical significance (Cliff's $\delta = 0.700$, p = 0.11; AROC = 0.85). These results identify severe post-anoxic injury as a boundary condition of the framework, in which diffuse cortical damage constrains the dynamic range of perturbational and spontaneous measures. * $p < 0.05$

Supplementary Note 4. FDT violations correlate with other non-equilibrium measures

Entropy production is one of the canonical physical measures of deviation from equilibrium. In the model used in this work, the entropy production rate (EPR) can be calculated analytically [S13]. We consider a linear Langevin system described by:

$$\frac{dx}{dt} = -Bx(t) + \eta(t) \qquad (S1)$$

where $\eta(t)$ is homogeneous white Gaussian noise, satisfying $< \eta(t)\eta(t') > = 2\,\sigma^2\delta(t-t')$. The EPR is then given by:

$$\phi = -\frac{1}{\sigma^2} tr(B\Theta) \qquad (S2)$$

Here, $\Theta = BK - \sigma^2 I$, where K is the stationary covariance matrix. We then computed the EPR for each fitted brain model according to Equation S2. A key characteristic of non-equilibrium systems is their temporal irreversibility. Following previous work [S14, S15], we quantified this irreversibility by measuring the asymmetry of time-lagged covariances. Specifically, we computed the difference between the lagged covariance matrix and its transpose:

$$A_c = \left\| KS^{model}(\tau) - (KS^{model}(\tau))^T \right\|_{\tau=dt} \qquad (S3)$$

where $\|\cdot\|$ denotes the Frobenius norm, i.e., the square root of the sum of the squared matrix elements, and A_C quantifies the asymmetry of the lagged covariances evaluated at $\tau = dt$.

Lastly, we examined the asymmetry of the fitted coupling matrix, which has been highlighted as a key mechanism for breaking detailed balance in interacting systems [S15], inducing FDT violations [S16]. Following previous work [S17], we characterized coupling asymmetry using irreciprocity. We define the reciprocated portion of the coupling between two nodes:

$$\overleftrightarrow{B}ij = min(Bij, Bji) = \overleftrightarrow{B}ji \qquad (S4)$$

The global reciprocity of the coupling is then defined as the ratio between the total reciprocated weight and the total connection weight:

$$r(B) = \frac{\sum_{i\neq j} \overleftrightarrow{B}ij}{\sum_{i\neq j} Bij} \qquad (S5)$$

Finally, we define the irreciprocity of the coupling as $A_B = 1 - r$, which lies in the range [0,1]. A value of $A_B = 0$ corresponds to a perfectly reciprocated coupling, while $A_B = 1$ corresponds to a completely unreciprocated coupling.

We reanalyzed the three datasets incorporating the non-equilibrium measures described above.

Figure S4 shows the results for the mouse dataset. EPR (Figure S4A), temporal irreversibility (Figure S4B), and coupling asymmetry (Figure S4C) all decreased with increasing anesthesia depth. These metrics showed clear separation between light and deep anesthesia, whereas the intermediate condition was less consistently distinguished from the other two. Nevertheless, the Spearman correlation coefficients indicate that all three metrics tracked anesthesia depth across conditions (EPR: Spearman's $\rho = 0.67$, $p < 0.001$; temporal irreversibility: Spearman's $\rho = 0.67$, $p < 0.001$; coupling asymmetry: Spearman's $\rho = 0.70$, $p < 0.001$). Furthermore, FDT violations were positively associated with EPR (Spearman's $\rho = 0.72$, $p < 0.001$; Pearson's $r = 0.66$, $p < 0.001$), temporal irreversibility (Spearman's $\rho = 0.72$, $p < 0.001$; Pearson's $r = 0.78$, $p < 0.001$), and coupling asymmetry (Spearman's $\rho = 0.77$, $p < 0.001$; Pearson's $r = 0.70$, $p < 0.001$).

Figure S5 presents the results for the three metrics in the human EEG recordings obtained during wakefulness and anesthesia. Across participants, EPR (Figure S5A), temporal irreversibility (Figure S5B), and coupling asymmetry (Figure S5C) were reduced under xenon and propofol relative to wakefulness. No significant differences were observed between ketamine and wakefulness, consistent with the main results for PCI and FDT violations. As observed for FDT violations, EPR, temporal irreversibility, and coupling asymmetry were higher in participants who reported conscious experience (wakefulness and ketamine; B+) than in those who did not (xenon and propofol; B-). FDT violations were positively associated with EPR (Spearman's $\rho = 0.67$, $p < 0.001$; Pearson's $r = 0.60$, $p < 0.001$), temporal irreversibility (Spearman's $\rho = 0.67$, $p < 0.001$; Pearson's $r = 0.66$, $p < 0.001$), and coupling asymmetry (Spearman's $\rho = 0.64$, $p < 0.001$; Pearson's $r = 0.63$, $p < 0.001$) (Figures S5D–F).

Lastly, we analyzed the DoC dataset (Figure S6). All non-equilibrium measures showed lower values in patients without signs of consciousness (UWS) than in patients with conscious signs (MCS−/MCS+), mirroring the pattern observed for FDT violations and PCI. These results should be interpreted cautiously, as the modest sample size and clinical heterogeneity of the DoC cohort may have limited statistical power despite directional consistency with the main findings. At the pooled level, FDT violations showed a significant positive correlation with temporal irreversibility (Spearman's $\rho = 0.51$, $p = 0.038$; Pearson's $r = 0.50$, $p = 0.0412$). EPR and coupling asymmetry showed positive trend-level associations in the same direction (EPR: Spearman's $\rho = 0.48$, $p = 0.051$; Pearson's $r = 0.44$, $p = 0.0762$; coupling asymmetry: Spearman's $\rho = 0.47$, $p = 0.0583$; Pearson's $r = 0.46$, $p = 0.0643$).

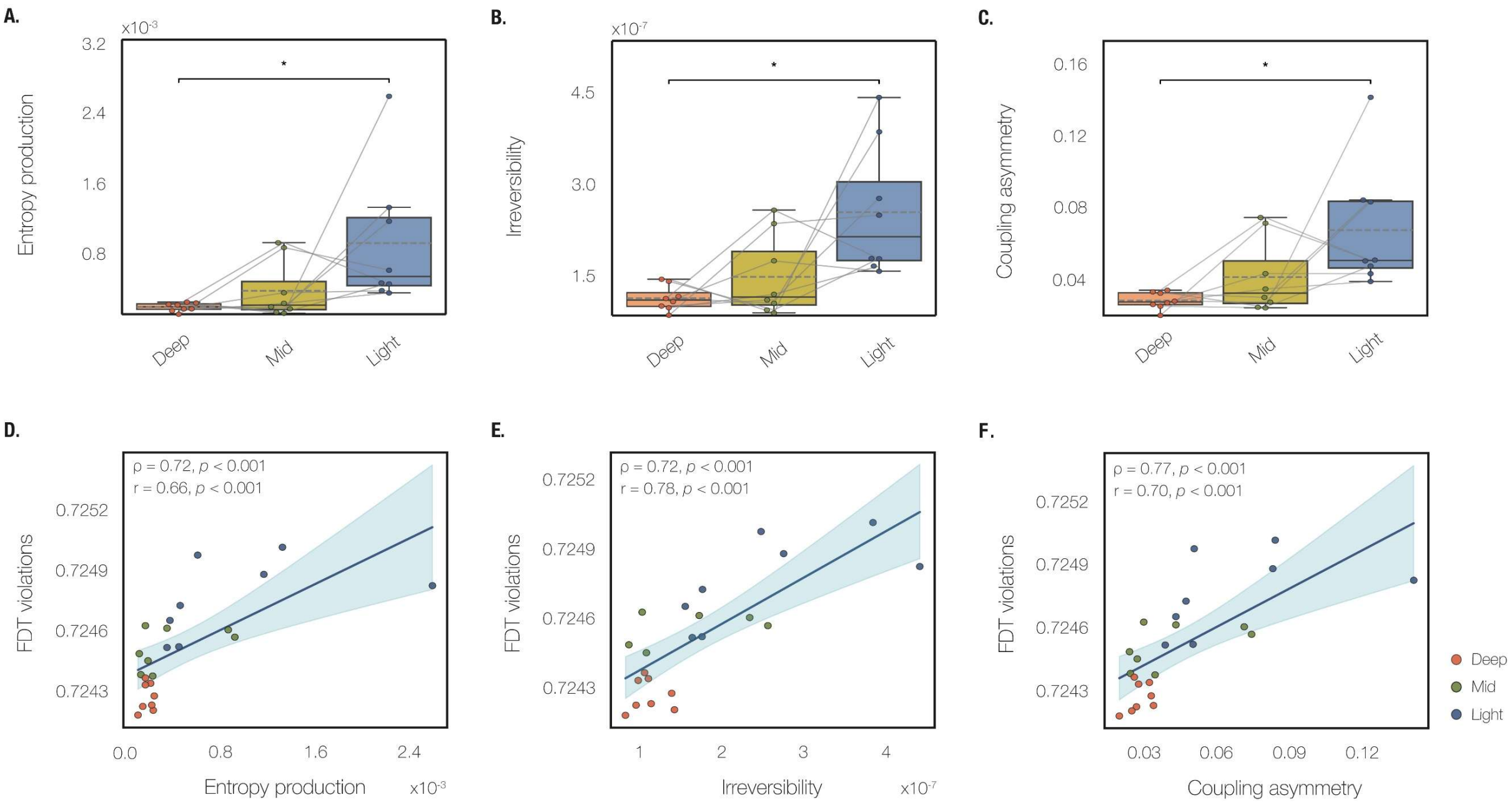

Figure S4. **FDT violations and complementary non-equilibrium measures track levels of anesthesia in mice. (A)** Entropy production rate (EPR), estimated from the whole-brain model fitted to spontaneous activity across graded anesthesia levels. **(B)** Temporal irreversibility computed from the asymmetry of time-lagged covariances in the fitted linear stochastic model. **(C)** Coupling asymmetry, quantified by irreciprocity, derived from the fitted effective coupling matrix. **(D)** FDT violations were positively associated with EPR (Spearman's $\rho = 0.72$, $p < 0.001$; Pearson's $r = 0.66$, $p < 0.001$). **(E)** FDT violations were positively associated with temporal irreversibility (Spearman's $\rho = 0.72$, $p < 0.001$; Pearson's $r = 0.78$, $p < 0.001$). **(F)** FDT violations were positively associated with coupling asymmetry (Spearman's $\rho = 0.77$, $p < 0.001$; Pearson's $r = 0.70$, $p < 0.001$). Across measures, deeper anesthesia was associated with reduced non-equilibrium dynamics. Pairwise comparisons were performed using Wilcoxon signed-rank tests with Holm-Bonferroni correction. * $p < 0.05$

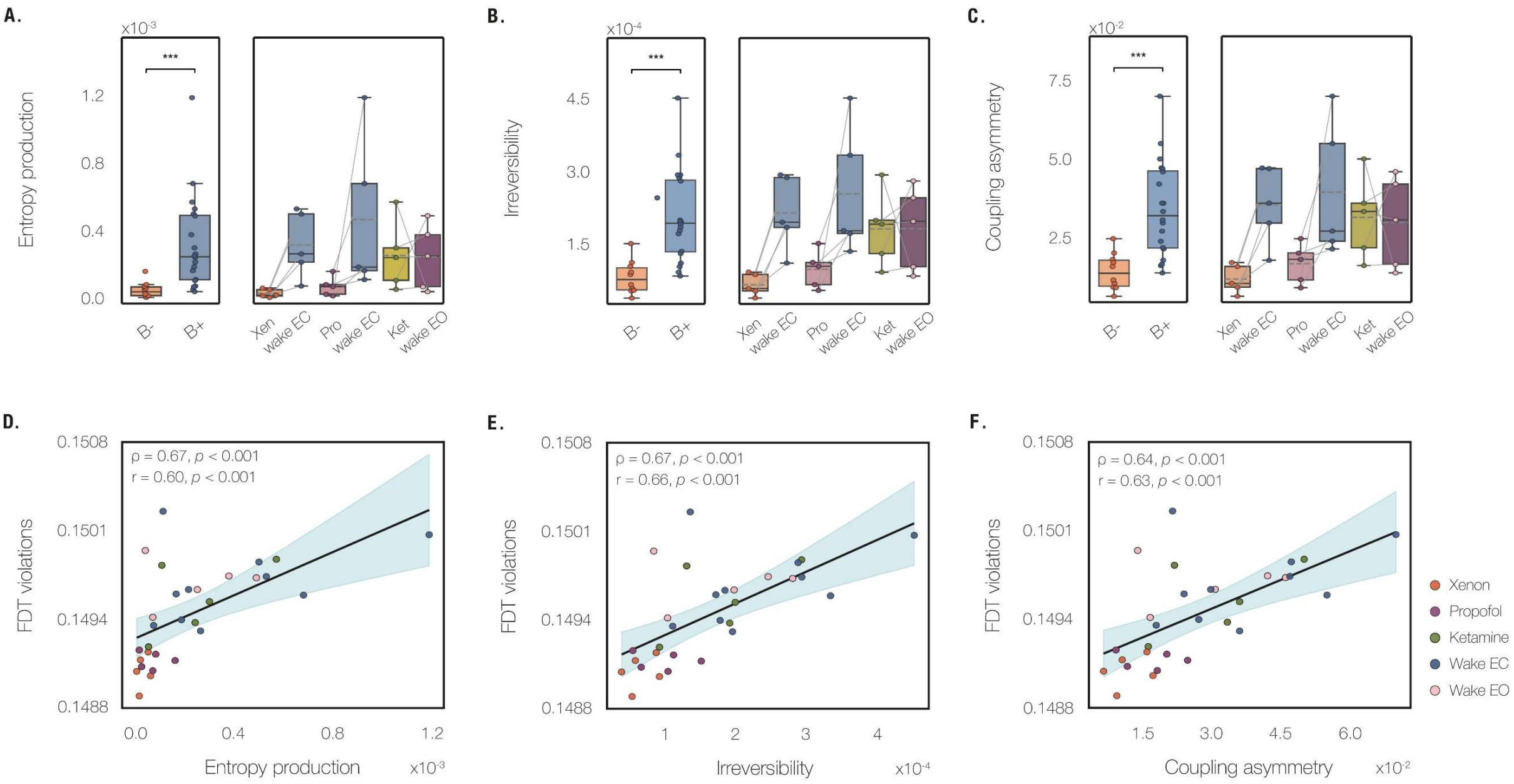

Figure S5. **FDT violations and non-equilibrium measures across wakefulness and anesthesia in humans. (A)** Entropy production rate across anesthetic conditions and grouped based on participants that reported conscious experience (wakefulness and ketamine; B+) and those who did not (xenon and propofol; B-). **(B)** Temporal irreversibility computed from the fitted linear stochastic model across conditions. **(C)** Coupling asymmetry, quantified by irreciprocity, across conditions. **(D)** FDT violations were positively associated with EPR (Spearman's ρ

= 0.67, $p < 0.001$; Pearson's r = 0.60, $p < 0.001$). **(E)** FDT violations were positively associated with temporal irreversibility (Spearman's ρ = 0.67, $p < 0.001$; Pearson's r = 0.66, $p < 0.001$). **(F)** FDT violations were positively associated with coupling asymmetry (Spearman's ρ = 0.64, $p < 0.001$; Pearson's r = 0.63, $p < 0.001$). Xenon and propofol showed reduced non-equilibrium dynamics relative to wakefulness, whereas ketamine remained closer to wakefulness, in line with the main manuscript results for PCI and FDT violations. *** $p < 0.001$

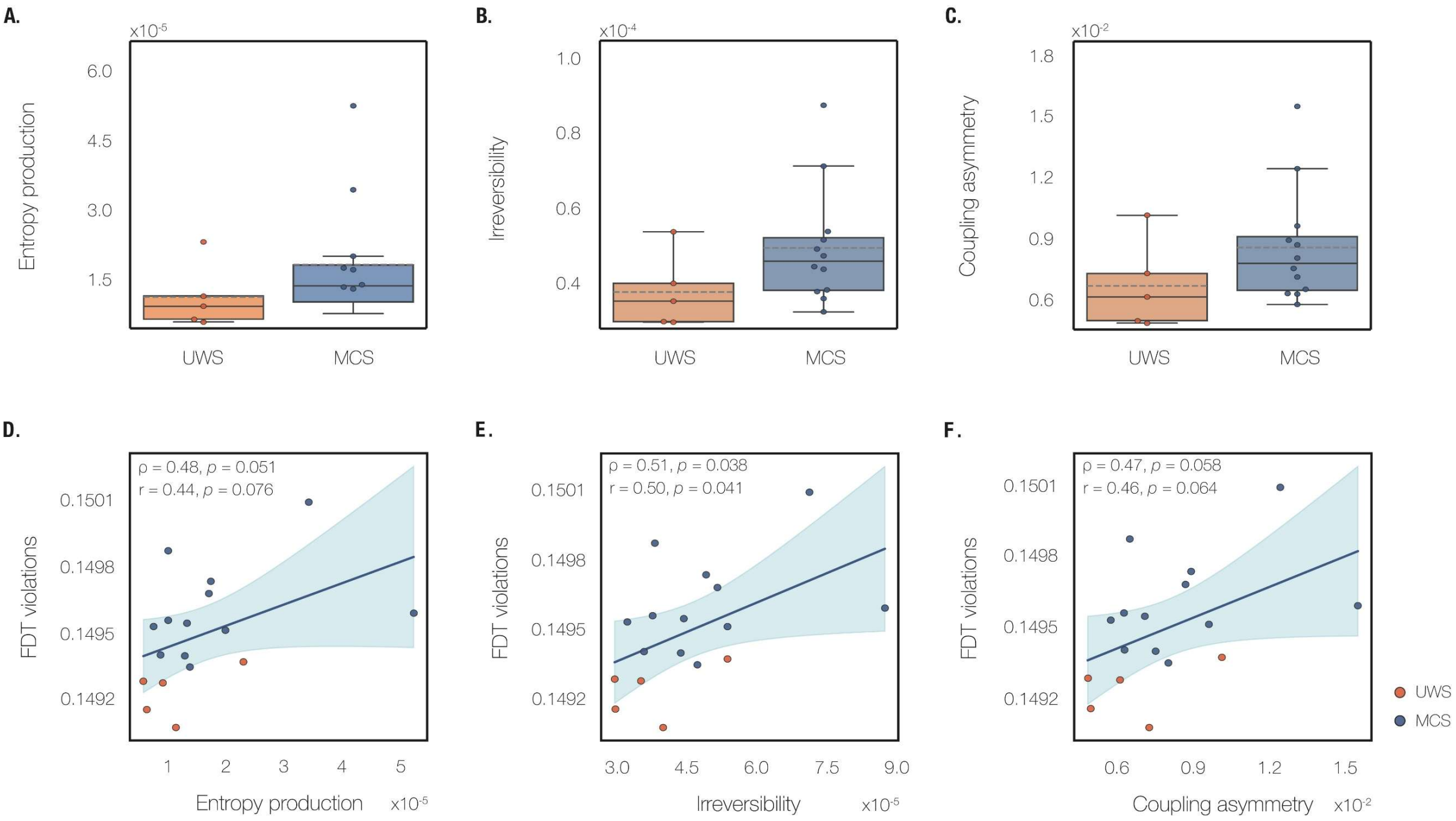

Figure S6. **FDT violations and non-equilibrium measures in disorders of consciousness. (A)** Entropy production rate (EPR) in patients with and without behavioral signs of consciousness. **(B)** Temporal irreversibility across diagnostic groups. **(C)** Coupling asymmetry, quantified by irreciprocity, across diagnostic groups. **(D)** Association between FDT violations and EPR (Spearman's ρ = 0.48, $p = 0.051$; Pearson's r = 0.44, $p = 0.0762$). **(E)** Association between FDT violations and temporal irreversibility (Spearman's ρ = 0.51, $p = 0.038$; Pearson's r = 0.50, $p = 0.0412$). **(F)** Association between FDT violations and coupling asymmetry (Spearman's ρ = 0.47, $p = 0.0583$; Pearson's r = 0.46, $p = 0.0643$). All three non-equilibrium measures showed lower values in UWS than in MCS−/MCS+, mirroring the pattern observed for PCI and FDT violations. However, only temporal irreversibility showed a significant positive association with FDT violations in the pooled DoC cohort, whereas EPR and coupling asymmetry showed positive trend-level associations.

Supplementary Note 5. Extension of human EEG experimental design and data curation

Electrode layouts and montages

Figure S7 illustrates the electrode layouts and digitized montages used for the human EEG recordings in two experimental setups. Figure S7A shows the digitized electrode positions for the Nexstim EEG system (Nexstim Plc.), which consists of 60 channels. Figure S7B displays the digitized layout for the BrainAmp EEG system (Brain Products GmbH), comprising 64 channels and used in a small subset of DoC recordings.

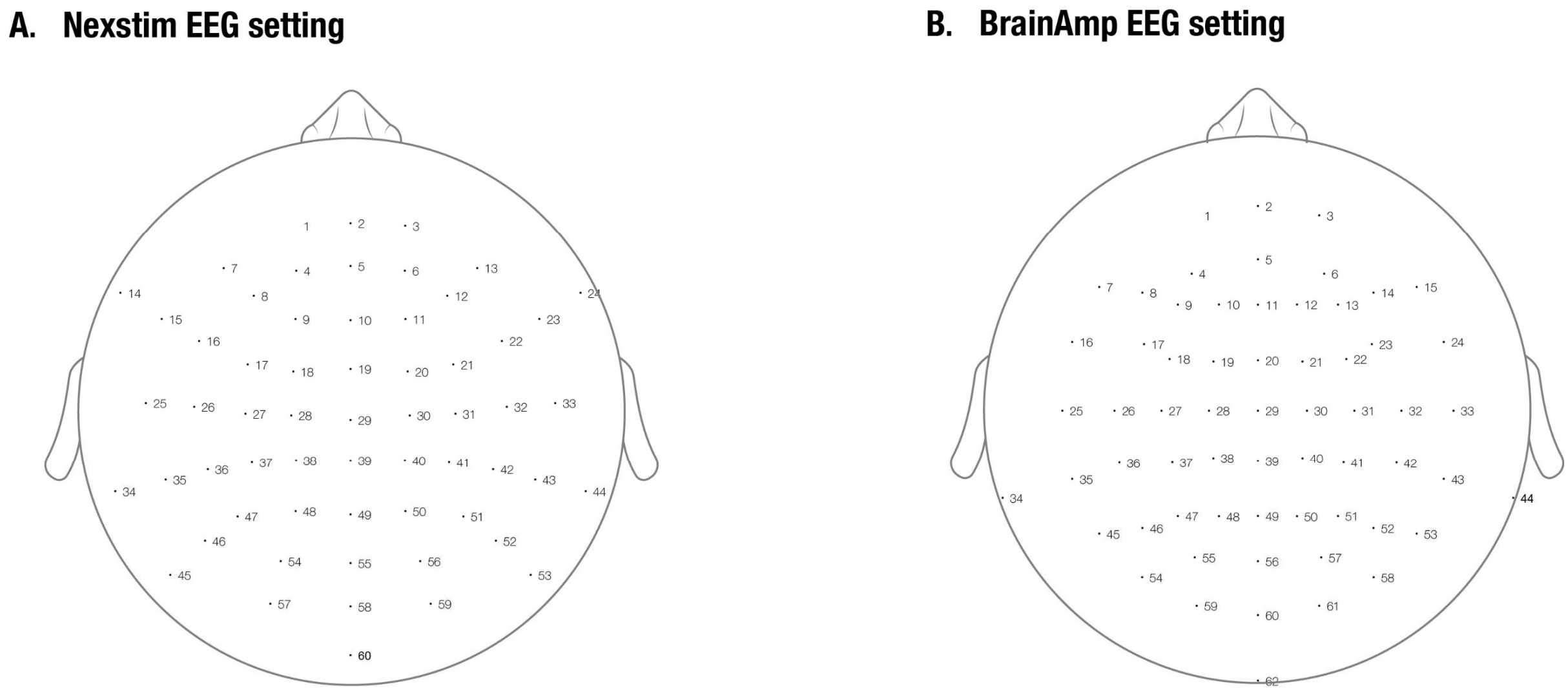

Figure S7. **Human EEG electrode layouts and montages. (A)** Digitization of the electrode positions for the Nexstim EEG system (Nexstim Plc., 60 channels). (**B)** Digitization of the electrode positions for the BrainAmp EEG system (Brain Products GmbH, 64 channels). Note the use of an external reference electrode on the forehead.

Drugs and exclusion criteria

Patients with DoC continued their standard pharmacological treatments during the EEG recording period. Specifically, antiepileptic medications were prescribed to 8 of the 17 patients. All patients in a chronic or persistent DoC state were free from sedative medications, while acute patients had been off sedatives for at least seven days prior to recording. Patients in a comatose state were excluded. Accordingly, no patient exhibited a flat or burst-suppression EEG pattern. Inclusion required at least one valid TMS-EEG session acquired either concurrently with, or in close temporal proximity to, a resting-state EEG recording.

Independent component analysis for the identification and removal of physiological artifacts

To ensure the stability of the Independent Component Analysis (ICA), we first reduced the dimensionality of the EEG data using eigenvalue decomposition, taking into account the number of rejected electrodes. Artifactual components were identified through visual inspection of each component's time series, power spectral density (PSD), and topographical distribution derived from the forward model. Ocular artifacts were typically characterized by pronounced temporal transients resembling eye movements or blinks, a PSD with elevated power at low frequencies and a steep spectral decay, and a prefrontal topography—often showing bilateral asymmetry for horizontal movements and symmetry for vertical movements and blinks. Muscular artifacts were identified by their fast, spiky temporal dynamics, occasionally organized into bursts, a broad high-frequency elevation in the PSD, and a topography indicative of localized superficial dipoles. Cardiac components were recognized by the periodic appearance of R-wave-like deflections in the time domain, a saw-tooth spectral shape associated with non-sinusoidal activity around 1 Hz, and, in some cases, a temporo-occipital distribution.

ICA-based cleaning was essential to reduce potential biases. Electrooculographic and sweat artifacts can increase low-frequency power, mimicking pathological slowing of brain activity, while electromyographic artifacts can artificially elevate power in the beta and gamma ranges, giving the false impression of increased fast cortical activity. Further details can be found in the original publications describing these datasets [S11, S12, S18, S19].

Post-anoxic subgroup: clinical and recording characteristics

The post-anoxic subgroup included 9 patients, of whom 5 were diagnosed with UWS and 4 with MCS, and underwent the same acquisition, preprocessing, PCI computation, and FDT estimation procedures as the primary non-anoxic DoC cohort. Short spontaneous EEG recordings were obtained either during the same TMS-EEG session (N = 8) or, when necessary, during a closely timed session (N = 1 within 2 weeks). Estimation of FDT violations was performed blind to behavioral diagnosis, PCI values, and other clinical information. EEG was recorded using Ag/AgCl electrodes and TMS-compatible amplifiers (Nexstim Plc., 60 channels, N = 9). When applicable, patients remained on their standard clinical treatments, including antiepileptic medication in 4 cases. This subgroup is reported separately because severe post-anoxic injury is frequently associated with diffuse cortical damage and low-voltage EEG, features that are important for interpreting both PCI and FDT violations.

Supplementary Note 6. FDT violations in the broadband range in animal models

To validate the focus on SO in the main analysis, which accounts for up to 95% of the spectral power, we repeated the analyses using broadband LFP signals within the same frequency range used for human EEG data. Overall, the results were qualitatively consistent with those obtained in the SO range, although the associations were somewhat noisier.

Broadband FDT violations also significantly distinguished all three levels of anesthesia (Figure S8A), as supported by a strong monotonic relationship with anesthesia depth (Spearman's $\rho = 0.870$, $p < 0.001$).

Broadband FDT violations were also significantly correlated with PCI (Spearman's $\rho = 0.69$, $p < 0.001$; Pearson's $r = 0.62$, $p = 0.00132$; Figure S8B). An ANCOVA including the condition interaction term revealed no evidence of slope heterogeneity between groups, indicating that the relationship was conserved across conditions ($F(2,18) = 0.114$, $p = 0.8933$; Freedman–Lane permutation $p_{perm} = 0.8945$). Leave-one-subject-out cross-validation further supported out-of-sample generalization (Spearman's $\rho = 0.6435$, $p < 0.001$; Pearson's $r = 0.541$, $p = 0.0063$). These results indicate that restricting the main analysis to SO does not create the observed relationship but rather enhances its sensitivity and interpretability in this dataset.

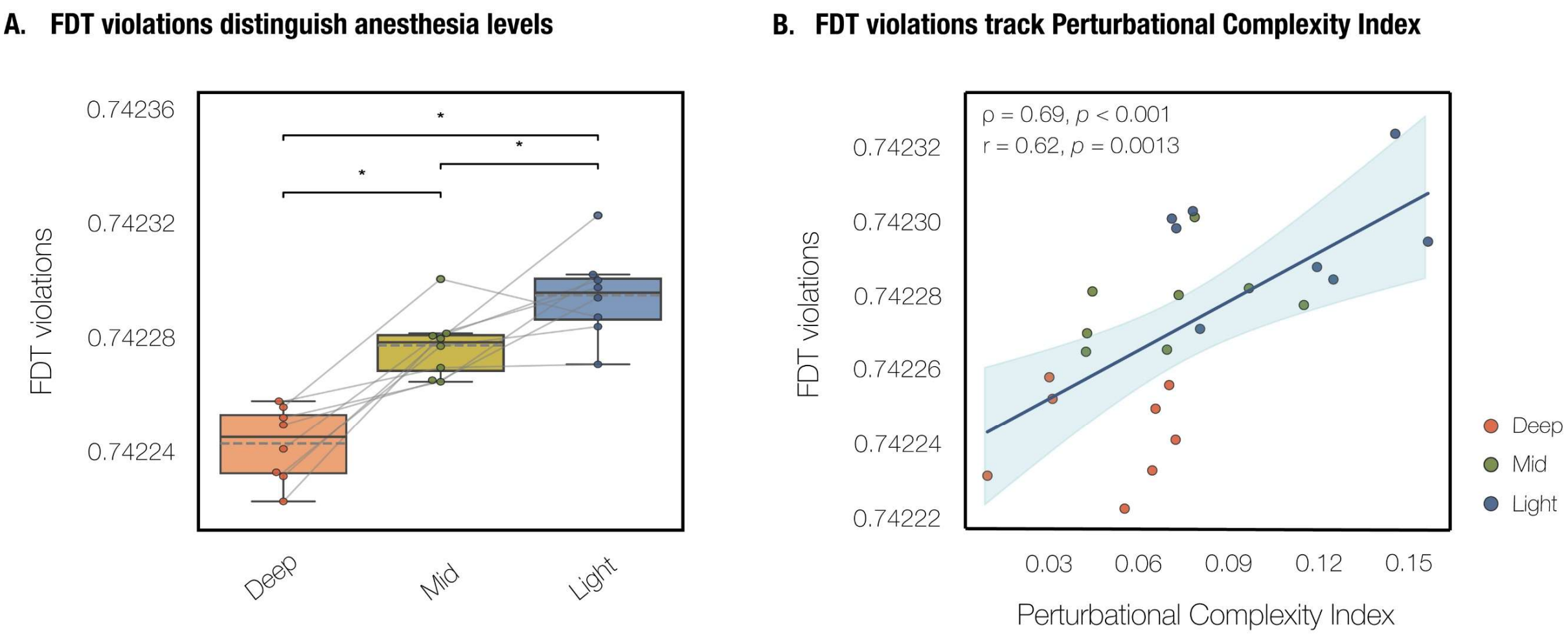

Figure S8. **FDT violations track levels of anesthesia in mice and correlate with the PCI in the broadband range. (A)** Broadband FDT violations, calculated from the whole-brain model fitted to empirical spontaneous activity, discriminated among all the conditions, as indicated by a strong monotonic relationship (Spearman's $\rho = 0.870$, $p < 0.001$). **(B)** Broadband FDT violations significantly correlated with PCI (Spearman's $\rho = 0.69$, $p < 0.001$; Pearson's $r = 0.62$, $p = 0.00132$). An ANCOVA including the condition interaction term revealed no evidence of slope heterogeneity between groups, indicating that the relationship was conserved across conditions ($F(2,18) = 0.114$, $p = 0.8933$; Freedman–Lane permutation $p_{perm} = 0.8945$). Leave-one-subject-out cross-validation supported out-of-sample generalization (Spearman's $\rho = 0.6435$, $p < 0.001$; Pearson's $r = 0.541$, $p = 0.0063$). All the pairwise comparisons were performed using Wilcoxon signed-rank tests and corrected post hoc with Holm-Bonferroni. * $p < 0.05$

Supplementary Note 7. Illustrative TMS-EEG traces for PCI computation

PCI values were obtained from previous studies [S11, S12, S20, S21]. Here, we show two representative TMS–EEG responses used for PCI computation (Figure S9). One example corresponds to a high PCI value, reflecting rich and differentiated evoked responses, whereas the other corresponds to a low PCI value, indicating a simpler and more stereotyped spatiotemporal response pattern.

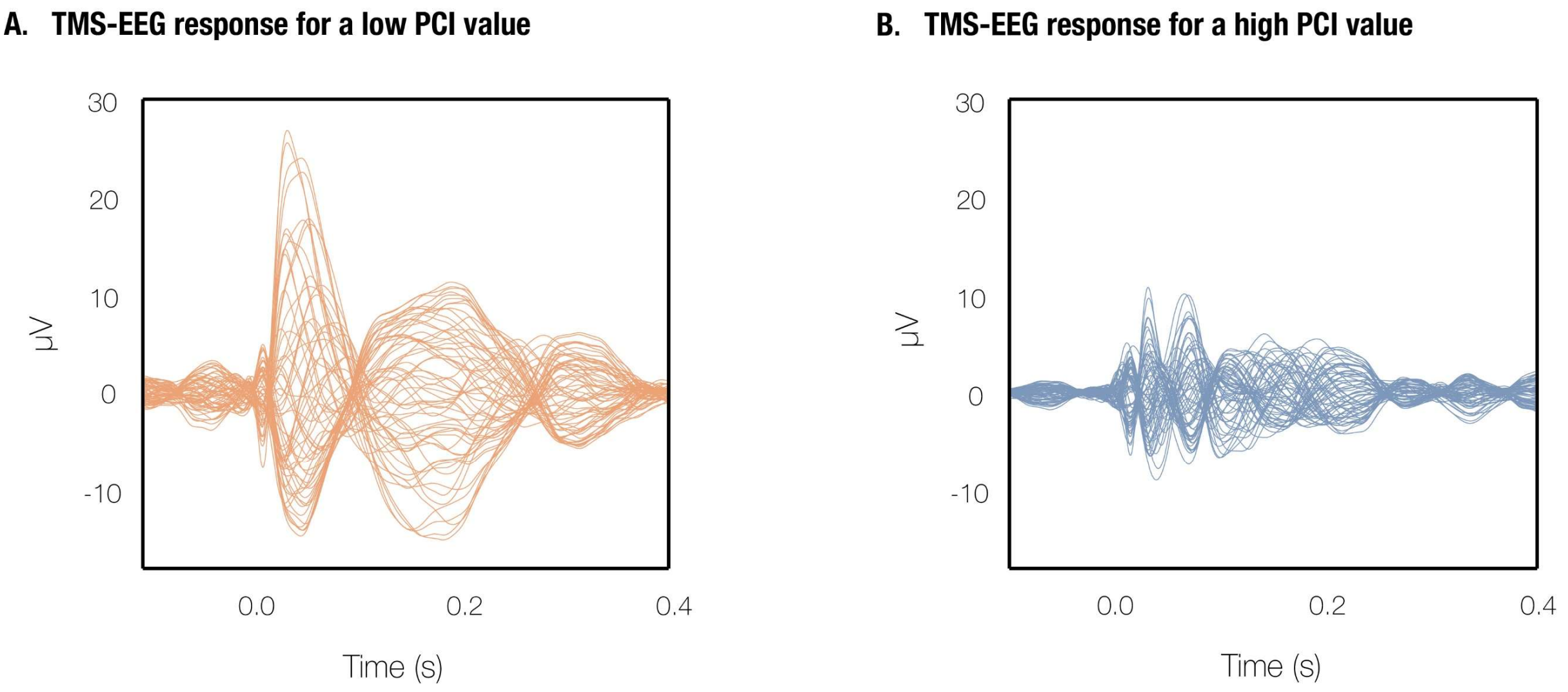

Figure S9. **TMS-EEG responses for PCI computation. (A)** Example of a TMS–EEG response with low complexity and a more stereotyped spatiotemporal pattern, corresponding to a low PCI value. **(B)** Example of a TMS–EEG response with rich and differentiated spatiotemporal dynamics, corresponding to a high PCI value.

Supplementary Note 8. Local weak stationarity control analyses

To evaluate the stationarity assumption underlying the multivariate Ornstein-Uhlenbeck framework, we performed additional epoch-based control analyses on signals subjected to the same core preprocessing used for the main FDT violation analysis. After preprocessing, recordings were segmented into 10-s windows with 50% overlap. Within each epoch, the mean and linear trend were removed. Local weak stationarity was then assessed using complementary Augmented Dickey-Fuller (ADF) and Kwiatkowski–Phillips–Schmidt–Shin (KPSS) tests, both evaluated at $\alpha = 0.05$. Epochs were classified as quasi-stationary when the ADF test rejected the null hypothesis of a unit root and the KPSS test failed to reject the null hypothesis of stationarity. These quantities were then summarized at the subject level, rather than treating overlapping epochs as independent observations.

Across the main datasets, the proportion of channel-epochs classified as quasi-stationary was high. In the mouse LFP dataset, the median (IQR) percentage of quasi-stationary channel-epochs was 100.0 (100.0–100.0) % in light anesthesia, 100.0 (99.9–100.0) % in mid anesthesia, and 99.4 (98.9–99.8) % in deep anesthesia. In the human anesthesia EEG dataset, the corresponding values were 100.0 (100.0–100.0) % in wakefulness with eyes closed, 100.0 (100.0–100.0) % in wakefulness with eyes open, 98.3 (90.8–100.0) % in xenon, 98.4 (97.9–99.5) % in propofol, and 99.8 (99.7–100.0) % in ketamine. In the non-anoxic DoC dataset, the values were 99.9 (99.7–100.0) % in MCS and 99.3 (97.6–99.7) % in UWS.

In the post-anoxic subgroup, the percentage of quasi-stationary channel-epochs was likewise high, reaching 99.8 (99.5–100.0) % in MCS and 99.7 (98.9–99.9) % in UWS. These values are reported for completeness, but this subgroup should be interpreted cautiously as a boundary condition given the low-voltage EEG patterns, limited sample size, and physiologically constrained nature of the recordings.

Overall, these results indicate that, after detrending and epoching, the large majority of analyzed segments were compatible with a local weak stationary approximation across all datasets. At the same time, these tests do not imply that the recordings form a single perfectly stationary regime over their full duration, nor do they exclude transient metastability or hidden state transitions. Rather, they support the use of a local weak stationarity approximation for the effective linear model.

Supplementary Note 9. Homogeneous noise in a linear Langevin system

In the case of spatially homogeneous noise, the normalized stationary-state autocorrelation does not depend on the value of the noise amplitude σ [S13]. Therefore, this parameter does not need to be fitted, as it does not provide additional information under the present modeling assumptions [S22]. In what follows, we provide proof of this.

Consider that the stationary-state autocorrelation function is given by:

$$C(\tau) = \langle x(t+\tau)x^T(t) \rangle \quad (S6)$$

Under stationary conditions, we have:

$$x(t) = \int_{-\infty}^{t} e^{-B(t-s)} \eta(s) ds \quad (S7)$$

$$x(t+\tau) = \int_{-\infty}^{t+\tau} e^{-B(t+\tau-s)} \eta(s) ds \quad (S8)$$

From $C(\tau)$, the normalized stationary-state autocorrelation function for each node or brain region i can be derived as:

$$\frac{C_{ii}(\tau)}{C_{ii}(0)} = \frac{[e^{-B\tau}K]_{ii}}{K_{ii}} \quad (S9)$$

When the noise amplitude is identical across all brain regions or nodes i (i.e. $\sigma_i = \sigma$), and denoting the identity matrix by I, the noise covariance is:

$$Q = 2\sigma^2 I \quad (S10)$$

At stationarity, the covariance matrix K satisfies the continuous-time Lyapunov equation:

$$BK + KB^T = Q \quad (S11)$$

Since this equation is linear in the noise variance, its solution can be written as $K = \sigma^2 K_0$, where K_0 satisfies $BK_0 + K_0 B^T = 2I$. Thus, the normalized autocorrelation function for component i becomes:

$$\frac{C_{ii}(\tau)}{C_{ii}(0)} = \frac{[e^{-B\tau}\sigma^2 K_0]_{ii}}{[\sigma^2 K_0]_{ii}} = \frac{[e^{-B\tau}K_0]_{ii}}{[K_0]_{ii}} \quad (S12)$$

From Equation S12, we can clearly see that in the case of homogeneous noise, the normalized stationary-state autocorrelation does not depend on the noise amplitude. Therefore, there is no need to fit the noise amplitude in the model, as all relevant information is embedded in the generative effective connectivity matrix B.

SUPPLEMENTAL REFERENCES

[S1] Massimini, M., Ferrarelli, F., Huber, R., Esser, S.K., Singh, H., and Tononi, G. (2005). Breakdown of Cortical Effective Connectivity during Sleep. Science *309*, 2228–2232.

[S2] Forgacs, P.B., Conte, M.M., Fridman, E.A., Voss, H.U., Victor, J.D., and Schiff, N.D. (2014). Preservation of electroencephalographic organization in patients with impaired consciousness and imaging-based evidence of command-following. Ann. Neurol. *76*, 869–879. https://doi.org/10.1002/ana.24283.

[S3] Andrillon, T., Poulsen, A.T., Hansen, L.K., Léger, D., and Kouider, S. (2016). Neural Markers of Responsiveness to the Environment in Human Sleep. J. Neurosci. *36*, 6583–6596. https://doi.org/10.1523/JNEUROSCI.0902-16.2016.

[S4] Edlow, B.L., Claassen, J., Schiff, N.D., and Greer, D.M. (2021). Recovery from disorders of consciousness: mechanisms, prognosis and emerging therapies. Nat. Rev. Neurol. *17*, 135–156. https://doi.org/10.1038/s41582-020-00428-x.

[S5] Sanchez-Vives, M.V., Massimini, M., and Mattia, M. (2017). Shaping the Default Activity Pattern of the Cortical Network. Neuron *94*, 993–1001. https://doi.org/10.1016/j.neuron.2017.05.015.

[S6] D'Andola, M., Rebollo, B., Casali, A.G., Weinert, J.F., Pigorini, A., Villa, R., Massimini, M., and Sanchez-Vives, M.V. (2018). Bistability, Causality, and Complexity in Cortical Networks: An In Vitro Perturbational Study. Cereb. Cortex *28*, 2233–2242. https://doi.org/10.1093/cercor/bhx122.

[S7] Cavelli, M.L., Mao, R., Findlay, G., Driessen, K., Bugnon, T., Tononi, G., and Cirelli, C. (2023). Sleep/wake changes in perturbational complexity in rats and mice. iScience *26*, 106186. https://doi.org/10.1016/j.isci.2023.106186.

[S8] Pigorini, A., Sarasso, S., Proserpio, P., Szymanski, C., Arnulfo, G., Casarotto, S., Fecchio, M., Rosanova, M., Mariotti, M., Lo Russo, G., et al. (2015). Bistability breaks-off deterministic responses to intracortical stimulation during non-REM sleep. NeuroImage *112*, 105–113. https://doi.org/10.1016/j.neuroimage.2015.02.056.

[S9] Rosanova, M., Fecchio, M., Casarotto, S., Sarasso, S., Casali, A.G., Pigorini, A., Comanducci, A., Seregni, F., Devalle, G., Citerio, G., et al. (2018). Sleep-like cortical OFF-periods disrupt causality and complexity in the brain of unresponsive wakefulness syndrome patients. Nat. Commun. *9*, 4427. https://doi.org/10.1038/s41467-018-06871-1.

[S10] Massimini, M., Corbetta, M., Sanchez-Vives, M.V., Andrillon, T., Deco, G., Rosanova, M., and Sarasso, S. (2024). Sleep-like cortical dynamics during wakefulness and their network effects following brain injury. Nat. Commun. *15*, 7207. https://doi.org/10.1038/s41467-024-51586-1.

[S11] Colombo, M.A., Comanducci, A., Casarotto, S., Derchi, C.-C., Annen, J., Viganò, A., Mazza, A., Trimarchi, P.D., Boly, M., Fecchio, M., et al. (2023). Beyond alpha power: EEG spatial and spectral gradients robustly stratify disorders of consciousness. Cereb. Cortex *33*, 7193–7210. https://doi.org/10.1093/cercor/bhad031.

[S12] Casarotto, S., Comanducci, A., Rosanova, M., Sarasso, S., Fecchio, M., Napolitani, M., Pigorini, A., G. Casali, A., Trimarchi, P.D., Boly, M., et al. (2016). Stratification of unresponsive patients by an independently validated index of brain complexity. Ann. Neurol. *80*, 718–729. https://doi.org/10.1002/ana.24779.

[S13] Godrèche, C., and Luck, J.-M. (2019). Characterising the nonequilibrium stationary states of Ornstein-Uhlenbeck processes. J. Phys. Math. Theor. *52*, 035002. https://doi.org/10.1088/1751-8121/aaf190.

[S14] Deco, G., Sanz Perl, Y., Bocaccio, H., Tagliazucchi, E., and Kringelbach, M.L. (2022). The INSIDEOUT framework provides precise signatures of the balance of intrinsic and extrinsic dynamics in brain states. Commun. Biol. *5*. https://doi.org/10.1038/s42003-022-03505-7.

[S15] Ohga, N., Ito, S., and Kolchinsky, A. (2023). Thermodynamic Bound on the Asymmetry of Cross-Correlations. Phys. Rev. Lett. *131*, 077101. https://doi.org/10.1103/PhysRevLett.131.077101.

[S16] Deco, G., Lynn, C.W., Sanz Perl, Y., and Kringelbach, M.L. (2023). Violations of the fluctuation-dissipation theorem reveal distinct nonequilibrium dynamics of brain states. Phys. Rev. E *108*, 064410. https://doi.org/10.1103/PhysRevE.108.064410.

[S17] Nartallo-Kaluarachchi, R., Asllani, M., Deco, G., Kringelbach, M.L., Goriely, A., and Lambiotte, R. (2024). Broken detailed balance and entropy production in directed networks. Phys. Rev. E *110*, 034313. https://doi.org/10.1103/PhysRevE.110.034313.

[S18] Colombo, M.A., Napolitani, M., Boly, M., Gosseries, O., Casarotto, S., Rosanova, M., Brichant, J.-F., Boveroux, P., Rex, S., Laureys, S., et al. (2019). The spectral exponent of the resting EEG indexes the presence of consciousness during unresponsiveness induced by propofol, xenon, and ketamine. NeuroImage *189*, 631–644. https://doi.org/10.1016/j.neuroimage.2019.01.024.

[S19] Massimini, M., and Laureys, S. (2017). Rest EEG recordings in healthy subjects during wakefulness, sleep and anesthesia with ketamine, propofol, and xenon. (Zenodo). https://doi.org/10.5281/ZENODO.806176.

[S20] Sarasso, S., Boly, M., Napolitani, M., Gosseries, O., Charland-Verville, V., Casarotto, S., Rosanova, M., Casali, A.G., Brichant, J.-F., Boveroux, P., et al. (2015). Consciousness and Complexity during Unresponsiveness Induced by Propofol, Xenon, and Ketamine. Curr. Biol. *25*, 3099–3105. https://doi.org/10.1016/j.cub.2015.10.014.

[S21] Dasilva, M., Camassa, A., Navarro-Guzman, A., Pazienti, A., Perez-Mendez, L., Zamora-López, G., Mattia, M., and Sanchez-Vives, M.V. (2021). Modulation of cortical slow oscillations and complexity across anesthesia levels. NeuroImage *224*, 117415. https://doi.org/10.1016/j.neuroimage.2020.117415.

[S22] Monti, J.M., Perl, Y.S., Tagliazucchi, E., Kringelbach, M.L., and Deco, G. (2025). Fluctuation-dissipation theorem and the discovery of distinctive off-equilibrium signatures of brain states. Phys. Rev. Res. *7*, 013301. https://doi.org/10.1103/PhysRevResearch.7.013301.
