# Supplementary material for: Thermodynamics of consciousness: Non-equilibrium brain dynamics track conscious states
Source: Cell Rep. 2026 Jul 31;45(8):117782. doi: 10.1016/j.celrep.2026.117782 (PMC13506674; doi:10.1016/j.celrep.2026.117782)
Supplement: Supplementary file 2 [file mmc2.pdf]

Cell Reports

Article

Thermodynamics of consciousness: Non-equilibrium brain dynamics track conscious states

Graphical abstract

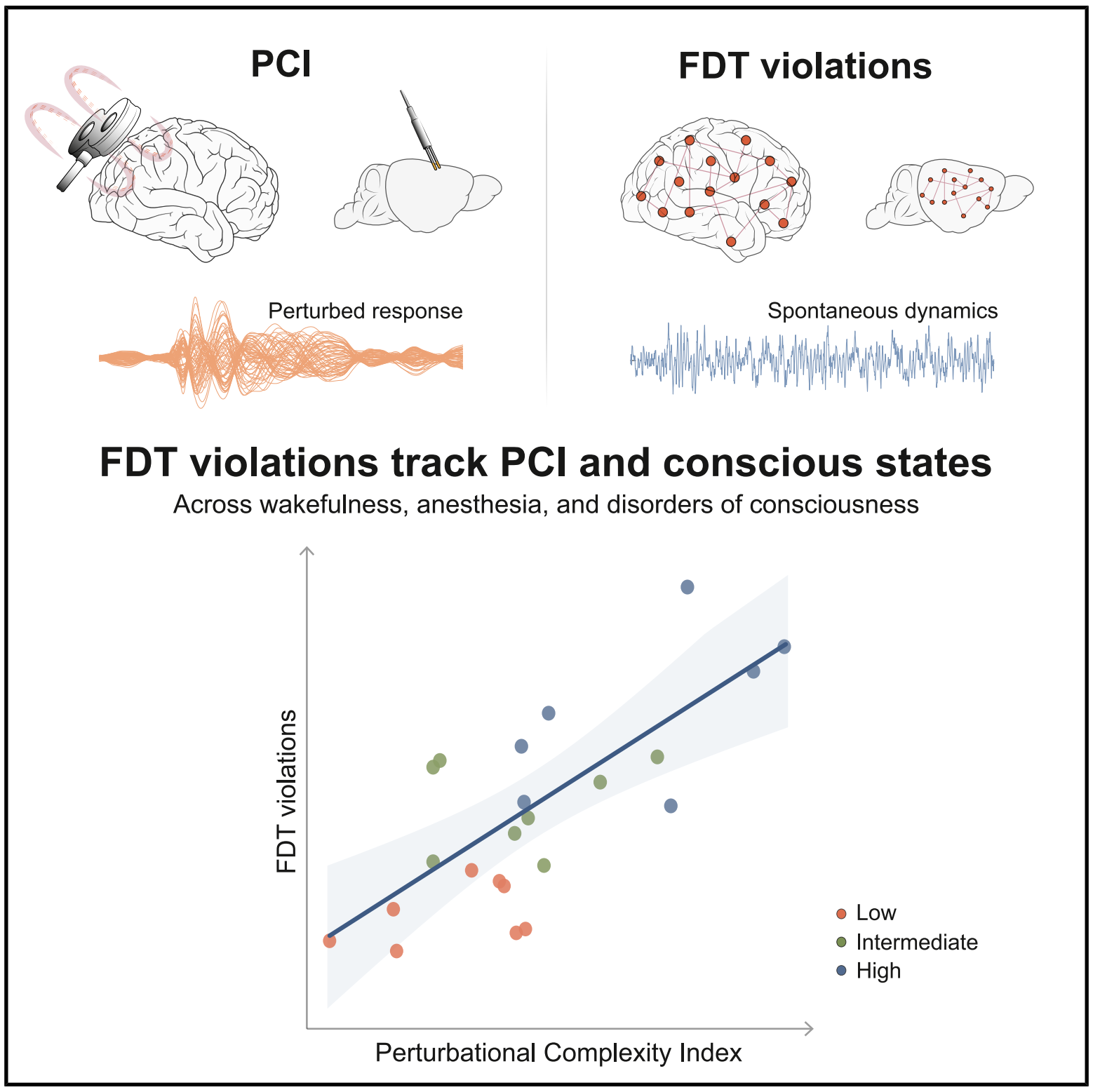

Authors

Tomas Berjaga-Buisan, Juan Manuel Monti, Martina Cortada, ..., Marcello Massimini, Yonatan Sanz Perl, Gustavo Deco

Correspondence

tomas.berjaga@upf.edu

In brief

Berjaga-Buisan et al. provide direct empirical evidence that perturbational complexity is linked to non-equilibrium dynamics in spontaneous brain activity. Across mice and humans, fluctuation-dissipation theorem (FDT) violations track perturbational complexity index (PCI) and conscious states, establishing a bridge between spontaneous fluctuations, perturbational complexity, and consciousness across experimental and clinical conditions.

Highlights

- Empirical PCI covaries with spontaneous non-equilibrium brain dynamics across species
- FDT violations track graded anesthesia in mice
- FDT violations distinguish wakefulness and anesthesia in humans
- FDT violations distinguish DoC groups and correlate with clinical scores

Berjaga-Buisan et al., 2026, Cell Reports *45*, 117782
August 25, 2026 © 2026 The Authors. Published by Elsevier Inc.
https://doi.org/10.1016/j.celrep.2026.117782

Cell Reports

CellPress OPEN ACCESS

Article

Thermodynamics of consciousness: Non-equilibrium brain dynamics track conscious states

Tomas Berjaga-Buisan,[1,18,*] Juan Manuel Monti,[2,3] Martina Cortada,[4,5] Michele A. Colombo,[6] Sebastian M. Geli,[1] Gianluca Gaglioti,[6] Simone Sarasso,[6] Morten L. Kringelbach,[7,8,9,10] Maurizio Corbetta,[11,12,13] Maria V. Sanchez-Vives,[4,14] Marcello Massimini,[6,15] Yonatan Sanz Perl,[10,16,17] and Gustavo Deco[10,14,17]

[1]Center for Brain and Cognition, Computational Neuroscience Group, Department of Engineering, Universitat Pompeu Fabra, 08005 Barcelona, Spain
[2]Instituto de Física Rosario CONICET-UNR, Rosario 2000, Argentina
[3]Laboratorio de Colisiones Atómicas, FCEIA, Universidad Nacional de Rosario, Rosario 2000, Argentina
[4]Institut d'Investigacions Biomèdiques August Pi i Sunyer (IDIBAPS), 08036 Barcelona, Spain
[5]Facultat de Física, Universitat de Barcelona (UB), 08028 Barcelona, Spain
[6]Department of Biomedical and Clinical Sciences, Università degli Studi di Milano, 20157 Milan, Italy
[7]Centre for Eudaimonia and Human Flourishing, Linacre College, University of Oxford, Oxford OX3 9BX, UK
[8]Department of Psychiatry, University of Oxford, Oxford OX3 7JX, UK
[9]Center for Music in the Brain, Department of Clinical Medicine, Aarhus University, 8000 Aarhus C, Denmark
[10]International Centre for Flourishing, Universities of Oxford (UK), Aarhus (Denmark) and Pompeu Fabra (Spain), Oxford OX3 9BX, UK
[11]Padova Neuroscience Center (PNC), University of Padova, 35129 Padova, Italy
[12]Department of Neuroscience, University of Padova, 35128 Padova, Italy
[13]Venetian Institute of Molecular Medicine (VIMM), 35129 Padova, Italy
[14]Institució Catalana de Recerca i Estudis Avançats (ICREA), 08010 Barcelona, Spain
[15]IRCCS, Fondazione Don Carlo Gnocchi Onlus, 20148 Milan, Italy
[16]Department of Engineering, Universidad de San Andrés, Buenos Aires B1644BID, Argentina
[17]Center for Brain and Cognition, Computational Neuroscience Group, Faculty of Medicine and Life Sciences, Universitat Pompeu Fabra, 08005 Barcelona, Spain
[18]Lead contact
*Correspondence: tomas.berjaga@upf.edu
https://doi.org/10.1016/j.celrep.2026.117782

SUMMARY

The quest for reliable and objective measures of consciousness is critical in basic and clinical neuroscience. Across species, the perturbational complexity index (PCI) has emerged as a robust empirical marker by directly perturbing the brain, yet its relationship to broader physical principles remains unclear. Here, we address this gap by introducing a non-invasive framework based on generative whole-brain models of non-equilibrium brain dynamics. Using these models, we identify violations of the fluctuation-dissipation theorem (FDT) in humans and rodents across wakefulness, anesthesia, and disorders of consciousness (DoC). Mirroring PCI, FDT violations decrease in unresponsive DoC and anesthesia compared with conscious conditions. These findings reveal a robust empirical link between PCI and non-equilibrium dynamics in spontaneous brain signals, suggesting that non-equilibrium dynamics capture an important aspect of perturbational complexity. Overall, this framework opens non-invasive, model-based avenues for understanding consciousness and supports efforts to assess its loss and recovery in health and disease.

INTRODUCTION

Distinguishing between conscious and unconscious brain states remains one of the most fundamental and unresolved challenges in systems and clinical neuroscience. Clinical diagnosis currently relies on a patient's ability to interact with the environment, which is typically assessed using standardized behavioral scales, such as the Coma Recovery Scale–Revised (CRS-R).[1,2] However, the absence of behavioral signs cannot be considered definitive evidence of unconsciousness, as individuals may be unable to interact with the environment due to impairments in motor, sensory, or executive functions.[3–5] States of disconnected consciousness can occur in healthy individuals during dreaming, under general anesthesia, and in cases of severe brain injury.[6–9] Therefore, there is an urgent need for neurophysiological measures capable of characterizing consciousness independently of behavior.

The perturbational complexity index (PCI) is a generalizable non-behavioral marker of consciousness that quantifies the algorithmic complexity of spatiotemporal brain patterns in response to external perturbations, typically delivered via transcranial magnetic stimulation (TMS) in humans or intracortical

This is an open access article under the CC BY license (http://creativecommons.org/licenses/by/4.0/).

electrical stimulation in animal models.[10,11] PCI captures the presence of both functional integration and differentiation by computing the extent and information content of the chain of interactions evoked in the brain by a direct perturbation. These features are collectively referred to as brain complexity, which has been proposed as a fundamental requirement for consciousness in both theoretical models and neuroimaging studies.[12–14] PCI has demonstrated reliability in discriminating consciousness from unconsciousness.[15] Notably, previous studies have reported that PCI generalizes across species, scales, and experimental models, consistently distinguishing between conditions in which the brain state was systematically manipulated.[16,17] Beyond this empirical robustness, PCI can be traced back to theoretical principles, and the neuronal underpinnings of its physiological and pathological alterations have been extensively studied.[16–22] However, its relationship to broader principles of physics remains unclear.

Like other living systems, the brain operates far from thermodynamic equilibrium.[23,24] Neural dynamics are characterized by continuous energy dissipation, broken temporal symmetry, and directional information.[24–28] Therefore, recent advances in stochastic thermodynamics, grounded in non-equilibrium statistical physics, offer a promising framework for studying whole-brain dynamics and addressing the conceptual limitations of complexity measures.[24,29] In this context, these tools are used to quantify departures from equilibrium, rather than to directly measure classical macroscopic quantities such as temperature, heat, or work. In particular, the fluctuation-dissipation theorem (FDT) states that, in thermodynamic equilibrium, a system's response to external perturbations is fully determined by the structure of its spontaneous fluctuations.[30] In non-equilibrium systems such as the brain, this balance no longer holds, and the degree to which FDT is violated can be used to quantify the system's effective departure from equilibrium. Importantly, this violation is a marker of non-equilibrium dynamics and has been proposed as a potential signature of consciousness in previous functional magnetic resonance imaging (fMRI) studies.[31,32]

In line with this perspective, recent model-based studies have suggested a link between simulated perturbational complexity in fMRI and asymmetries in effective connectivity.[33] In the present study, we move beyond this by empirically testing the link between PCI and FDT violations in electrophysiological data, relating PCI from real perturbational recordings to FDT violations estimated from spontaneous activity in the same subjects and conditions. We hypothesize that both may be associated with a common aspect of conscious states: The brain's effective departure from thermodynamic equilibrium. Specifically, we suggest that the brain's capacity to generate complex cortical responses to perturbations in conscious states may be associated with a breakdown in the relationship between spontaneous fluctuations and evoked activity. From this perspective, high PCI values, which reflect rich and differentiated evoked responses, are predicted to coincide with strong FDT violations, indicating that the system output cannot be inferred from its intrinsic activity. Conversely, low PCI values observed in unconscious conditions are expected to reflect weaker violations of FDT and are therefore expected to show a more preserved relationship between spontaneous and evoked activity. Moreover, we hypothesize that FDT violations may provide a complementary, non-invasive, model-based measure associated with conscious state, since they can be estimated from spontaneous activity alone.

To test these hypotheses, we analyzed neurophysiological recordings from animal models and human subjects across multiple levels of consciousness and experimental conditions. Specifically, evoked responses and spontaneous activity were analyzed in mice under three graded levels of anesthesia and in two independent human datasets. Among these two human datasets, one included patients with disorders of consciousness (DoC), encompassing individuals diagnosed with unresponsive wakefulness syndrome (UWS) and minimally conscious states (MCS+, MCS−). The second dataset served as a reference and included neurotypical individuals during wakefulness and under different anesthetic agents, enabling us to test the generalizability of our findings across conscious states and providing a brain-based comparison that does not rely on behavioral responsiveness. PCI was computed from cortical responses to direct perturbations, whereas FDT violations were assessed independently using a whole-brain model fitted to empirical recordings of spontaneous fluctuations.

Across the datasets studied here, FDT violations varied systematically across experimental and clinical states and were associated with PCI across species, modalities, and conditions. We also found a robust correlation that was consistent across species, datasets, and measurement modalities: States with higher PCI values were associated with stronger FDT violations, whereas lower PCI values corresponded to smaller departures from equilibrium.

These findings support a robust link between empirical PCI and non-equilibrium spontaneous dynamics, while introducing FDT violations as a complementary, stimulation-free measure associated with conscious state. Overall, this study provides a framework grounded in non-equilibrium brain dynamics that identifies effective departures from equilibrium as a relevant feature of conscious brain states. This framework offers a complementary perspective on consciousness through non-equilibrium thermodynamics and may open new avenues for assessing consciousness in basic and clinical research, as well as for understanding its neural substrates in both health and disease.

RESULTS

Conscious brain states have been proposed to be associated with stronger deviations from thermodynamic equilibrium, with a theoretical link to cortical complexity and PCI.[24,25] To test this hypothesis, large-scale neurophysiological evoked and spontaneous recordings were analyzed from multiple species, modalities, and levels of consciousness. From these recordings, PCI values were estimated from evoked cortical responses as a measure of complexity, whereas FDT violations were quantified from spontaneous activity to reflect the system's non-equilibrium properties (Figure 1A).

PCI values, derived from TMS-evoked or intracortical responses, were gathered from previous studies.[6,15,34,35] In these datasets, spatiotemporal patterns of cortical source activations were binarized using non-parametric permutations, and the

CellPress
OPEN ACCESS

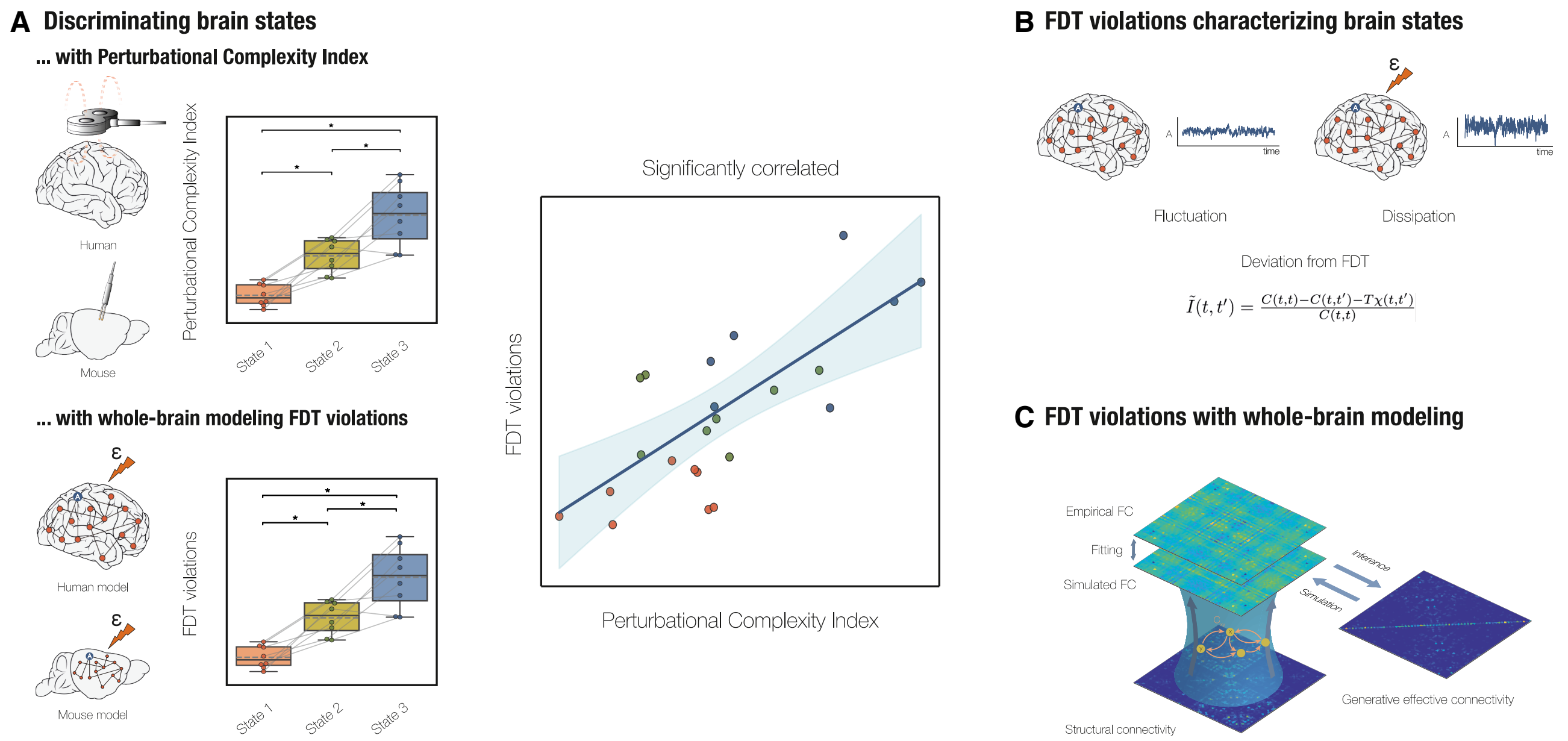

Figure 1. Framework for computing PCI and FDT violations from evoked and spontaneous brain dynamics

(A) PCI is computed from TMS-evoked or intracortical responses by binarizing spatiotemporal patterns of cortical source activations and quantifying their algorithmic complexity using Lempel-Ziv compression. PCI has proven to be a reliable and robust indicator, consistently distinguishing between conscious and unconscious conditions with high accuracy.[10,15] FDT violations, estimated from spontaneous brain dynamics using whole-brain modeling, also discriminate between brain states and correlate strongly with PCI. This supports a robust empirical link between PCI and non-equilibrium spontaneous dynamics, while also introducing FDT violations as a complementary, stimulation-free measure associated with conscious state.

(B) Here, we use a model-based formalism for FDT violations that captures the mismatch between the model-derived response to a small theoretical perturbation and the response that would be expected if the system obeyed equilibrium. In equilibrium systems, fluctuations fully determine the response function; in the brain, departures from this relationship indicate non-equilibrium dynamics. FDT violations have been used in previous studies to characterize brain states.[31,32]

(C) Whole-brain modeling is applied at the individual level to reproduce spontaneous brain dynamics and capture the underlying asymmetrical structure of connectivity, referred to as generative effective connectivity.

PCI was then computed using the Lempel-Ziv compression algorithm. In parallel, we quantified FDT violations using a model-based formalism that captures the mismatch between the effective response to a small theoretical perturbation and the response that would be expected if the system obeyed equilibrium (Figure 1B). In equilibrium systems, fluctuations fully determine the response function; in the brain, departures from this relationship indicate non-equilibrium dynamics.

To obtain the effective response function, we used whole-brain models. First, the directed asymmetric interactions between brain regions were estimated by fitting a multivariate Ornstein-Uhlenbeck process to the empirical data. This approach has been successfully applied to characterize large-scale brain dynamics in both electroencephalography (EEG) and fMRI studies and has proven effective in extracting biologically meaningful structure from spontaneous activity.[36–38] The model fitting was performed using a pseudo-gradient descent algorithm that simultaneously captures the zero-lag functional correlation matrix and the time-lagged covariance matrix, the latter encoding directional temporal interactions (Figure 1C). Once the model was fitted, FDT violations were computed using the off-equilibrium extension provided in previous studies.[32,39,40] After both PCI and FDT violations were estimated, we assessed their correlation across conditions, as well as their relationship with clinical scores (Figure 1A).

We applied this pipeline across three distinct datasets: (1) invasive local field potential (LFP) recordings from mice under graded anesthesia, (2) scalp EEG from neurotypical human participants in wakefulness and under three anesthetic agents, and (3) scalp EEG from patients with DoC. Within each dataset, we found that FDT violations increased from unconscious to conscious states and tightly co-varied with PCI, supporting a robust association between spontaneous non-equilibrium signatures and perturbational measures. These findings provide further support for a link between non-equilibrium brain dynamics and perturbation-based indices while introducing FDT violations as a non-invasive measure associated with conscious state.

FDT violations track levels of anesthesia in animal models

To investigate the relationship between PCI and FDT violations and to evaluate whether they can effectively track graded anesthesia, we first analyzed electrophysiological data from animal models. We examined large-scale LFP recordings from eight mice under three distinct levels of isoflurane anesthesia, for which both spontaneous and evoked brain activity were recorded.

As illustrated in Figure S1A, spontaneous activity during anesthesia was characterized by slow oscillations,[41] reflecting the

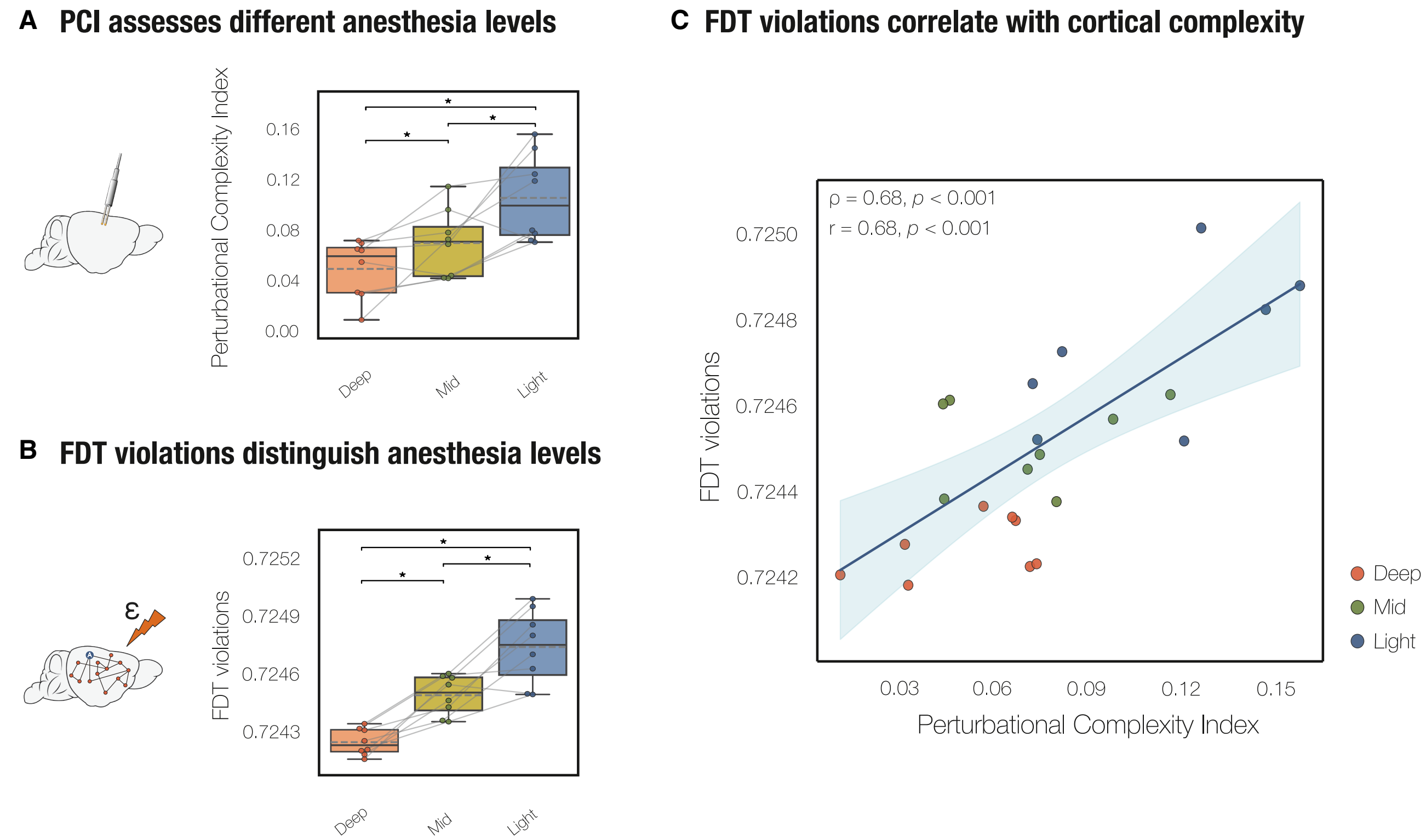

Figure 2. FDT violations track graded levels of anesthesia in mice and correlate with the PCI

(A) PCI, calculated from multi-unit activity-based evoked responses, significantly discriminated between all anesthesia conditions. PCI consistently tracked the anesthesia levels in mice (Spearman's $\rho = 0.71$, $p < 0.001$).

(B) FDT violations calculated from the whole-brain model fitted to empirical spontaneous activity were also able to discriminate among all the conditions, as reflected by a strong monotonic relationship (Spearman's $\rho = 0.885$, $p < 0.001$).

(C) FDT violations correlate with the PCI (Spearman's $\rho = 0.68$, $p < 0.001$; Pearson's $r = 0.68$, $p < 0.001$). A linear mixed-effects model, controlling for anesthesia level and incorporating a random intercept for subject, confirmed that the association remained significant ($\beta = 0.613 \pm 0.298$ s.e., $p = 0.037$). Leave-one-subject-out cross-validation confirmed out-of-sample generalization (Spearman's $\rho = 0.553$, $p = 0.0051$; Pearson's $r = 0.615$, $p = 0.0014$). The line indicates the linear regression fit, and the shaded area indicates the corresponding 95% confidence band. Thin gray lines connect measurements from the same mouse across conditions. All the pairwise comparisons were performed using Wilcoxon signed-rank tests and post hoc corrected with Holm-Bonferroni. *$p < 0.05$.

coordinated dynamics of large populations of neurons transitioning between active periods, known as Up states, and silent periods, referred to as Down states.[42,43] The depth of anesthesia was associated with a slower dominant frequency and a higher occurrence of silent periods (See Notes S1 and S2).

PCI, derived from multi-unit activity-based evoked responses, significantly discriminated all anesthesia conditions and consistently tracked anesthesia depth (Spearman's $\rho = 0.71$, $p < 0.001$; Figure 2A).

In parallel, FDT violations, estimated from whole-brain models fitted to empirical spontaneous activity, also significantly distinguished all three anesthesia levels (Figure 2B). This was supported by a strong monotonic relationship with anesthesia depth (Spearman's $\rho = 0.885$, $p < 0.001$). FDT violations were also highly correlated with the dominant frequency of the slow oscillations (see Note S2; Figure S2).

Figure 2C shows a strong correlation between PCI and FDT violations (Spearman's $\rho = 0.68$, $p < 0.001$; Pearson's $r = 0.68$, $p < 0.001$). A linear mixed-effects model, controlling for anesthesia level and including a random intercept for subject, confirmed that the association remained significant ($\beta = 0.613 \pm 0.298$ s.e., $p = 0.037$). Importantly, the relationship between PCI and FDT violations remained significant under leave-one-subject-out cross-validation, demonstrating out-of-sample generalization across subjects (Spearman's $\rho = 0.553$, $p = 0.0051$; Pearson's $r = 0.615$, $p = 0.0014$).

FDT violations distinguish wakefulness from anesthesia in human EEG

To generalize the findings to humans, we analyzed EEG recordings from neurotypical individuals. The dataset included fifteen healthy participants recorded during wakefulness under eyes-open or eyes-closed conditions. Participants were randomly assigned to one of three anesthetic conditions ($N = 5$ each for xenon, propofol, and ketamine), resulting in a total of 30 recordings. Participants under ketamine anesthesia reported vivid, dream-like experiences during unresponsiveness, consistent with previous studies describing ketamine-induced conscious states.[6,44,45] Accordingly, the grouping by conscious state is not independent of anesthetic condition and is interpreted here descriptively.

Consistent with these state differences, spontaneous activity during anesthesia was dominated by slow oscillations, whereas

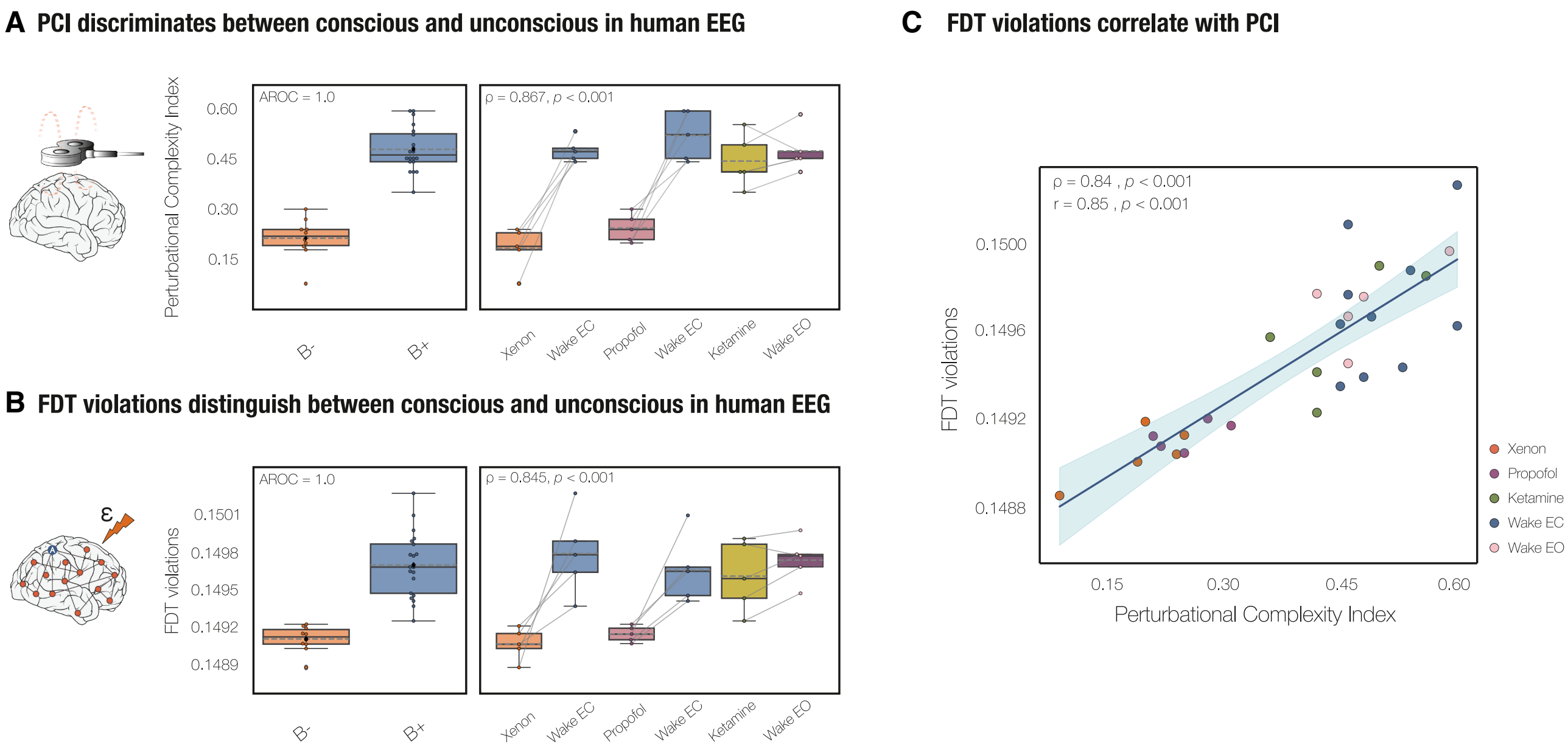

Figure 3. FDT violations in human EEG track level of consciousness during wakefulness and anesthesia and correlate with PCI

(A) PCI discriminated between participants who retrospectively reported some form of conscious experience (wakefulness and ketamine; B+) and those who did not (xenon and propofol; B-). PCI showed perfect group separation in this sample (AROC = 1.0). A strong monotonic relationship with condition was observed (Spearman's $\rho = 0.867$, $p < 0.001$).

(B) FDT violations, computed from whole-brain models fitted to spontaneous EEG activity, also differentiated participants who reported conscious experience from those who did not, AROC = 1.0, indicating perfect group separation in this sample. A monotonic relationship was observed (Spearman's $\rho = 0.845$, $p < 0.001$), consistent with sensitivity to differences in conscious state.

(C) FDT violations were strongly correlated with PCI (Spearman's $\rho = 0.84$, $p < 0.001$; Pearson's $r = 0.85$, $p < 0.001$). A linear mixed-effects model, controlling for anesthesia state and drug type while including subject identity as a random intercept, confirmed that the association between FDT violations and PCI remained significant ($\beta = 0.564 \pm 0.159$ s.e., $p < 0.001$). Leave-one-subject-out cross-validation showed robust generalization to unseen subjects (Spearman's $\rho = 0.803$, $p < 0.001$; Pearson's $r = 0.831$, $p < 0.001$). The line indicates the linear regression fit, and the shaded area indicates the corresponding 95% confidence band. Thin gray lines connect measurements from the same participant across conditions.

wakefulness was characterized by prominent alpha rhythms (Figure S1B).[22,46]

In this context, PCI remained high in ketamine and did not distinguish ketamine from wakefulness, while both wakefulness and ketamine were clearly separated from xenon and propofol, consistent with preserved conscious content during ketamine anesthesia despite behavioral unresponsiveness. Group separability, quantified using the area under the receiver operating characteristic (AROC), was 1.0, indicating perfect separation between report-based conscious and unconscious states in this sample. A strong monotonic relationship was also observed between PCI and conditions (Spearman's $\rho = 0.867$; $p < 0.001$), indicating that PCI could track consciousness during anesthesia conditions (Figure 3A).

FDT violations showed the same qualitative pattern: values remained elevated in ketamine and did not separate ketamine from wakefulness, while both wakefulness and ketamine were distinguished from xenon and propofol. This was supported by an AROC of 1.0 and a similarly strong monotonic correlation (Spearman's $\rho = 0.845$, $p < 0.001$; Figure 3B).

FDT violations were strongly correlated with PCI (Spearman's $\rho = 0.84$, $p < 0.001$; Pearson's $r = 0.85$, $p < 0.001$; Figure 3C). A linear mixed-effects model, controlling for anesthesia state (anesthetized vs. awake) and drug type, while including subject identity as a random intercept, confirmed that the association between FDT violations and PCI remained significant ($\beta = 0.564 \pm 0.159$ s.e., $p < 0.001$).

The association also generalized robustly to unseen subjects under leave-one-subject-out cross-validation. Across held-out folds, predicted and observed FDT violations remained strongly associated both in rank order (Spearman's $\rho = 0.803$, $p < 0.001$) and in linear terms (Pearson's $r = 0.831$, $p < 0.001$).

FDT violations correlate with PCI and clinical scores in disorders of consciousness

Lastly, to evaluate the association in a clinically relevant cohort, we analyzed EEG data from patients diagnosed with chronic DoC, including individuals classified as UWS, MCS−, and MCS+. Clinical diagnosis was based on repeated CRS-R assessments conducted at least three times within one week. EEG recordings were obtained from 17 patients, including individuals with vascular ($N = 13$) and traumatic ($N = 4$) etiologies. Importantly, analyses of FDT violations were performed blind to PCI values, diagnostic category, CRS-R scores, and other clinical information, which were incorporated only afterward for evaluation.

Consistent with previous reports, PCI reliably distinguished between behaviorally responsive and unresponsive patients

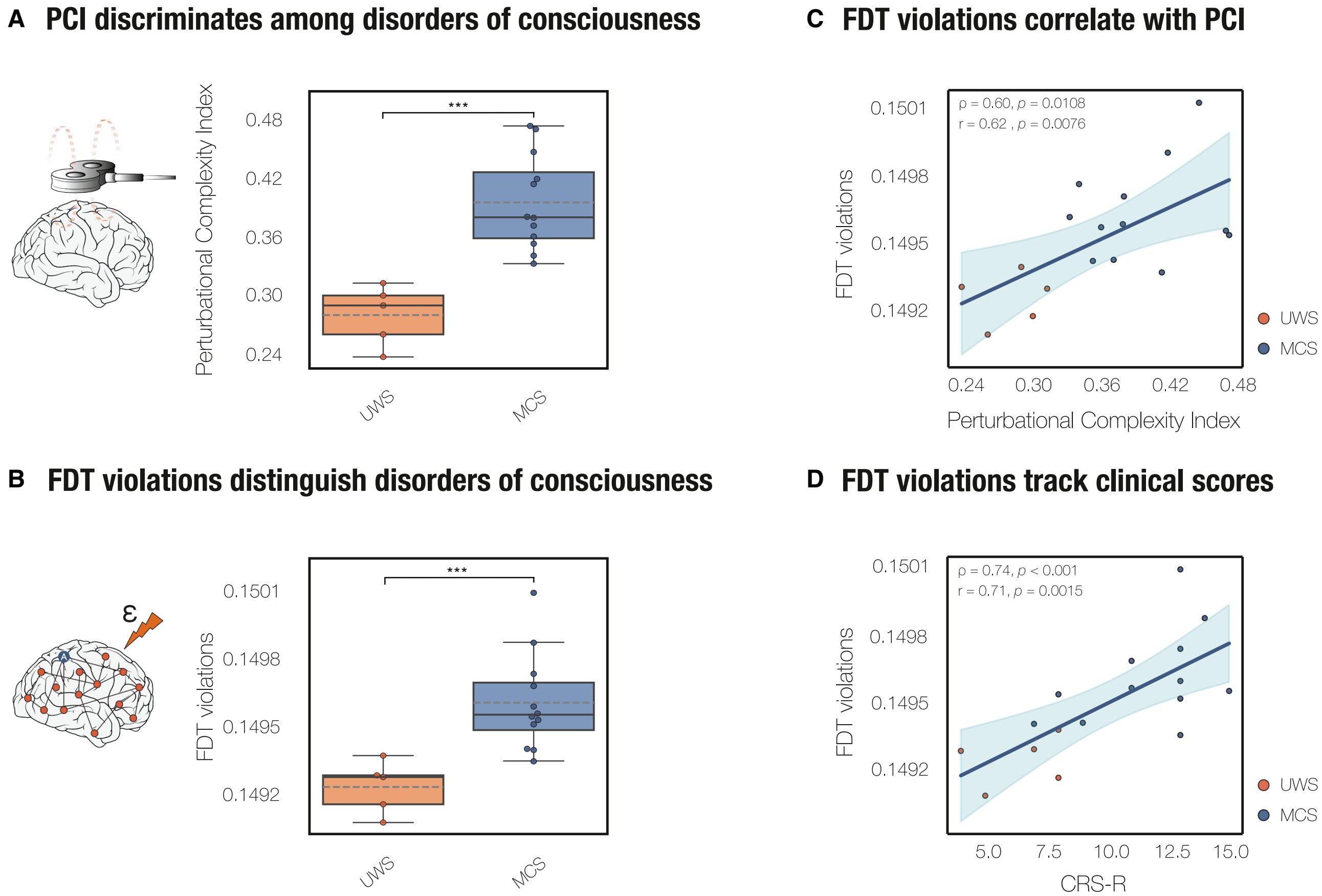

Figure 4. FDT violations distinguish behaviorally responsive from behaviorally unresponsive patients with disorders of consciousness and correlate with PCI and clinical scores

(A) PCI discriminated between behaviorally responsive (MCS−/MCS+) and behaviorally unresponsive patients (UWS) with high accuracy (Cliff's $\delta = 1.0$, $p < 0.001$) and showed perfect group separation in this sample (AROC = 1.0).

(B) FDT violations also distinguished conscious from unconscious patients with similarly high accuracy (Cliff's $\delta = 0.967$, $p < 0.001$) and achieved an AROC of 0.983.

(C) FDT violations were significantly correlated with PCI values, indicating that both metrics capture related aspects of consciousness in a clinical context (Spearman's $\rho = 0.60$, $p = 0.0108$; Pearson's $r = 0.62$, $p = 0.0076$). An ANCOVA including the *FDT violations* × *Condition* interaction term revealed no evidence of slope heterogeneity between groups, indicating that the relationship was conserved across conditions ($F(1,13) = 0.007$, $p = 0.934$; Freedman-Lane permutation $p_{perm} = 0.908$). Leave-one-subject-out cross-validation showed moderate out-of-sample generalization, with a significant rank-based association (Spearman's $\rho = 0.539$, $p = 0.0255$) and a weaker linear association at trend level (Pearson's $r = 0.464$, $p = 0.060$).

(D) FDT violations were also strongly correlated with clinical scales such as CRS-R scores (Spearman's $\rho = 0.736$, $p < 0.001$; Pearson's $r = 0.707$, $p = 0.0015$). Under leave-one-subject-out cross-validation, this association remained significant out of sample (Spearman's $\rho = 0.598$, $p = 0.0112$; Pearson's $r = 0.606$, $p = 0.009$). In C and D, the lines indicate the linear regression fits, and the shaded areas indicate the corresponding 95% confidence bands. Group comparisons in A and B were performed using two-sided Mann-Whitney U tests. ***$p < 0.001$.

(Cliff's $\delta = 1.0$, $p < 0.001$; Figure 4A). Discriminative performance, quantified using AROC, was 1.0, indicating perfect separation between the two groups in this sample.

FDT violations also significantly differentiated behaviorally responsive from unresponsive patients in this cohort (Cliff's $\delta = 0.967$, $p < 0.001$; Figure 4B). The corresponding AROC was 0.983, indicating strong discriminative performance in this clinical setting.

In this more challenging clinical context, FDT violations remained significantly correlated with PCI (Spearman's $\rho = 0.60$, $p = 0.0108$; Pearson's $r = 0.62$, $p = 0.0076$; Figure 4C). An ANCOVA including an *FDT violations* × *Condition* interaction term revealed no evidence of slope heterogeneity between groups, indicating that the relationship was conserved across conditions ($F(1,13) = 0.007$, $p = 0.934$; Freedman-Lane permutation $p_{perm} = 0.908$). In the DoC cohort, leave-one-subject-out cross-validation showed moderate out-of-sample generalization. Predicted and observed FDT violations remained significantly associated at the rank level (Spearman's $\rho = 0.539$, $p = 0.0255$), whereas the linear association was weaker and reached trend level (Pearson's $r = 0.464$, $p = 0.060$). FDT violations were significantly correlated with CRS-R scores (Spearman's $\rho = 0.736$, $p < 0.001$; Pearson's $r = 0.707$, $p = 0.0015$; Figure 4D), as was PCI (Spearman's $\rho = 0.57$, $p = 0.0116$; Pearson's $r = 0.56$, $p = 0.018$). Under leave-one-subject-out cross-validation, the association between FDT violations and

CRS-R remained significant out of sample (Spearman's ρ = 0.598, p = 0.0112; Pearson's r = 0.606, p = 0.009), whereas the corresponding association for PCI was weaker and did not reach significance (Spearman's ρ = 0.414, p = 0.098; Pearson's r = 0.411, p = 0.101).

We additionally examined a small post-anoxic subgroup in a supplementary analysis (N = 9; Note S3; Figure S3). In this subgroup, PCI showed a marked floor effect, with PCI values at floor in all patients with UWS. Spontaneous EEG activity was frequently low-voltage, limiting the interpretability of graded PCI-based relationships and making estimation of model-derived non-equilibrium structure more challenging. FDT violations remained estimable and retained variability, but separation between diagnostic categories was modest and did not reach statistical significance.

DISCUSSION

Here, we show that FDT violations are robustly associated with PCI across multiple electrophysiological datasets in mice and humans. Across species, modalities, and experimental and clinical conditions, FDT violations varied systematically across brain states and correlated strongly with PCI. Together, these results suggest a common framework relating spontaneous dynamics, perturbational complexity, and model-based measures of non-equilibrium dynamics.

Currently, the assessment of consciousness relies on behavioral responsiveness, most commonly evaluated with the CRS-R scale in the clinical context. However, an absence of responsiveness cannot be taken as evidence of absence of consciousness.[3–5] Motor, sensory, or executive impairments may prevent behavioral interaction with the environment, even when awareness is preserved, as in healthy subjects during dreaming, under general anesthesia, or in cases of severe brain injury.[7–9,47] This limitation has motivated the search for non-behavioral markers of consciousness. Early candidates such as the P3b component, global EEG activation, and cortical oscillations showed initial promise but have proven unreliable.[48–50] In practice, they failed to consistently separate minimally conscious from unresponsive patients or to index consciousness properly across altered states.[34,51–55]

The development of new quantitative complexity indices marked an important advance, inspired by theoretical neuroscience and particularly by integrated information theory.[13,56–58] Empirically, brain complexity has been estimated either through analysis of spontaneous activity[59–62] or by quantifying reactivity patterns to cortical perturbations.[6,10] Among these, PCI has emerged as a robust and generalizable marker, reliably distinguishing conscious from unconscious conditions, as also seen here in Figures 2A, 3A, and 4A.[10,11,15] While PCI can be traced back to theoretical principles,[16–22] its relationship to broader principles of physics remains unclear.

Recent work has begun to address this question by linking perturbational complexity to critical dynamics[63] and, more recently, by proposing modeling links between model-based perturbational complexity and irreversibility.[33] Here, by contrast, we provide, to our knowledge, the first direct empirical evidence that PCI derived from perturbational recordings covaries with FDT violations and non-equilibrium brain dynamics. Across species, brain states, and recording modalities, states with higher PCI values, reflecting richer and more differentiated evoked responses, were associated with stronger FDT violations, whereas states with lower PCI values corresponded to more equilibrium-like spontaneous dynamics (Figures 2C, 3C, and 4C). Leave-one-subject-out cross-validation further supported the robustness of this relationship. Generalization was strong in mice and human anesthesia, and more modest in DoC, where the association remained significant at the rank level but weaker in strictly linear terms. This pattern is consistent with the smaller sample size and greater clinical heterogeneity of that cohort. Across datasets, the results indicate that the observed association is not driven solely by subject-specific fits. In addition, in the DoC cohort, analyses of FDT violations were carried out without access to PCI values, diagnosis, CRS-R scores, or other subject-level clinical outcomes, reducing the risk that the reported associations arose from circular analysis choices or implicit optimization toward target measures. These findings support a connection between brain complexity and non-equilibrium brain dynamics, and help place previous empirical and theoretical results within a broader non-equilibrium framework.[6,10,15,16,35] From this perspective, the brain's capacity to generate complex, integrated cortical responses in conscious states may be associated with a breakdown in the relationship between spontaneous fluctuations and evoked activity. Taken together, PCI and FDT violations may capture complementary aspects of conscious brain states: their effective departure from equilibrium. Further support for this broader non-equilibrium interpretation comes from complementary analyses of other non-equilibrium measures, including entropy production, temporal irreversibility, and coupling asymmetry, which showed parallel reductions with loss of consciousness (see Note S4; Figures S4, S5, and S6).

Our study further suggests that FDT violations may constitute a promising measure associated with conscious state, applicable to electrophysiological recordings in both humans and animal models. FDT violations successfully distinguished three levels of graded anesthesia in mouse LFP data (Figure 2B). In humans, FDT violations reliably differentiated wakefulness from xenon- and propofol-induced unresponsiveness (Figure 3B). However, similar to PCI, they did not distinguish between wakefulness and the dissociative state induced by ketamine at anesthetic concentrations.[6] This observation aligns with prior clinical studies and with participants reporting "ketamine dreams," rich inner experiences upon awakening, often unrelated to their surgical environment.[6,44,45] Since ketamine, xenon, and propofol anesthetic protocols all induced behavioral unresponsiveness, the preservation of relatively high PCI and FDT violations under ketamine argues against a purely responsiveness-based interpretation and against a simple wake-versus-anesthetized dichotomy.

In patients with DoC, FDT violations significantly differentiated patients in UWS from those in MCS−/MCS+ (Figure 4B) and correlated with CRS-R scores (Figure 4D). Notably, in the present DoC cohort, the association between FDT violations and CRS-R scores was stronger than the corresponding association for PCI and remained significant under leave-one-subject-out

cross-validation. This difference should nevertheless be interpreted cautiously given the modest sample size and clinical heterogeneity of the dataset. These findings support the potential clinical relevance of FDT violations in DoC, while also indicating that in clinical settings they may remain sensitive to dimensions that covary with behavioral responsiveness. Overall, FDT violations captured meaningful distinctions in brain states across all datasets and at multiple scales, introducing a model-based approach to study brain dynamics across levels of consciousness.

In the present study, we also examined a small subgroup of severe post-anoxic patients as a clinically relevant boundary condition. In this subgroup, PCI values were at floor in all patients with UWS, while spontaneous EEG activity was often low-voltage, making graded relationships with PCI difficult to assess and the estimation of non-equilibrium brain dynamics more challenging. Even under these conditions, FDT violations could still be estimated and retained variability within the subgroup. However, separation between diagnostic categories was modest and did not reach statistical significance, consistent with the limited sample size and the physiologically constrained nature of this subgroup. Severe post-anoxic injury should therefore be interpreted as an important boundary condition of the framework, in which both perturbational and spontaneous measures may be strongly constrained by diffuse cortical damage. Therefore, the main conclusions of the present study apply to conditions in which spontaneous and perturbational brain dynamics remain sufficiently preserved to support meaningful estimation of PCI and FDT violations. Future work should test this framework in larger post-anoxic cohorts and assess whether refined modeling improves the estimation of directional asymmetries and FDT violations in patients with globally reduced cortical activity and low-voltage EEG patterns.[64–67]

This framework may also be used to capture focal alterations in brain dynamics. Following brain lesions, for example, quantifying local FDT violations could yield spatially resolved maps of non-equilibrium dynamics, offering insight into how injury reshapes integration and pointing to potential targets for restoring wake-like activity through neuromodulation.[68] Taken together, this perspective suggests that FDT violations may offer a non-invasive approach for characterizing alterations in brain dynamics across a wide range of neurological conditions, although validation remains necessary.

Moreover, importantly, the relationship between FDT violations and fundamental neural dynamics remains an open area of investigation. In the mouse dataset, FDT violations were correlated with the dominant frequency of the slow oscillations observed under anesthesia, supporting a link between neural dynamics and non-equilibrium measures (see Note S2; Figure S2). Indeed, the emergence of spontaneous slow oscillations, a hallmark of low-complexity brain states, is commonly observed during non-rapid eye movement sleep, in patients with UWS, even during periods of eyes open, and in other pathological states.[46,54,59,69] These oscillations reflect cortical bistability as they are characterized by alternating phases of neuronal tonic firing and neuronal silence.[70] Experimental and computational studies across humans, rodents, and cortical slice preparations converge in identifying cortical bistability and OFF-periods as key disruptors of large-scale network integration and complexity.[16–19,22] Cortical perturbations reveal that bistability causally constrains network dynamics.[17–19,22] PCI reflects this directly, increasing with rich, specific interactions and decreasing when OFF-periods yield restricted, stereotypical responses.[10] Theoretically, FDT violations provide a natural framework to study this phenomenon. They quantify the breakdown in the relationship between spontaneous fluctuations and evoked activity, thereby providing a link between non-equilibrium brain dynamics and perturbational complexity.

It is also worth noting that while here we implemented a model-based whole-brain framework, similar approaches could be directly applied to empirical brain dynamics under linear stochastic assumptions.[71] Importantly, the model-based perspective remains advantageous as it allows for in silico manipulations, offering a path to bridge empirical data with theoretical models.

All in all, this study identifies FDT violations as a complementary, non-invasive, model-based measure associated with PCI and conscious state across species, recording modalities, and scales. Most importantly, by establishing a systematic relationship between estimates of FDT violations and PCI across brain states and conditions, we show that perturbational complexity and non-equilibrium dynamics are closely related. Together, these results support a common framework linking spontaneous brain dynamics, perturbational complexity, and model-based non-equilibrium measures, offering a complementary thermodynamic perspective on consciousness. These findings open new avenues for understanding the nature of consciousness and motivate further work toward developing tools to assess its loss and recovery in health and disease.

Limitations of the study

It should be noted that this study has several limitations. First, electrophysiological measures can be affected by artifacts or limited electrode coverage, although standard preprocessing minimizes such effects. In patients with DoC, diagnostic reliability is further challenged by behavioral fluctuations and heterogeneous signal quality; while the CRS-R vigilance protocol mitigated this, future work should examine robustness in larger and more diverse cohorts.[72,73] Time since injury may also influence EEG features, but prior studies suggest only marginal effects, and our focus was on diagnostic capacity rather than longitudinal progression.[74] The modest sample size, especially in the clinical cohort, also limits the precision of the reported statistics. Methodologically, our framework relies on a linear stochastic approximation for analytical tractability, which may underestimate nonlinear aspects of brain activity, and assumes spatially homogeneous noise covariance across regions. Future work should assess whether allowing heterogeneous noise covariance improves the model and alters the estimation of FDT violations. FDT violation values should also be interpreted cautiously across species or recording modalities, given differences in spatial resolution, signal properties, and recording conditions. Moreover, although most short, detrended epochs were compatible with local weak stationarity, small residual non-stationarity, metastability, or hidden state transitions may still contribute to the estimated FDT violations. Accordingly, the quantities reported here should be interpreted as model-based

measures of effective non-equilibrium in the observed signals, rather than as direct measurements of physical thermodynamic dissipation. Despite these caveats, the consistency of results across species, brain states, and modalities underscores the robustness of the main findings, but further validation in larger, independent, and more varied populations will be essential.

RESOURCE AVAILABILITY

Lead contact

Requests for further information and resources should be directed to and will be fulfilled by the lead contact, Tomas Berjaga-Buisan (tomas.berjaga@upf.edu).

Materials availability

This study did not generate new unique reagents or materials.

Data and code availability

- Raw LFP recordings from eight adult male C57BL/6J mice across different anesthesia levels are publicly available at the EBRAINS repository (https://doi.org/10.25493/WKA8-Q4T).
- The human anesthesia EEG dataset analyzed in this study was not generated specifically for the present study. It was originally collected in the context of the EU Horizon 2020 LUMINOUS project (grant no. 686764) and deposited by the original investigators on Zenodo (https://doi.org/10.5281/zenodo.806176), where it is indexed in the EU Open Research Repository. The repository record and metadata are publicly accessible, while the underlying human participant-level EEG data are available under restricted access due to ethical restrictions associated with the original data collection. Access requests may be submitted through Zenodo and are assessed by the relevant data custodians.
- All original code used for data analysis, modeling of spontaneous fluctuations, fitting of generative effective connectivity, computation of FDT violations, and calculation of complementary thermodynamic metrics has been deposited at Zenodo and is publicly available at https://doi.org/10.5281/zenodo.20719663. The code is also available in the associated GitHub repository: https://github.com/tomasberjagabuisan/Thermo-Consciousness
- Additional data, including raw EEG signals from patients with DoC, along with associated metadata, are available from the authors upon reasonable request and subject to a data sharing agreement.

ACKNOWLEDGMENTS

We are grateful to all the patients and volunteers who participated in the experiments and made this work possible. We thank the members of the various laboratories involved in data acquisition for their invaluable contributions. We would like to thank Irene Acero and Paula García for their insightful comments on the draft. We also want to thank Inés Blázquez for assisting with the figures. J.M.M. acknowledges financial support from CONICET through an external scholarship for researchers. M.L.K. is supported by the Centre for Eudaimonia and Human Flourishing (funded by the Pettit and Carlsberg Foundations) and Center for Music in the Brain (funded by the Danish National Research Foundation, grant no. DNRF117). M. Corbetta is supported by Fondazione Cassa di Risparmio di Padova e Rovigo (CARIPARO) (GA 55403), the Italian Ministry of Health (NEUROCONN RF-2008-12366899, EYEMOVINSTROKE RF-2019-12369300), and the EU H2020 project, H2020-SC5–2019-2, (Grant Agreement 869505). M.V.S.V. is supported by PID2023-152918OB-I00 funded by MICIU/AEI/10.13039/501100011033/FEDER, UE and by AGAUR 2021-SGR-01165. M. Corbetta, M.V.S.V., M.M., Y.S.P., and G.D. are supported by the project NEurological MEchanismS of Injury, and Sleep-like cellular dynamics (NEMESIS; ref. 101071900) funded by the EU ERC Synergy Horizon Europe. Y.S.P. also acknowledges support from grant PID2024-162576NA-I00 funded by MICIU/AEI/10.13039/501100011033 and by ERDF A way of making Europe. G.D. also acknowledges support from grant PID2022-136216NB-I00 funded by MICIU/AEI/10.13039/501100011033; by ERDF A way of making Europe, ERDF, EU; and AGAUR research support grant (2021 SGR 00917) funded by the Department of Research and Universities of the Generalitat of Catalunya.

AUTHOR CONTRIBUTIONS

T.B.B., J.M.M., Y.S.P., and G.D. conceptualized and designed the study; T.B.B. and J.M.M. performed the formal analysis and developed the modeling framework; T.B.B., J.M.M., M. Cortada, M.A.C., M. Corbetta, M.V.S.V., M.M., Y.S.P., and G.D. contributed to the methodology; J.M.M., M. Cortada, and S.M.G. performed complementary analyses; M. Cortada, M.A.C., G.G., S.S., M.V.S.V., and M.M. provided resources; T.B.B. performed additional data curation and filtering; T.B.B. generated all visualizations with input from M.L.K. G.D. supervised the research; T.B.B. wrote the original manuscript. All authors reviewed and edited the manuscript.

DECLARATION OF INTERESTS

M.M. is a co-founder and shareholder of Intrinsic Powers, a spin-off of the University of Milan. S.S. is an advisor of Intrinsic Powers. M.M., S.S., and M.A.C. are named on a patent application related to consciousness assessment. These relationships and the patent application in no way affected the content of this article. The remaining authors declare no competing interests.

DECLARATION OF GENERATIVE AI AND AI-ASSISTED TECHNOLOGIES IN THE WRITING PROCESS

During the preparation of this work, the authors used ChatGPT (OpenAI; GPT-5.4) only to assist with existing text revision, including language editing and wording changes. It was not used to write original manuscript content or to generate any scientific content, analyses, interpretations, or conclusions. All scientific content was written by the authors, who reviewed and edited the revised text as needed and take full responsibility for the published article.

STAR★METHODS

Detailed methods are provided in the online version of this paper and include the following:

- KEY RESOURCES TABLE
- EXPERIMENTAL MODEL AND STUDY PARTICIPANT DETAILS
  - Mouse electrophysiological data
  - Human EEG data during wakefulness and anesthesia
  - Human EEG data in disorders of consciousness
  - Signal preprocessing
- METHOD DETAILS
  - Perturbational Complexity Index
  - Whole-brain modeling
  - Model optimization
  - FDT violations
- QUANTIFICATION AND STATISTICAL ANALYSIS
  - Statistical analysis

SUPPLEMENTAL INFORMATION

Supplemental information can be found online at https://doi.org/10.1016/j.celrep.2026.117782.

Received: December 3, 2025
Revised: April 21, 2026
Accepted: July 15, 2026
Published: July 31, 2026

REFERENCES

1. Giacino, J.T., Schnakers, C., Rodriguez-Moreno, D., Kalmar, K., Schiff, N., and Hirsch, J. (2009). Behavioral assessment in patients with disorders of consciousness: gold standard or fool's gold? Prog. Brain Res. *177*, 33–48. https://doi.org/10.1016/s0079-6123(09)17704-x.
2. Kalmar, K., and Giacino, J.T. (2005). The JFK coma recovery scale—revised. Neuropsychol. Rehabil. *15*, 454–460. https://doi.org/10.1080/09602010443000425.
3. Alkire, M.T., Hudetz, A.G., and Tononi, G. (2008). Consciousness and Anesthesia. Science *322*, 876–880. https://doi.org/10.1126/science.1149213.
4. Laureys, S., Owen, A.M., and Schiff, N.D. (2004). Brain function in coma, vegetative state, and related disorders. Lancet Neurol. *3*, 537–546. https://doi.org/10.1016/s1474-4422(04)00852-x.
5. Sanders, R.D., Tononi, G., Laureys, S., Sleigh, J.W., and Warner, D.S. (2012). Unresponsiveness $\neq$ Unconsciousness. Anesthesiology *116*, 946–959. https://doi.org/10.1097/aln.0b013e318249d0a7.
6. Sarasso, S., Boly, M., Napolitani, M., Gosseries, O., Charland-Verville, V., Casarotto, S., Rosanova, M., Casali, A.G., Brichant, J.-F., Boveroux, P., et al. (2015). Consciousness and Complexity during Unresponsiveness Induced by Propofol, Xenon, and Ketamine. Curr. Biol. *25*, 3099–3105. https://doi.org/10.1016/j.cub.2015.10.014.
7. Sebel, P.S., Bowdle, T.A., Ghoneim, M.M., Rampil, I.J., Padilla, R.E., Gan, T.J., and Domino, K.B. (2004). The Incidence of Awareness During Anesthesia: A Multicenter United States Study. Anesth. Analg. *99*, 833–839. https://doi.org/10.1213/01.ane.0000130261.90896.6c.
8. Laureys, S., Celesia, G.G., Cohadon, F., Lavrijsen, J., León-Carrión, J., Sannita, W.G., Sazbon, L., Schmutzhard, E., Von Wild, K.R., Zeman, A., et al. (2010). Unresponsive wakefulness syndrome: a new name for the vegetative state or apallic syndrome. BMC Med. *8*, 68. https://doi.org/10.1186/1741-7015-8-68.
9. Fernández-Espejo, D., and Owen, A.M. (2013). Detecting awareness after severe brain injury. Nat. Rev. Neurosci. *14*, 801–809. https://doi.org/10.1038/nrn3608.
10. Casali, A.G., Gosseries, O., Rosanova, M., Boly, M., Sarasso, S., Casali, K.R., Casarotto, S., Bruno, M.-A., Laureys, S., Tononi, G., and Massimini, M. (2013). A Theoretically Based Index of Consciousness Independent of Sensory Processing and Behavior. Sci. Transl. Med. *5*, 198ra105. https://doi.org/10.1126/scitranslmed.3006294.
11. Comolatti, R., Pigorini, A., Casarotto, S., Fecchio, M., Faria, G., Sarasso, S., Rosanova, M., Gosseries, O., Boly, M., Bodart, O., et al. (2019). A fast and general method to empirically estimate the complexity of brain responses to transcranial and intracranial stimulations. Brain Stimul. *12*, 1280–1289. https://doi.org/10.1016/j.brs.2019.05.013.
12. Tononi, G., and Edelman, G.M. (1998). Consciousness and Complexity. Science *282*, 1846–1851. https://doi.org/10.1126/science.282.5395.1846.
13. Deco, G., Tononi, G., Boly, M., and Kringelbach, M.L. (2015). Rethinking segregation and integration: contributions of whole-brain modelling. Nat. Rev. Neurosci. *16*, 430–439. https://doi.org/10.1038/nrn3963.
14. Sarasso, S., Casali, A.G., Casarotto, S., Rosanova, M., Sinigaglia, C., and Massimini, M. (2021). Consciousness and complexity: a consilience of evidence. Neurosci. Conscious. *2021*, niab023. https://doi.org/10.1093/nc/niab023.
15. Casarotto, S., Comanducci, A., Rosanova, M., Sarasso, S., Fecchio, M., Napolitani, M., Pigorini, A., G Casali, A., Trimarchi, P.D., Boly, M., et al. (2016). Stratification of unresponsive patients by an independently validated index of brain complexity. Ann. Neurol. *80*, 718–729. https://doi.org/10.1002/ana.24779.
16. D'Andola, M., Rebollo, B., Casali, A.G., Weinert, J.F., Pigorini, A., Villa, R., Massimini, M., and Sanchez-Vives, M.V. (2018). Bistability, Causality, and Complexity in Cortical Networks: An In Vitro Perturbational Study. Cereb. Cortex *28*, 2233–2242. https://doi.org/10.1093/cercor/bhx122.
17. Cavelli, M.L., Mao, R., Findlay, G., Driessen, K., Bugnon, T., Tononi, G., and Cirelli, C. (2023). Sleep/wake changes in perturbational complexity in rats and mice. iScience *26*, 106186. https://doi.org/10.1016/j.isci.2023.106186.
18. Pigorini, A., Sarasso, S., Proserpio, P., Szymanski, C., Arnulfo, G., Casarotto, S., Fecchio, M., Rosanova, M., Mariotti, M., Lo Russo, G., et al. (2015). Bistability breaks-off deterministic responses to intracortical stimulation during non-REM sleep. Neuroimage *112*, 105–113. https://doi.org/10.1016/j.neuroimage.2015.02.056.
19. Rosanova, M., Fecchio, M., Casarotto, S., Sarasso, S., Casali, A.G., Pigorini, A., Comanducci, A., Seregni, F., Devalle, G., Citerio, G., et al. (2018). Sleep-like cortical OFF-periods disrupt causality and complexity in the brain of unresponsive wakefulness syndrome patients. Nat. Commun. *9*, 4427. https://doi.org/10.1038/s41467-018-06871-1.
20. Arena, A., Comolatti, R., Thon, S., Casali, A.G., and Storm, J.F. (2021). General Anesthesia Disrupts Complex Cortical Dynamics in Response to Intracranial Electrical Stimulation in Rats. eNeuro *8*, ENEURO.0343-20.2021. https://doi.org/10.1523/ENEURO.0343-20.2021.
21. Cattani, A., Galluzzi, A., Fecchio, M., Pigorini, A., Mattia, M., and Massimini, M. (2023). Adaptation Shapes Local Cortical Reactivity: From Bifurcation Diagram and Simulations to Human Physiological and Pathological Responses. eNeuro *10*, ENEURO.0435-22.2023. https://doi.org/10.1523/ENEURO.0435-22.2023.
22. Massimini, M., Corbetta, M., Sanchez-Vives, M.V., Andrillon, T., Deco, G., Rosanova, M., and Sarasso, S. (2024). Sleep-like cortical dynamics during wakefulness and their network effects following brain injury. Nat. Commun. *15*, 7207. https://doi.org/10.1038/s41467-024-51586-1.
23. Nartallo-Kaluarachchi, R., Asllani, M., Deco, G., Kringelbach, M.L., Goriely, A., and Lambiotte, R. (2024). Broken detailed balance and entropy production in directed networks. Phys. Rev. E *110*, 034313. https://doi.org/10.1103/PhysRevE.110.034313.
24. Kringelbach, M.L., Sanz Perl, Y., and Deco, G. (2024). The Thermodynamics of Mind. Trends Cogn. Sci. *28*, 568–581. https://doi.org/10.1016/j.tics.2024.03.009.
25. Sanz Perl, Y., Bocaccio, H., Pallavicini, C., Pérez-Ipiña, I., Laureys, S., Laufs, H., Kringelbach, M., Deco, G., and Tagliazucchi, E. (2021). Nonequilibrium brain dynamics as a signature of consciousness. Phys. Rev. E *104*, 14411. https://doi.org/10.1103/physreve.104.014411.
26. Deco, G., Sanz Perl, Y., De La Fuente, L., Sitt, J.D., Yeo, B.T.T., Tagliazucchi, E., and Kringelbach, M.L. (2023). The arrow of time of brain signals in cognition: Potential intriguing role of parts of the default mode network. Netw. Neurosci. *7*, 966–998. https://doi.org/10.1162/netn_a_00300.
27. Deco, G., Sanz Perl, Y., Bocaccio, H., Tagliazucchi, E., and Kringelbach, M.L. (2022). The INSIDEOUT framework provides precise signatures of the balance of intrinsic and extrinsic dynamics in brain states. Commun. Biol. *5*, 572. https://doi.org/10.1038/s42003-022-03505-7.
28. Camassa, A., Torao-Angosto, M., Manasanch, A., Kringelbach, M.L., Deco, G., and Sanchez-Vives, M.V. (2024). The temporal asymmetry of cortical dynamics as a signature of brain states. Sci. Rep. *14*, 24271. https://doi.org/10.1038/s41598-024-74649-1.
29. Lynn, C.W., Cornblath, E.J., Papadopoulos, L., Bertolero, M.A., and Bassett, D.S. (2021). Broken detailed balance and entropy production in the human brain. Proc. Natl. Acad. Sci. *118*, e2109889118. https://doi.org/10.1073/pnas.2109889118.
30. Kubo, R. (1957). Statistical-Mechanical Theory of Irreversible Processes. I. General Theory and Simple Applications to Magnetic and Conduction Problems. J. Phys. Soc. Jpn. *12*, 570–586. https://doi.org/10.1143/JPSJ.12.570.
31. Deco, G., Lynn, C.W., Sanz Perl, Y., and Kringelbach, M.L. (2023). Violations of the fluctuation-dissipation theorem reveal distinct nonequilibrium dynamics of brain states. Phys. Rev. E *108*, 064410. https://doi.org/10.1103/PhysRevE.108.064410.

32. Monti, J.M., Perl, Y.S., Tagliazucchi, E., Kringelbach, M.L., and Deco, G. (2025). Fluctuation-dissipation theorem and the discovery of distinctive off-equilibrium signatures of brain states. Phys. Rev. Res. *7*, 013301. https://doi.org/10.1103/PhysRevResearch.7.013301.

33. Stikvoort, W., Pérez-Ordoyo, E., Mindlin, I., Escrichs, A., Sitt, J.D., Kringelbach, M.L., Deco, G., and Perl, Y.S. (2025). Nonequilibrium brain dynamics elicited as the origin of perturbative complexity. PLoS Comput. Biol. *21*, e1013150. https://doi.org/10.1371/journal.pcbi.1013150.

34. Colombo, M.A., Comanducci, A., Casarotto, S., Derchi, C.-C., Annen, J., Viganò, A., Mazza, A., Trimarchi, P.D., Boly, M., Fecchio, M., et al. (2023). Beyond alpha power: EEG spatial and spectral gradients robustly stratify disorders of consciousness. Cereb. Cortex *33*, 7193–7210. https://doi.org/10.1093/cercor/bhad031.

35. Dasilva, M., Camassa, A., Navarro-Guzman, A., Pazienti, A., Perez-Mendez, L., Zamora-López, G., Mattia, M., and Sanchez-Vives, M.V. (2021). Modulation of cortical slow oscillations and complexity across anesthesia levels. Neuroimage *224*, 117415. https://doi.org/10.1016/j.neuroimage.2020.117415.

36. Gilson, M., Tagliazucchi, E., and Cofré, R. (2023). Entropy production of Multivariate Ornstein-Uhlenbeck processes correlates with consciousness levels in the human brain. Phys. Rev. E *107*, 24121. https://doi.org/10.1103/PhysRevE.107.024121.

37. Ignaccolo, M., Latka, M., Jernajczyk, W., Grigolini, P., and West, B.J. (2010). Dynamics of electroencephalogram entropy and pitfalls of scaling detection. Phys. Rev. E *81*, 31909. https://doi.org/10.1103/physreve.81.031909.

38. Gilson, M., Zamora-López, G., Pallarés, V., Adhikari, M.H., Senden, M., Campo, A.T., Mantini, D., Corbetta, M., Deco, G., and Insabato, A. (2020). Model-based whole-brain effective connectivity to study distributed cognition in health and disease. Netw. Neurosci. *4*, 338–373. https://doi.org/10.1162/netn_a_00117.

39. Cugliandolo, L.F., Dean, D.S., and Kurchan, J. (1997). Fluctuation-Dissipation theorems and entropy production in relaxational systems. Phys. Rev. Lett. *79*, 2168–2171. https://doi.org/10.1103/PhysRevLett.79.2168.

40. Cugliandolo, L.F., Kurchan, J., and Parisi, G. (1994). Off equilibrium dynamics and aging in unfrustrated systems. J. Phys. *4*, 1641–1656. https://doi.org/10.1051/jp1:1994212.

41. Ruiz-Mejias, M., Ciria-Suarez, L., Mattia, M., and Sanchez-Vives, M.V. (2011). Slow and fast rhythms generated in the cerebral cortex of the anesthetized mouse. J. Neurophysiol. *106*, 2910–2921. https://doi.org/10.1152/jn.00440.2011.

42. Sanchez-Vives, M.V. (2020). Origin and dynamics of cortical slow oscillations. Curr. Opin. Physiol. *15*, 217–223. https://doi.org/10.1016/j.cophys.2020.04.005.

43. Camassa, A., Galluzzi, A., Mattia, M., and Sanchez-Vives, M.V. (2022). Deterministic and Stochastic Components of Cortical Down States: Dynamics and Modulation. J. Neurosci. *42*, 9387–9400. https://doi.org/10.1523/JNEUROSCI.0914-22.2022.

44. Garfield, J.M., Garfield, F.B., Stone, J.G., Hopkins, D., and Johns, L.A. (1972). A Comparison of Psychologic Responses to Ketamine and Thiopental–Nitrous Oxide–Halothane Anesthesia. Anesthesiology *36*, 329–338. https://doi.org/10.1097/00000542-197204000-00006.

45. Drummond, J.C., Brebner, J., Galloon, S., and Young, P.S. (1979). A randomized evaluation of the reversal of ketamine by physostigmine. Can. Anaesth. Soc. J. *26*, 288–295. https://doi.org/10.1007/BF03006289.

46. Massimini, M., Ferrarelli, F., Huber, R., Esser, S.K., Singh, H., and Tononi, G. (2005). Breakdown of Cortical Effective Connectivity during Sleep. Science *309*, 2228–2232. https://doi.org/10.1126/science.1117256.

47. Sarasso, S., Rosanova, M., Casali, A.G., Casarotto, S., Fecchio, M., Boly, M., Gosseries, O., Tononi, G., Laureys, S., and Massimini, M. (2014). Quantifying Cortical EEG Responses to TMS in (Un)consciousness. Clin. EEG Neurosci. *45*, 40–49. https://doi.org/10.1177/1550059413513723.

48. Koch, C., Massimini, M., Boly, M., and Tononi, G. (2016). Neural correlates of consciousness: progress and problems. Nat. Rev. Neurosci. *17*, 307–321. https://doi.org/10.1038/nrn.2016.22.

49. Engemann, D.A., Raimondo, F., King, J.-R., Rohaut, B., Louppe, G., Faugeras, F., Annen, J., Cassol, H., Gosseries, O., Fernandez-Slezak, D., et al. (2018). Robust EEG-based cross-site and cross-protocol classification of states of consciousness. Brain *141*, 3179–3192. https://doi.org/10.1093/brain/awy251.

50. Schiff, N.D. (2016). Mesocircuit Mechanisms Underlying Recovery of Consciousness Following Severe Brain Injuries: Model and Predictions. In Brain Function and Responsiveness in Disorders of Consciousness, M.M. Monti and W.G. Sannita, eds. (Springer International Publishing), pp. 195–204. https://doi.org/10.1007/978-3-319-21425-2_15.

51. Fischer, C., Luaute, J., and Morlet, D. (2010). Event-related potentials (MMN and novelty P3) in permanent vegetative or minimally conscious states. Clin. Neurophysiol. *121*, 1032–1042. https://doi.org/10.1016/j.clinph.2010.02.005.

52. Höller, Y., Bergmann, J., Kronbichler, M., Crone, J.S., Schmid, E.V., Golaszewski, S., and Ladurner, G. (2011). Preserved oscillatory response but lack of mismatch negativity in patients with disorders of consciousness. Clin. Neurophysiol. *122*, 1744–1754. https://doi.org/10.1016/j.clinph.2011.02.009.

53. Silverstein, B.H., Snodgrass, M., Shevrin, H., and Kushwaha, R. (2015). P3b, consciousness, and complex unconscious processing. Cortex *73*, 216–227. https://doi.org/10.1016/j.cortex.2015.09.004.

54. Forgacs, P.B., Conte, M.M., Fridman, E.A., Voss, H.U., Victor, J.D., and Schiff, N.D. (2014). Preservation of electroencephalographic organization in patients with impaired consciousness and imaging-based evidence of command-following. Ann. Neurol. *76*, 869–879. https://doi.org/10.1002/ana.24283.

55. Muthukumaraswamy, S.D., and Liley, D.T. (2018). 1/f electrophysiological spectra in resting and drug-induced states can be explained by the dynamics of multiple oscillatory relaxation processes. Neuroimage *179*, 582–595. https://doi.org/10.1016/j.neuroimage.2018.06.068.

56. Tononi, G., Boly, M., Massimini, M., and Koch, C. (2016). Integrated information theory: from consciousness to its physical substrate. Nat. Rev. Neurosci. *17*, 450–461. https://doi.org/10.1038/nrn.2016.44.

57. Tononi, G. (2004). An information integration theory of consciousness. BMC Neurosci. *5*, 42. https://doi.org/10.1186/1471-2202-5-42.

58. Tononi, G., Sporns, O., and Edelman, G.M. (1994). A measure for brain complexity: relating functional segregation and integration in the nervous system. Proc. Natl. Acad. Sci. *91*, 5033–5037. https://doi.org/10.1073/pnas.91.11.5033.

59. Andrillon, T., Poulsen, A.T., Hansen, L.K., Léger, D., and Kouider, S. (2016). Neural Markers of Responsiveness to the Environment in Human Sleep. J. Neurosci. *36*, 6583–6596. https://doi.org/10.1523/JNEUROSCI.0902-16.2016.

60. Ferenets, R., Lipping, T., Anier, A., Jäntti, V., Melto, S., and Hovilehto, S. (2006). Comparison of Entropy and Complexity Measures for the Assessment of Depth of Sedation. IEEE Trans. Biomed. Eng. *53*, 1067–1077. https://doi.org/10.1109/TBME.2006.873543.

61. Ferenets, R., Vanluchene, A., Lipping, T., Heyse, B., and Struys, M.M.R.F. (2007). Behavior of Entropy/Complexity Measures of the Electroencephalogram during Propofol-induced Sedation: Dose-dependent Effects of Remifentanil. Anesthesiology *106*, 696–706. https://doi.org/10.1097/01.anes.0000264790.07231.2d.

62. Schartner, M., Seth, A., Noirhomme, Q., Boly, M., Bruno, M.-A., Laureys, S., and Barrett, A. (2015). Complexity of Multi-Dimensional Spontaneous EEG Decreases during Propofol Induced General Anaesthesia. PLoS One *10*, e0133532. https://doi.org/10.1371/journal.pone.0133532.

63. Maschke, C., O'Byrne, J., Colombo, M.A., Boly, M., Gosseries, O., Laureys, S., Rosanova, M., Jerbi, K., and Blain-Moraes, S. (2024). Critical dynamics in spontaneous EEG predict anesthetic-induced loss of

consciousness and perturbational complexity. Commun. Biol. *7*, 946. https://doi.org/10.1038/s42003-024-06613-8.

64. Cloostermans, M.C., Van Meulen, F.B., Eertman, C.J., Hom, H.W., and Van Putten, M.J.A.M. (2012). Continuous electroencephalography monitoring for early prediction of neurological outcome in postanoxic patients after cardiac arrest: A prospective cohort study. Crit. Care Med. *40*, 2867–2875. https://doi.org/10.1097/CCM.0b013e31825b94f0.

65. Rossi Sebastiano, D., Panzica, F., Visani, E., Rotondi, F., Scaioli, V., Leonardi, M., Sattin, D., D'Incerti, L., Parati, E., Ferini Strambi, L., and Franceschetti, S. (2015). Significance of multiple neurophysiological measures in patients with chronic disorders of consciousness. Clin. Neurophysiol. *126*, 558–564. https://doi.org/10.1016/j.clinph.2014.07.004.

66. Rossetti, A.O., Rabinstein, A.A., and Oddo, M. (2016). Neurological prognostication of outcome in patients in coma after cardiac arrest. Lancet Neurol. *15*, 597–609. https://doi.org/10.1016/S1474-4422(16)00015-6.

67. Hofmeijer, J., and Van Putten, M.J.A.M. (2016). EEG in postanoxic coma: Prognostic and diagnostic value. Clin. Neurophysiol. *127*, 2047–2055. https://doi.org/10.1016/j.clinph.2016.02.002.

68. Deco, G., Cruzat, J., Cabral, J., Tagliazucchi, E., Laufs, H., Logothetis, N.K., and Kringelbach, M.L. (2019). Awakening: Predicting external stimulation to force transitions between different brain states. Proc. Natl. Acad. Sci. *116*, 18088–18097. https://doi.org/10.1073/pnas.1905534116.

69. Edlow, B.L., Claassen, J., Schiff, N.D., and Greer, D.M. (2021). Recovery from disorders of consciousness: mechanisms, prognosis and emerging therapies. Nat. Rev. Neurol. *17*, 135–156. https://doi.org/10.1038/s41582-020-00428-x.

70. Sanchez-Vives, M.V., Massimini, M., and Mattia, M. (2017). Shaping the Default Activity Pattern of the Cortical Network. Neuron *94*, 993–1001. https://doi.org/10.1016/j.neuron.2017.05.015.

71. Patow, G., Monti, J., Acero-Pousa, I., Idesis, S., Escrichs, A., Perl, Y.S., Ritter, P., Kringelbach, M., and Deco, G.; the Alzheimer's Disease Neuroimaging Initiative (2024). Off-Equilibrium Fluctuation-Dissipation Theorem Paves the Way in Alzheimer's Disease Research. preprint at bioRxiv. https://doi.org/10.1101/2024.09.15.613131.

72. Piarulli, A., Bergamasco, M., Thibaut, A., Cologan, V., Gosseries, O., and Laureys, S. (2016). EEG ultradian rhythmicity differences in disorders of consciousness during wakefulness. J. Neurol. *263*, 1746–1760. https://doi.org/10.1007/s00415-016-8196-y.

73. Mertel, I., Pavlov, Y.G., Barner, C., Müller, F., Diekelmann, S., and Kotchoubey, B. (2020). Sleep in disorders of consciousness: behavioral and polysomnographic recording. BMC Med. *18*, 350. https://doi.org/10.1186/s12916-020-01812-6.

74. Colombo, M.A., Napolitani, M., Boly, M., Gosseries, O., Casarotto, S., Rosanova, M., Brichant, J.-F., Boveroux, P., Rex, S., Laureys, S., et al. (2019). The spectral exponent of the resting EEG indexes the presence of consciousness during unresponsiveness induced by propofol, xenon, and ketamine. Neuroimage *189*, 631–644. https://doi.org/10.1016/j.neuroimage.2019.01.024.

75. Sanchez-Vives, M. (2020). Propagation modes of slow waves in mouse cortex. EBRAINS. https://doi.org/10.25493/WKA8-Q4T.

76. Massimini, M., and Laureys, S. (2017). Rest EEG Recordings in Healthy Subjects during Wakefulness, Sleep and Anesthesia with Ketamine, Propofol, and Xenon (Zenodo). https://doi.org/10.5281/ZENODO.806176.

77. Tort-Colet, N., Capone, C., Sanchez-Vives, M.V., and Mattia, M. (2021). Attractor competition enriches cortical dynamics during awakening from anesthesia. Cell Rep. *35*, 109270. https://doi.org/10.1016/j.celrep.2021.109270.

78. Pazzini, L., Polese, D., Weinert, J.F., Maiolo, L., Maita, F., Marrani, M., Pecora, A., Sanchez-Vives, M.V., and Fortunato, G. (2018). An ultracompact integrated system for brain activity recording and stimulation validated over cortical slow oscillations in vivo and in vitro. Sci. Rep. *8*, 16717. https://doi.org/10.1038/s41598-018-34560-y.

79. Ramsay, M.A., Savege, T.M., Simpson, B.R., and Goodwin, R. (1974). Controlled Sedation with Alphaxalone-Alphadolone. BMJ *2*, 656–659. https://doi.org/10.1136/bmj.2.5920.656.

80. Murphy, M., Bruno, M.-A., Riedner, B.A., Boveroux, P., Noirhomme, Q., Landsness, E.C., Brichant, J.-F., Phillips, C., Massimini, M., Laureys, S., et al. (2011). Propofol Anesthesia and Sleep: A High-Density EEG Study. Sleep *34*, 283. https://doi.org/10.1093/sleep/34.3.283.

81. Rex, S., Schaefer, W., Meyer, P.H., Rossaint, R., Boy, C., Setani, K., Büll, U., and Baumert, J.H. (2006). Positron Emission Tomography Study of Regional Cerebral Metabolism during General Anesthesia with Xenon in Humans. Anesthesiology *105*, 936–943. https://doi.org/10.1097/00000542-200611000-00014.

82. Lee, U., Ku, S., Noh, G., Baek, S., Choi, B., and Mashour, G.A. (2013). Disruption of Frontal–Parietal Communication by Ketamine, Propofol, and Sevoflurane. Anesthesiology *118*, 1264–1275. https://doi.org/10.1097/ALN.0b013e31829103f5.

83. Hockaday, J.M., Potts, F., Epstein, E., Bonazzi, A., and Schwab, R.S. (1965). Electroencephalographic changes in acute cerebral anoxia from cardiac or respiratory arrest. Electroencephalogr. Clin. Neurophysiol. *18*, 575–586. https://doi.org/10.1016/0013-4694(65)90075-1.

84. Estraneo, A., Loreto, V., Guarino, I., Boemia, V., Paone, G., Moretta, P., and Trojano, L. (2016). Standard EEG in diagnostic process of prolonged disorders of consciousness. Clin. Neurophysiol. *127*, 2379–2385. https://doi.org/10.1016/j.clinph.2016.03.021.

85. Reig, R., Mattia, M., Compte, A., Belmonte, C., and Sanchez-Vives, M.V. (2010). Temperature Modulation of Slow and Fast Cortical Rhythms. J. Neurophysiol. *103*, 1253–1261. https://doi.org/10.1152/jn.00890.2009.

86. Sanchez-Vives, M.V., Mattia, M., Compte, A., Perez-Zabalza, M., Winograd, M., Descalzo, V.F., and Reig, R. (2010). Inhibitory Modulation of Cortical Up States. J. Neurophysiol. *104*, 1314–1324. https://doi.org/10.1152/jn.00178.2010.

87. Mattia, M., and Del Giudice, P. (2002). Population dynamics of interacting spiking neurons. Phys. Rev. E *66*, 051917. https://doi.org/10.1103/PhysRevE.66.051917.

88. Wilson, H.R., and Cowan, J.D. (1973). A mathematical theory of the functional dynamics of cortical and thalamic nervous tissue. Kybernetik *13*, 55–80. https://doi.org/10.1007/BF00288786.

89. Wang, M.C., and Uhlenbeck, G.E. (1945). On the Theory of the Brownian Motion II. Rev. Mod. Phys. *17*, 323–342. https://doi.org/10.1103/revmodphys.17.323.

90. Godrèche, C., and Luck, J.-M. (2019). Characterising the nonequilibrium stationary states of Ornstein-Uhlenbeck processes. J. Phys. A: Math. Theor. *52*, 035002. https://doi.org/10.1088/1751-8121/aaf190.

91. Uhlenbeck, G.E., and Ornstein, L.S. (1930). On the Theory of the Brownian Motion. Phys. Rev. *36*, 823–841. https://doi.org/10.1103/PhysRev.36.823.

92. Hsu, M., and Hsu, D. (2005). A piece-wise harmonic Langevin model of EEG dynamics: Theory and application to EEG seizure detection. Neurocomputing *65–66*, 469–474. https://doi.org/10.1016/j.neucom.2004.11.003.

93. Endemann, C.M., Krause, B.M., Nourski, K.V., Banks, M.I., and Veen, B.V. (2022). Multivariate autoregressive model estimation for high-dimensional intracranial electrophysiological data. Neuroimage *254*, 119057. https://doi.org/10.1016/j.neuroimage.2022.119057.

94. Anderson, C.W., Stolz, E.A., and Shamsunder, S. (1998). Multivariate autoregressive models for classification of spontaneous electroencephalographic signals during mental tasks. IEEE Trans. Biomed. Eng. *45*, 277–286. https://doi.org/10.1109/10.661153.

95. Gilson, M., Deco, G., Friston, K.J., Hagmann, P., Mantini, D., Betti, V., Romani, G.L., and Corbetta, M. (2018). Effective connectivity inferred from fMRI transition dynamics during movie viewing points to a balanced

CellPress
OPEN ACCESS

reconfiguration of cortical interactions. Neuroimage *180*, 534–546. https://doi.org/10.1016/j.neuroimage.2017.09.061.

96. Deco, G., Sanz Perl, Y., Johnson, S., Bourke, N., Carhart-Harris, R.L., and Kringelbach, M.L. (2024). Different hierarchical reconfigurations in the brain by psilocybin and escitalopram for depression. Nat. Ment. Health *2*, 1096–1110. https://doi.org/10.1038/s44220-024-00298-y.

97. Gilson, M., Moreno-Bote, R., Ponce-Alvarez, A., Ritter, P., and Deco, G. (2016). Estimation of Directed Effective Connectivity from fMRI Functional Connectivity Hints at Asymmetries of Cortical Connectome. PLoS Comput. Biol. *12*, e1004762. https://doi.org/10.1371/journal.pcbi.1004762.

98. Kringelbach, M.L., Perl, Y.S., Tagliazucchi, E., and Deco, G. (2023). Toward naturalistic neuroscience: Mechanisms underlying the flattening of brain hierarchy in movie-watching compared to rest and task. Sci. Adv. *9*, eade6049. https://doi.org/10.1126/sciadv.ade6049.

99. Altaner, B., Polettini, M., and Esposito, M. (2016). Fluctuation-Dissipation Relations Far from Equilibrium. Phys. Rev. Lett. *117*, 180601. https://doi.org/10.1103/PhysRevLett.117.180601.

100. Ohga, N., Ito, S., and Kolchinsky, A. (2023). Thermodynamic Bound on the Asymmetry of Cross-Correlations. Phys. Rev. Lett. *131*, 077101. https://doi.org/10.1103/PhysRevLett.131.077101.

STAR★METHODS

KEY RESOURCES TABLE

REAGENT or RESOURCE	SOURCE	IDENTIFIER
Deposited data		
Mouse LFP recordings across graded levels of isoflurane anesthesia	Sanchez-Vives, M.V.[75]	EBRAINS: https://doi.org/10.25493/WKA8-Q4T
Human EEG recordings during wakefulness and anesthesia	Massimini, M. and Laureys, S.[76]	Zenodo: https://doi.org/10.5281/zenodo.806176; restricted access; access requests are assessed by the relevant data custodians.
Experimental models: organisms/strains		
Mouse: adult male C57BL/6J	Sanchez-Vives, M.V.[75]	C57BL/6J
Human: healthy adult participants recorded during wakefulness and anesthesia	Massimini, M. and Laureys, S.[76]	N/A
Human: patients with disorders of consciousness	Casarotto et al.[15]; Colombo et al.[34]	N/A
Software and algorithms		
Custom code and analyses	This paper	Zenodo: https://doi.org/10.5281/zenodo.20719663 GitHub: https://github.com/tomasberjagabuisan/Thermo-Consciousness
MATLAB	MathWorks	R2023b; RRID: SCR_001622
Python	Python Software Foundation	Version 3.13; RRID: SCR_008394

EXPERIMENTAL MODEL AND STUDY PARTICIPANT DETAILS

Mouse electrophysiological data

Animal subjects

Data were obtained from a previously published study.[35] Recordings were conducted in eight adult male C57BL/6J mice bred in-house at the University of Barcelona and maintained on a 12 h light/dark cycle with *ad libitum* access to food and water. All procedures were approved by the Ethics Committee of the Hospital Clinic of Barcelona and adhered to Spanish regulatory laws (BOE 34/11370–421, 2013) and the European Union directive 2010/63/EU. Detailed methodological procedures can be found in the original publication.[35]

Surgical procedures and anesthesia protocol

Anesthesia was induced via intraperitoneal injection of ketamine (75 mg/kg) and medetomidine (1.3 mg/kg) and maintained with isoflurane in pure oxygen. Atropine (0.3 mg/kg), methylprednisolone (30 mg/kg), and mannitol (0.5 g/kg) were administered subcutaneously to reduce respiratory secretions and prevent cerebral edema. Body temperature was monitored and maintained at 37°C using a thermal blanket (RWD Life Science, China).

Mice were placed in a stereotaxic frame (SR-6M, Narishige, Japan), and a craniotomy and durotomy (−3.0 mm to +3.0 mm relative to bregma and +3.0 mm lateral to midline) was performed over one hemisphere. Three graded levels of anesthesia were defined based on isoflurane concentrations: Deep = 1.16% ± 0.08 s.e.; Mid = 0.34% ± 0.06 s.e.; and Light = 0–0.1%. The volume delivered was 0.8 L/min and the animals were breathing freely via a tracheostomy. Each level was maintained for 20–30 min, and recordings were taken during a stable slow-oscillatory regime (~10 min post-concentration change, before microarousals).[77] Reflexes were regularly tested to confirm anesthetic depth.

Electrophysiological recordings

Extracellular LFP activity was recorded using 32-channel multielectrode arrays (550 μm interelectrode spacing) covering the entire exposed area of the cortex.[78] Signals were amplified by 100, high-pass filtered above 0.1 Hz, digitized at 5 kHz, and fed into a computer via a digitizer interface (CED 1401 and Spike2 software, Cambridge Electronic Design, UK).

For each anesthesia level, 300–500 s of spontaneous activity was recorded, followed by an equal duration of evoked activity. For further methodological details on the stimulation setup and protocol, readers are referred to the original publication where these procedures are described in detail.[35]

Human EEG data during wakefulness and anesthesia

Participants

Data were obtained from a previously published dataset[76] comprising fifteen healthy participants (5 males, aged 18–28 years) recorded during wakefulness with eyes-open or eyes-closed conditions. Participants were randomly assigned to one of three anesthetic conditions (N = 5 per condition: xenon, propofol, and ketamine), resulting in a total of 30 recordings.[74] All participants underwent medical screening to rule out contraindications to anesthesia and provided written informed consent. The experimental protocol was approved by the Ethics Committee of the University of Liège, Belgium. This dataset has been validated and widely used in prior research.[15,63,74] For full methodological details, refer to the original publication.[74]

Experimental design and anesthetic procedures

All experimental procedures were performed at the Center Hospitalier Universitaire in Liège, Belgium. Spontaneous EEG, followed by TMS-EEG recordings, was recorded before drug administration (Ramsay Scale score 2) as well as upon reaching deep unresponsiveness in three consecutive assessments (Ramsay Scale score 6).[79] At the end of the TMS-EEG recordings, anesthesia was discontinued, participants were allowed to recover, and assessments of responsiveness were continued every 30 s. Participants were continuously monitored for heart rate, oxygen saturation, blood pressure, exhaled CO_2, and temperature. Mild nausea, if present, was treated with metoclopramide (2 mg).

To enable comparisons across drugs with distinct molecular targets, all anesthetic protocols aimed to reach a common behavioral endpoint: unresponsiveness to external stimuli systematically assessed using repeated Ramsay Scale administrations. Anesthetic protocols followed established procedures for propofol,[80] xenon,[81] and ketamine.[82]

TMS–EEG acquisition

EEG data were recorded using a 60-channel TMS-compatible amplifier (Nexstim Plc., Finland), with online filtering (0.1–350 Hz) and sampling at 1450 Hz. The reference electrode was placed on the forehead, and the impedance was kept below 5 kΩ. Two additional channels recorded electrooculographic activity. For further methodological details on the TMS–EEG acquisition, stimulation, and navigation procedures, readers are referred to the original studies where these methods are described in detail.[74]

Assessment of conscious experience

To assess the presence or absence of conscious experience during anesthesia-induced unresponsiveness, retrospective reports were collected in all participants after awakening. Responsiveness was considered restored when participants consistently reached a Ramsay score of 2 on three consecutive assessments. At that point, they were asked whether they recalled any mental activity during the unresponsive state. Conscious experience was defined broadly as any kind of mental activity, including thoughts, dreams, images, or emotions. Under propofol and xenon, participants either reported no memory of the period or were unable to recall any experience. In contrast, all participants who received ketamine described vivid, emotionally intense experiences with a coherent narrative structure, consistent with previous findings.[6]

Human EEG data in disorders of consciousness

Patients

Data were gathered from previously published studies[15,34] and included 26 patients diagnosed with prolonged or chronic DoC. Patients were clinically classified as being in UWS, MCS−, or MCS+ based on repeated CRS-R assessments conducted at least three times within one week. The main analyses were performed on the 17 non-anoxic patients, comprising vascular (N = 13) and traumatic (N = 4) etiologies, while the post-anoxic subgroup was examined separately in Note S3. Patients with post-anoxic etiology were not included in the main analysis because, in severe cases, their electrophysiological signatures primarily reflect cortical integrity rather than consciousness capacity.[34] These patients often show marked suppression of alpha power within a context of overall power suppression and low-voltage EEG, in which both spontaneous and perturbational EEG measures may become strongly constrained by cortical integrity rather than providing a graded index of consciousness capacity.[64–67,83,84]

Written informed consent was obtained either from the patients (when possible) or from their legal representatives. All experimental procedures were approved by the respective local ethics committees. All patients were free of sedative medication at the time of recording. Further methodological details can be found in the original publications.[15,34]

Experimental design

During recordings, patients were seated in an upright or reclined position in a quiet room. Short spontaneous EEG recordings (median duration = 5.13 min, interquartile range: 4.03–6.59 min) were acquired either during the same experimental session of TMS-EEG recordings (N = 14) or, when not feasible, during a closely timed session within one day (N = 1) or at most within two weeks (N = 2). In all cases, the CRS-R behavioral diagnosis remained stable across sessions. Patients were continuously monitored by trained personnel to detect any signs of drowsiness. When necessary, the CRS-R vigilance protocol[2] was administered to minimize the risk of sleep intrusions and reduce the potential confounding effects of arousal fluctuations on the variables of interest.

Importantly, the DoC analyses were performed blind to the patients' behavioral diagnosis, PCI values, and clinical information, which were used only afterward for group-level evaluation. In the DoC population, behavioral classification based on CRS-R yielded a natural dichotomy: behaviorally unresponsive patients (B−, i.e., UWS; N = 5) and behaviorally responsive patients (B+, i.e., MCS− and MCS+; N = 12).

TMS-EEG acquisition

EEG was recorded using Ag/AgCl electrodes and TMS-compatible amplifiers (Nexstim Plc., 60 channels, $N = 14$; Brain Products GmbH, 64 channels, $N = 3$). The two systems employed highly similar electrode montages (see Note S5; Figure S7), with a common acquisition reference placed on the forehead near Fpz. Electrode input impedance was maintained below 20 kΩ. For further methodological details on the TMS–EEG acquisition, stimulation, and navigation procedures, readers are referred to the original studies in which these methods are described in detail.[15,34]

Signal preprocessing

Animal electrophysiological signals

For rodent data, distinct signal components were extracted from the same LFP recordings. For PCI computation, multi-unit activity was estimated by computing the power in the 200–1500 Hz band using 5-ms windows, a range that captures fast population spiking associated with evoked responses.[41,85–87] Further methodological details can be found in the original publication.[35]

In contrast, FDT violations analyses were performed on spontaneous LFP activity filtered in the delta band (0.5–4 Hz) to isolate slow cortical oscillations, which dominate anesthesia (accounting for up to 95% of spectral power) and are particularly sensitive to global state changes.[42,70] These slow fluctuations evolve over extended timescales and display rich temporal and spatial structure, providing a natural substrate for assessing FDT violations. Importantly, this band choice was further supported by repeating the analysis using the broadband range employed for human data (see Note S6; Figure S8).

Human EEG signals

Unlike in rodents, human recordings spanned a broader range of brain states, including wakefulness, that involve additional spectral components such as alpha and beta rhythms. For this reason, broadband signals were preserved to capture the full repertoire of state-dependent dynamics.

Spontaneous EEG signals were bandpass filtered using a third-order, zero-phase Butterworth filter (0.5–60 Hz cutoffs) with the *filtfilt* function in MATLAB, and notch-filtered to remove 50 Hz line noise. Data were then downsampled to reduce computational demands (from 5000 Hz to 1000 Hz for Brain Products recordings, and from 1450 Hz to 725 Hz for Nexstim recordings).

A trained neurophysiologist manually inspected each recording to exclude segments contaminated with gross artifacts. This procedure retained a median of 4.79 min of clean data per condition (98.74%, interquartile range: 3.58–6.12 min, 91.06–99.27%) and excluded a median of 2 electrodes per subject (IQR: 1–4). Rejected electrodes were interpolated using spherical splines, and all signals were re-referenced to the common average. To remove residual non-neural activity, independent component analysis was performed. Components attributable to ocular, muscular, or cardiac artifacts were visually identified and excluded. A median of 33 components (IQR: 25–41.5) were retained and back-projected to reconstruct artifact-free EEG signals.

To compute FDT violations, the cleaned EEG data were additionally low-pass filtered at 40 Hz and downsampled to match the corresponding Nyquist frequency, thereby minimizing high-frequency muscle artifacts and reducing computational demands during whole-brain model fitting.

Further details on recording setups and artifact removal procedures are provided in Note S5. For methodological details on the TMS-EEG preprocessing, please refer to the original studies.[6,15,34] For illustration, representative TMS–EEG traces corresponding to high and low PCI values are shown in Note S7; Figure S9.

METHOD DETAILS

Perturbational Complexity Index

PCI values were obtained from previous works.[6,15,34,35] PCI quantifies the complexity of spatiotemporal brain patterns in response to external perturbations, typically delivered via TMS or intracranial stimulation. Complexity is measured using the lossless Lempel-Ziv (LZ) data compression algorithm, normalized by the source entropy.[10] To compute the LZ complexity, TMS-evoked responses are first binarized, producing a significant spatiotemporal matrix. This is achieved using a non-parametric permutation statistical procedure, as described by Casali et al. (2013).[10] The maximum PCI value across all stimulation sites (PCImax) is typically retained as the final score. Further methodological details for PCI computation in both humans and animal models can be found in the original studies.[6,15,34,35]

It should be noted that when PCI is applied to intracranial recordings and multi-unit activity, particularly in animal models, it is often referred to as intracranial multi-unit PCI. This variant extends the original index to accommodate multi-unit spike-based and intracortical perturbation paradigms.[16,35]

Whole-brain modeling

The local dynamics of each brain region were modeled as a multivariate Ornstein–Uhlenbeck (OU) process, which corresponds to the linearization of nonlinear population models such as Wilson–Cowan neurons.[88] This formulation provides a linear approximation to the stochastic dynamics of the system near a stable fixed point.[89,90] OU processes are particularly well-suited for capturing non-equilibrium dynamics, as they enable a direct and quantitative assessment of the relationship between spontaneous fluctuations and

linear response functions.[36,39,91] Moreover, OU-based models have been successfully applied to characterize large-scale brain dynamics in both EEG and fMRI studies, and have proven effective in extracting biologically meaningful structure from spontaneous activity.[36–38]

In this framework, brain activity is modeled as a network of interacting regions governed by coupled stochastic differential equations:

$$\frac{dx}{dt} = -Bx(t) + \eta(t) \quad \text{(Equation 1)}$$

where B is the drift matrix and corresponds to the asymmetric connectivity matrix inferred from empirical data. The $\eta(t)$ term corresponds to zero-mean additive white Gaussian noise. The noise is characterized by its covariance structure, $\langle\eta(t)\eta(t')\rangle = 2\sigma^2\delta(t-t')$, where σ^2 is, by definition, the noise variance.

To ensure a well-defined and stable stationary solution, we require that all eigenvalues of the drift matrix B have strictly positive real parts.[90] This condition guarantees that the associated system dynamics fulfills the Hurwitz criterion, meaning the system naturally decays and converges to a unique steady state. This requirement is essential not only for mathematical stability and well-posedness, but also for the thermodynamic and biological interpretability of the model.[90] To assess whether local weak stationarity provided a reasonable approximation for the effective linear stochastic model, we performed an additional control analysis in which recordings were segmented into 10-s overlapping detrended epochs and tested for local weak stationarity using complementary Augmented Dickey–Fuller and Kwiatkowski–Phillips–Schmidt–Shin tests (see Note S8). This approach is consistent with the methodology and previous applications of the model.[37,92–94]

Model optimization

To quantify FDT violations, we applied the multivariate OU modeling framework described in the section above. The empirical observables used for model fitting included the functional connectivity matrix ($FC^{empirical}$), reflecting zero-lag statistical dependencies across brain regions, and the normalized time-lagged covariance matrix ($FS^{empirical}(\tau)$), which captures directional temporal interactions at a fixed lag while reducing zero-lag dependencies and volume-conduction effects. These normalized time-lagged covariance matrices are generated by taking the shifted covariance matrix $KS^{empirical}(\tau)$ and normalizing each pair (i, j) by dividing $\sqrt{KS_{ii}^{empirical}(0)KS_{jj}^{empirical}(0)}$. This normalization accounts for variance across regions and emphasizes relative lagged dependencies. Importantly, incorporating time-lagged covariances allows the estimated coupling matrix B to become asymmetric, enabling the emergence of non-equilibrium dynamics and quantifiable violations of the FDT.[95,96] The time lag τ was selected to ensure theoretical consistency with the OU formulation and to preserve sensitivity to temporal asymmetries, following previous studies.[36,38]

To match these empirical observables, the model was iteratively adjusted using a heuristic pseudo-gradient descent algorithm that minimized the difference between simulated and empirical FC and $FS(\tau)$ matrices.[38,97] We updated B until the fit was fully optimized and converged to a stable solution. More specifically, the updating uses the following rule:

$$B_{ij} = B_{ij} + \alpha\left(FC_{ij}^{empirical} - FC_{ij}^{model}\right) + \delta\left(FS_{ij}^{empirical}(\tau) - FS_{ij}^{model}(\tau)\right) \quad \text{(Equation 2)}$$

The multivariate OU model allows analytic computation of both stationary and time-lagged covariances. The stationary covariance matrix was defined as $K = \langle xx^T \rangle$ and the noise covariance as Q, assumed to be symmetric and positive semidefinite. Using Itô's calculus and keeping terms to first order in dt, the evolution of K is given by the following equation[31,32,96]:

$$\frac{dK}{dt} = -BK - KB^T + Q \quad \text{(Equation 3)}$$

At the steady state ($\frac{dK}{dt} = 0$), we obtain the continuous-time Lyapunov equation:

$$BK + KB^T = Q \quad \text{(Equation 4)}$$

Solving this equation (e.g., via Lyapunov or Sylvester solvers) provides the model-derived covariance matrix K, from which the simulated functional connectivity (FC^{model}) is computed. The time-lagged covariance was then calculated analytically as:

$$KS^{model}(\tau) = e^{-\tau B}K \quad \text{(Equation 5)}$$

and normalized in the same manner as the empirical data. Note that $KS^{model}(0) = K$.

B was initialized as a random symmetric matrix constrained to satisfy the Hurwitz stability criterion. This approach ensured mathematical stability without imposing anatomical priors. Learning rates were fixed at $\alpha = \delta = 0.00001$, and the optimization was repeated until the solution stabilized. The full optimization process was repeated 1000 times, and the results were then averaged to ensure convergence to a robust solution. We refer to the final optimized matrix as the generative effective connectivity matrix, which captures both the directional and non-equilibrium aspects of spontaneous brain dynamics.[98] Noise was assumed to be spatially homogeneous and therefore did not need to be fitted, as demonstrated in Note S9.

FDT violations

To assess the departure from equilibrium, we adapted a model-based formalism grounded in the off-equilibrium extension of FDT within a statistical-physics framework.[39,40,99] In thermodynamic equilibrium, the FDT states that the system's linear response to small perturbations is entirely determined by spontaneous fluctuations. However, in systems that are out of equilibrium, such as the brain, this relationship is broken. The degree of FDT violation therefore provides a principled model-based measure of effective non-equilibrium dynamics.[31,32]

More precisely, in equilibrium, the linear response function $R(t,t')$ of the system's state $x(t)$ to a small enough perturbation $\epsilon(t')$ is defined as[30]:

$$x(t) = \int_{t'}^{t} R(t,t')\epsilon(t')dt' \quad \text{(Equation 6)}$$

According to the FDT, this response function is directly related to the autocorrelation function of spontaneous fluctuations, $C(t,t') = \langle x(t)x(t')\rangle$, through the expression:

$$R(t,t') = \beta \frac{\partial C(t,t')}{\partial t'} \quad \text{(Equation 7)}$$

where $t' \leq t$ and $\beta = 1/T$, T being the temperature of the thermal bath. In the case of non-equilibrium systems, this equality no longer holds. Instead, the linear response can be determined through generalized expressions such as[40]:

$$R(t,t') = \frac{1}{2T}\langle x(t)\eta(t')\rangle \quad \text{(Equation 8)}$$

Based on this, two metrics that capture the degree of FDT violation have been introduced in prior studies[32,39]: the differential violation $V(t,t')$ and the integral violation $I(t,t')$.

$$V(t,t') = \frac{\partial C(t,t')}{\partial t'} - TR(t,t') \quad \text{(Equation 9)}$$

$$I(t,t') = \int_{t'}^{t} V(t,s)ds = C(t,t) - C(t,t') - T\,\chi(t,t') \quad \text{(Equation 10)}$$

where $\chi(t,t') = \int_{t'}^{t} R(t,s)ds$ is the integrated response function, or dynamic susceptibility.

To obtain scale-free quantities that allow comparisons across datasets recorded in the same species with the same recording modality, we normalized the metrics by the instantaneous autocorrelation, consistent with previous thermodynamic metrics.[31,100]

This normalization choice is justified by strict scale invariance. Normalizing by the instantaneous autocorrelation, rather than by a fixed $C(0,0)$ or by α^2 alone, further accounts for node-wise heterogeneity and slow variance drifts, while remaining robust to preprocessing steps that rescale amplitudes (e.g., z-scoring within runs).

Alternative estimators of FDT violations exist and are theoretically equivalent given infinite data. However, finite-sample effects can lead to variability across formulations. Following previous literature, we report the most stable estimates using the definition of $I(t,t')$, as this formulation is less prone to numerical instability.[32]

To obtain node-level metrics of non-equilibrium, we computed the time-integrated absolute values of the violations:

$$FDT\ violations = \sum_{t'} \int |I_j(t,t')|dt \quad \text{(Equation 11)}$$

Then, for each subject, we averaged over all nodes as a more global indicator of distance from equilibrium. In the present study, we focus on these global averages. However, in conditions involving more spatially localized alterations, such as neurodegenerative or focal disorders, node-specific patterns may provide additional insight.[22]

QUANTIFICATION AND STATISTICAL ANALYSIS

Data preprocessing, whole-brain model fitting, and computation of FDT violations were performed in MATLAB R2023b. Statistical analyses and data visualization were performed in Python 3.13.

Statistical analysis

Group comparisons were conducted using non-parametric tests. For paired conditions, the Wilcoxon signed-rank test was used, followed by Holm–Bonferroni post hoc corrections. For unpaired groups, the Mann–Whitney U test was applied, and effect sizes were quantified using Cliff's δ, a distribution-free, non-parametric measure directly interpretable as the probability of superiority. Group separability was quantified using the AROC/AUC, computed directly from subject-level mean values using the equivalence between AUC and the Mann–Whitney U statistic.

To assess monotonic relationships between variables, Spearman's correlation coefficients (ρ) were calculated. Pearson's correlation coefficients (r) were also computed when relationships were assumed to be linear and variables approximately normally distributed. We tested whether the relationship between FDT violations and PCI differed across conditions using an ANCOVA that included the *FDT violations* × *Condition* interaction term. The significance of this interaction was further evaluated using a Freedman–Lane permutation test (10,000 permutations, within-condition shuffling) to obtain a permutation-based *p-value* under small-sample conditions.

In paired datasets, linear mixed-effects models (LME) were fitted to confirm the robustness of the associations between FDT violations and PCI. In mice, the LME controlled for anesthesia level (deep vs. mid vs. light), while in humans it controlled for anesthesia state (anesthetized vs. awake) and drug type (ketamine vs. propofol vs. xenon). In both cases, subject identity was included as a random intercept to account for repeated measurements.

Subject-level generalizability was assessed using leave-one-subject-out cross-validation. For each leave-one-subject-out fold, we fit a linear model relating PCI to FDT violations using the training subjects only and then used the fitted model to generate out-of-sample predictions of FDT violations for the held-out subject. Predictions and observations were compared across folds using Spearman and Pearson correlations.

Variables were standardized prior to regression to allow interpretation of effect sizes in standard deviation units. All *p-values* reported are two-tailed, and significance was defined at $p < 0.05$.

Cell Reports, Volume *45*

Supplemental information

Thermodynamics of consciousness: Non-equilibrium brain dynamics track conscious states

Tomas Berjaga-Buisan, Juan Manuel Monti, Martina Cortada, Michele A. Colombo, Sebastian M. Geli, Gianluca Gaglioti, Simone Sarasso, Morten L. Kringelbach, Maurizio Corbetta, Maria V. Sanchez-Vives, Marcello Massimini, Yonatan Sanz Perl, and Gustavo Deco

SUPPLEMENTARY INFORMATION

Supplementary Note 1. Illustrative spontaneous traces of electrophysiological recordings

To qualitatively illustrate the temporal structure of brain activity across levels of consciousness, we present representative spontaneous electrophysiological recordings from mice and humans. In mice, local field potential (LFP) traces exhibit a clear progression as anesthesia deepens, with activity increasingly dominated by large, stereotyped Up–Down state transitions. As isoflurane concentration increases, Up states become shorter and occur less frequently, consistent with strengthened cortical bistability and a greater predominance of Down states (Figure S1A).

Human electroencephalography (EEG) recordings reveal a comparable state dependence. During anesthesia, the signal is dominated by large-amplitude slow oscillations, indicative of widespread cortical synchrony and reduced responsiveness. In contrast, wakefulness is characterized by lower-amplitude, desynchronized activity with faster components, consistent with active and differentiated neural processing (Figure S1B). Together, these examples provide qualitative insight into how spontaneous brain activity is reorganized across species as the brain transitions between conscious and anesthetized states.

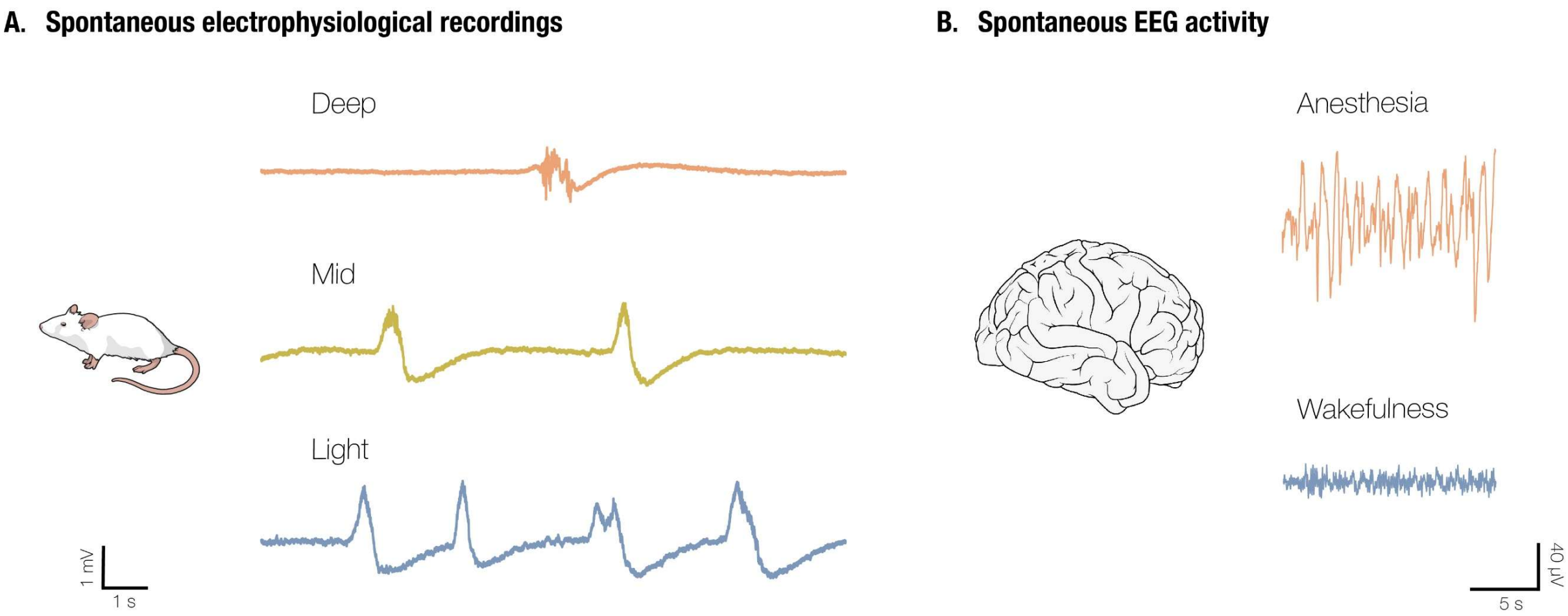

Figure S1. **Illustrative spontaneous electrophysiological recordings. (A)** Spontaneous LFP traces from the same mouse under three different levels of graded anesthesia, defined by increasing isoflurane concentrations. As observed, neural activity under anesthesia becomes dominated by stereotyped Up–Down state transitions characteristic of the anesthetized cortex. **(B)** Representative human EEG patterns during anesthesia, characterized by dominant slow oscillations, and during wakefulness, marked by lower-amplitude, desynchronized activity with prominent alpha rhythms.

Supplementary Note 2. FDT violations correlate with the frequency of the slow oscillation in animal models

Slow oscillations (SO) are a hallmark of brain states associated with reduced complexity. They are prominently observed during non-rapid eye movement sleep, in patients with unresponsive wakefulness syndrome (UWS), even during eyes-open periods, and in pathological conditions such as stroke [S1, S2, S3, S4]. These rhythms reflect cortical bistability, arising from alternating phases of neuronal activity and silence [S5]. Experimental and computational studies across humans, rodents, and cortical slice preparations converge in identifying cortical bistability and OFF-periods as major disruptors of large-scale network integration and complexity [S6, S7, S8, S9, S10]. In this framework, such bistable dynamics are thought to constrain the emergence of rich, differentiated cortical responses, thereby reducing perturbational complexity and FDT violations.

In the mouse anesthesia dataset, the dominant frequency of SO varied systematically across anesthesia levels and showed a strong monotonic relationship with anesthesia depth (Spearman's ρ = 0.87, $p < 0.001$), indicating that slower oscillatory dynamics were associated with deeper anesthesia. PCI was significantly correlated with SO frequency (Spearman's ρ = 0.69, $p < 0.001$; Pearson's r = 0.76, $p < 0.001$; Figure S2A), consistent with the idea that slower and more bistable cortical dynamics are associated with reduced perturbational complexity. Likewise, FDT violations exhibited an even stronger association with SO frequency (Spearman's ρ = 0.80, $p < 0.001$; Pearson's r = 0.83, $p < 0.001$; Figure S2B), indicating that the dominant frequency of spontaneous slow oscillations is closely linked to the degree of effective departure from equilibrium estimated from the whole-brain model.

We further evaluated robustness using leave-one-subject-out cross-validation. In held-out subjects, the association remained significant for PCI (Spearman's ρ = 0.6252, p = 0.0011; Pearson's r = 0.6567, $p < 0.001$) and for FDT violations (Spearman's ρ = 0.8139, $p < 0.001$; Pearson's r = 0.8169, $p < 0.001$).

Together, these findings show that the dominant frequency of SO not only tracks graded anesthesia depth but is also tightly linked to both perturbational complexity and non-equilibrium brain dynamics. This result is consistent with the idea that slow oscillatory bistable regimes impose strong constraints on large-scale integration, thereby reducing both the complexity of evoked cortical responses and the degree to which spontaneous activity departs from equilibrium.

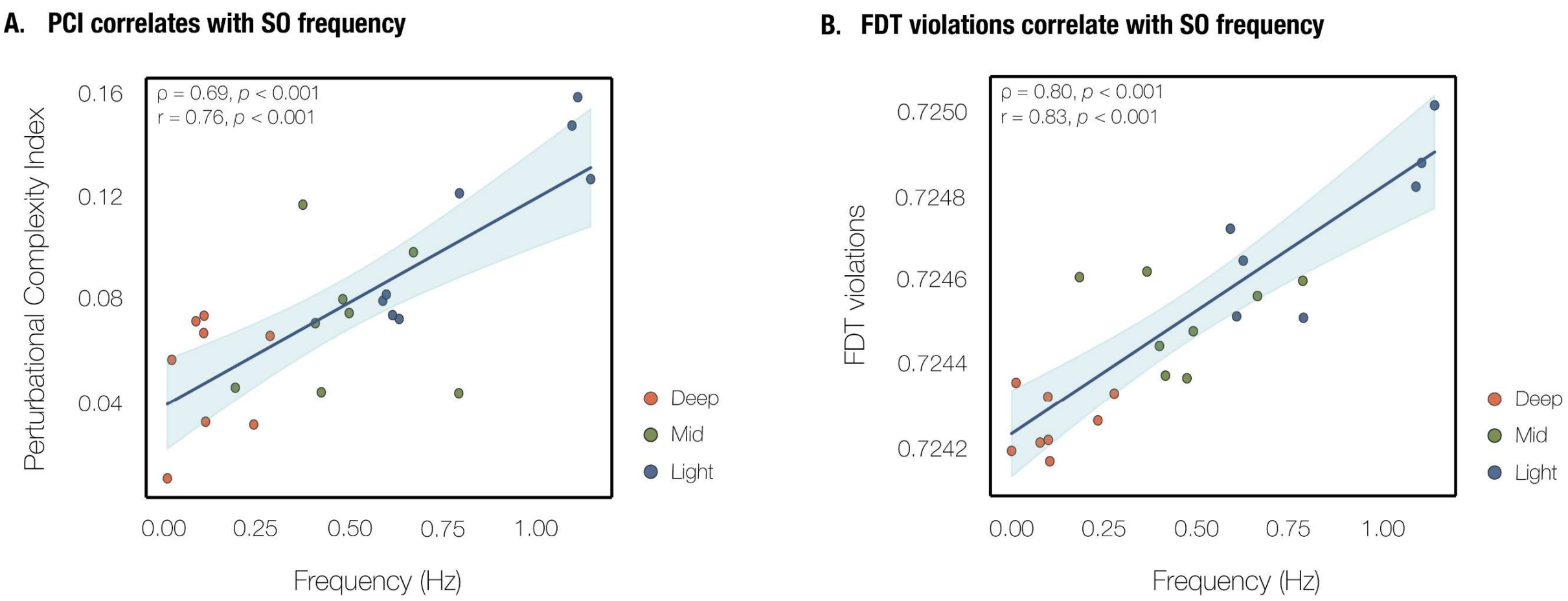

Figure S2. **Intrinsic slow oscillation (SO) frequency correlates with PCI and FDT violations in animal models. (A)** PCI correlates with the intrinsic frequency of the SO (Spearman's ρ = 0.69, $p < 0.001$; Pearson's r = 0.76, $p < 0.001$). It generalizes under leave-one-subject-out cross-validation (Spearman's ρ = 0.6252, $p = 0.0011$; Pearson's r = 0.6567, $p < 0.001$). **(B)** FDT violations also show a strong correlation with the intrinsic frequency of the SO, capturing its spatiotemporal structure with high sensitivity (Spearman's ρ = 0.80, $p < 0.001$; Pearson's r = 0.83, $p < 0.001$). It generalizes under leave-one-subject-out cross-validation (Spearman's ρ = 0.8139, $p < 0.001$; Pearson's r = 0.8169, $p < 0.001$).

Supplementary Note 3. Post-anoxic patients define a boundary condition of the framework

To better define the scope of the main findings, we analyzed the subgroup of patients with post-anoxic etiology separately from the primary non-anoxic disorders of consciousness (DoC) cohort. This subgroup was not included in the main analysis because severe post-anoxic injury is associated with diffuse cortical damage, marked suppression of electrophysiological activity, and low-voltage EEG, conditions under which both perturbational and spontaneous measures may become constrained by cortical integrity rather than providing a meaningful graded index of consciousness capacity [S11, S12].

The post-anoxic subgroup included 9 patients. All patients underwent the same acquisition, preprocessing, PCI computation, and FDT violations estimation procedures as described for the main DoC cohort. In this subgroup, PCI showed a marked floor effect. In particular, all UWS patients had PCI values at floor, consistent with absent or severely reduced perturbational responses. Accordingly, PCI still separated diagnostic categories at the group level (Cliff's $\delta = 1.0$, $p = 0.0108$; AROC = 1.0, Figure S3A). Under these conditions, however, PCI does not provide a meaningful graded variable, and therefore correlations cannot be interpreted in the same way as in the main non-anoxic DoC cohort.

Spontaneous EEG activity in the post-anoxic subgroup was frequently low-voltage, reducing the effective signal-to-noise ratio available for model-based estimation of directional and non-equilibrium structure. Nevertheless, FDT violations could still be estimated and retained variability across patients. At the group level, separation of FDT violations between diagnostic categories was modest and did not reach statistical significance (Cliff's $\delta = 0.700$, $p = 0.11$; Figure S3B). Given the modest separation, the limited sample size, and the likely heterogeneity in cortical injury and residual electrophysiological integrity within this subgroup, these results should be reported descriptively and interpreted cautiously (AROC = 0.85).

Because PCI was floor-compressed in UWS, we do not interpret this subgroup as providing an equivalent graded test of the relationship between PCI and FDT violations. Rather, these findings identify severe post-anoxic injury as a boundary condition of the framework, in which diffuse cortical damage constrains the dynamic range of both perturbational and spontaneous measures.

Taken together, these findings suggest that severe post-anoxic injury defines a clinically important boundary condition for the present framework. In this regime, diffuse cortical damage appears to compress the available electrophysiological repertoire and reduce the discriminatory range of both perturbational and spontaneous measures. Accordingly, the main conclusions of the study should be interpreted as applying to conditions in which spontaneous and perturbational brain dynamics remain sufficiently preserved to support meaningful estimation of PCI and FDT violations.

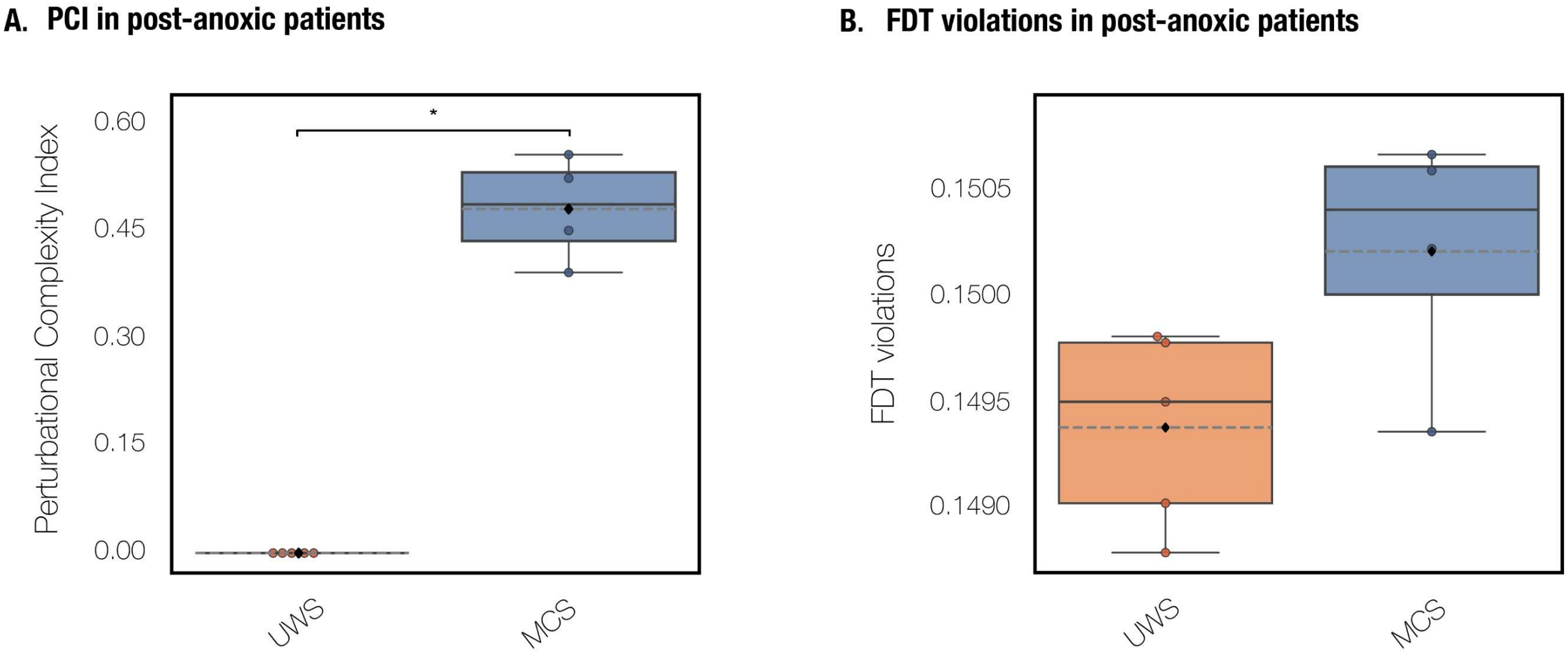

Figure S3. **PCI and FDT violations in the post-anoxic subgroup. (A)** PCI values in post-anoxic patients separated UWS and MCS at the group level (Cliff's $\delta = 1.0$, p = 0.0108; AROC = 1.0), but this separation was driven by a marked floor effect in UWS, with all UWS patients showing PCI values at floor. **(B)** FDT violations retained variability across post-anoxic patients, but separation between diagnostic categories was modest and did not reach statistical significance (Cliff's $\delta = 0.700$, p = 0.11; AROC = 0.85). These results identify severe post-anoxic injury as a boundary condition of the framework, in which diffuse cortical damage constrains the dynamic range of perturbational and spontaneous measures. * $p < 0.05$

Supplementary Note 4. FDT violations correlate with other non-equilibrium measures

Entropy production is one of the canonical physical measures of deviation from equilibrium. In the model used in this work, the entropy production rate (EPR) can be calculated analytically [S13]. We consider a linear Langevin system described by:

$$\frac{dx}{dt} = -Bx(t) + \eta(t) \qquad (S1)$$

where $\eta(t)$ is homogeneous white Gaussian noise, satisfying $< \eta(t)\eta(t') > = 2\,\sigma^2\delta(t-t')$. The EPR is then given by:

$$\phi = -\frac{1}{\sigma^2} tr(B\Theta) \qquad (S2)$$

Here, $\Theta = BK - \sigma^2 I$, where K is the stationary covariance matrix. We then computed the EPR for each fitted brain model according to Equation S2. A key characteristic of non-equilibrium systems is their temporal irreversibility. Following previous work [S14, S15], we quantified this irreversibility by measuring the asymmetry of time-lagged covariances. Specifically, we computed the difference between the lagged covariance matrix and its transpose:

$$A_c = \left\| KS^{model}(\tau) - (KS^{model}(\tau))^T \right\|_{\tau=dt} \qquad (S3)$$

where $\|\cdot\|$ denotes the Frobenius norm, i.e., the square root of the sum of the squared matrix elements, and A_C quantifies the asymmetry of the lagged covariances evaluated at $\tau = dt$.

Lastly, we examined the asymmetry of the fitted coupling matrix, which has been highlighted as a key mechanism for breaking detailed balance in interacting systems [S15], inducing FDT violations [S16]. Following previous work [S17], we characterized coupling asymmetry using irreciprocity. We define the reciprocated portion of the coupling between two nodes:

$$\overleftrightarrow{B}ij = min(Bij, Bji) = \overleftrightarrow{B}ji \qquad (S4)$$

The global reciprocity of the coupling is then defined as the ratio between the total reciprocated weight and the total connection weight:

$$r(B) = \frac{\sum_{i\neq j} \overleftrightarrow{B}ij}{\sum_{i\neq j} Bij} \qquad (S5)$$

Finally, we define the irreciprocity of the coupling as $A_B = 1 - r$, which lies in the range [0,1]. A value of $A_B = 0$ corresponds to a perfectly reciprocated coupling, while $A_B = 1$ corresponds to a completely unreciprocated coupling.

We reanalyzed the three datasets incorporating the non-equilibrium measures described above.

Figure S4 shows the results for the mouse dataset. EPR (Figure S4A), temporal irreversibility (Figure S4B), and coupling asymmetry (Figure S4C) all decreased with increasing anesthesia depth. These metrics showed clear separation between light and deep anesthesia, whereas the intermediate condition was less consistently distinguished from the other two. Nevertheless, the Spearman correlation coefficients indicate that all three metrics tracked anesthesia depth across conditions (EPR: Spearman's $\rho = 0.67$, $p < 0.001$; temporal irreversibility: Spearman's $\rho = 0.67$, $p < 0.001$; coupling asymmetry: Spearman's $\rho = 0.70$, $p < 0.001$). Furthermore, FDT violations were positively associated with EPR (Spearman's $\rho = 0.72$, $p < 0.001$; Pearson's $r = 0.66$, $p < 0.001$), temporal irreversibility (Spearman's $\rho = 0.72$, $p < 0.001$; Pearson's $r = 0.78$, $p < 0.001$), and coupling asymmetry (Spearman's $\rho = 0.77$, $p < 0.001$; Pearson's $r = 0.70$, $p < 0.001$).

Figure S5 presents the results for the three metrics in the human EEG recordings obtained during wakefulness and anesthesia. Across participants, EPR (Figure S5A), temporal irreversibility (Figure S5B), and coupling asymmetry (Figure S5C) were reduced under xenon and propofol relative to wakefulness. No significant differences were observed between ketamine and wakefulness, consistent with the main results for PCI and FDT violations. As observed for FDT violations, EPR, temporal irreversibility, and coupling asymmetry were higher in participants who reported conscious experience (wakefulness and ketamine; B+) than in those who did not (xenon and propofol; B-). FDT violations were positively associated with EPR (Spearman's $\rho = 0.67$, $p < 0.001$; Pearson's $r = 0.60$, $p < 0.001$), temporal irreversibility (Spearman's $\rho = 0.67$, $p < 0.001$; Pearson's $r = 0.66$, $p < 0.001$), and coupling asymmetry (Spearman's $\rho = 0.64$, $p < 0.001$; Pearson's $r = 0.63$, $p < 0.001$) (Figures S5D–F).

Lastly, we analyzed the DoC dataset (Figure S6). All non-equilibrium measures showed lower values in patients without signs of consciousness (UWS) than in patients with conscious signs (MCS−/MCS+), mirroring the pattern observed for FDT violations and PCI. These results should be interpreted cautiously, as the modest sample size and clinical heterogeneity of the DoC cohort may have limited statistical power despite directional consistency with the main findings. At the pooled level, FDT violations showed a significant positive correlation with temporal irreversibility (Spearman's $\rho = 0.51$, $p = 0.038$; Pearson's $r = 0.50$, $p = 0.0412$). EPR and coupling asymmetry showed positive trend-level associations in the same direction (EPR: Spearman's $\rho = 0.48$, $p = 0.051$; Pearson's $r = 0.44$, $p = 0.0762$; coupling asymmetry: Spearman's $\rho = 0.47$, $p = 0.0583$; Pearson's $r = 0.46$, $p = 0.0643$).

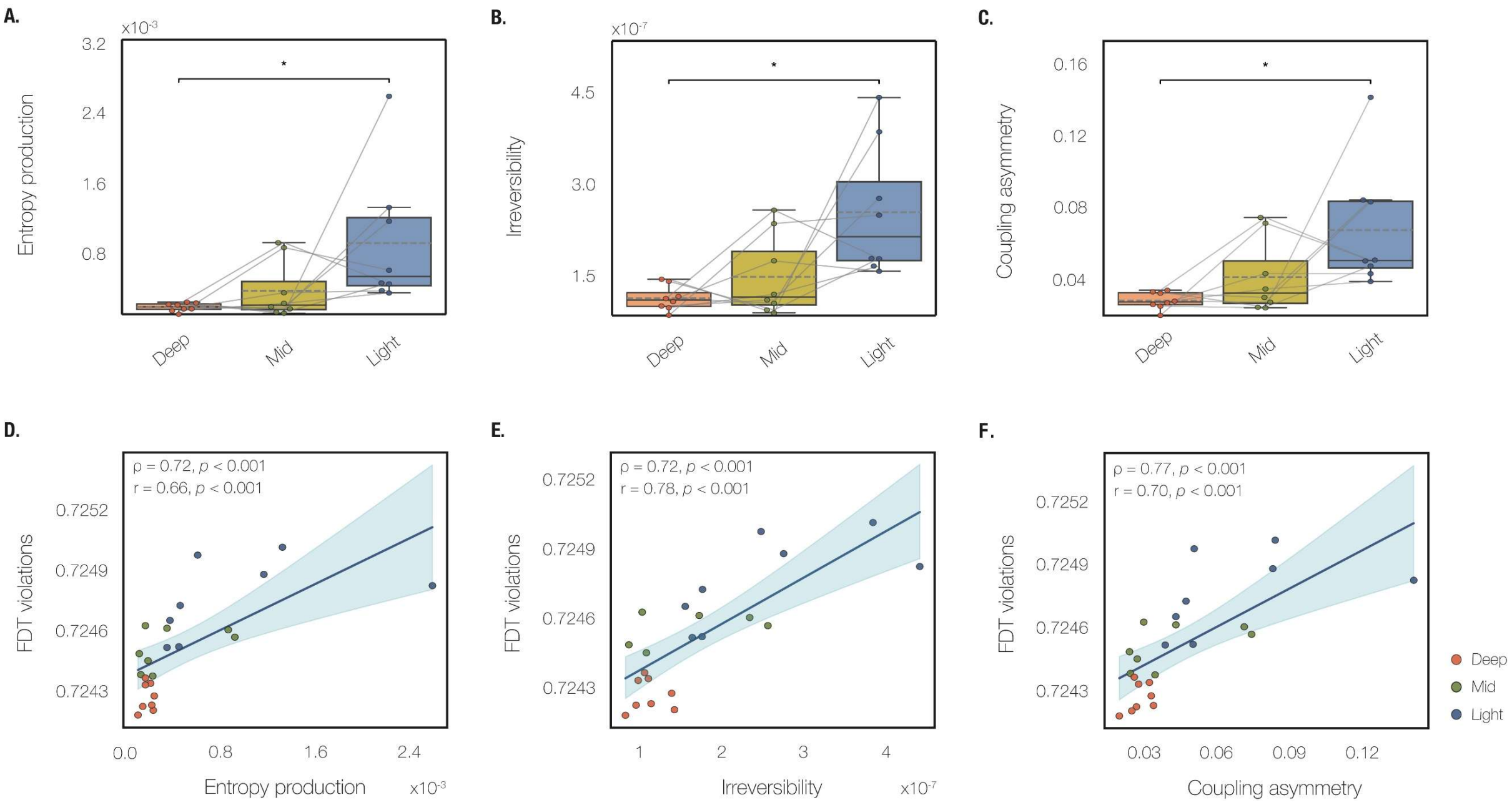

Figure S4. **FDT violations and complementary non-equilibrium measures track levels of anesthesia in mice. (A)** Entropy production rate (EPR), estimated from the whole-brain model fitted to spontaneous activity across graded anesthesia levels. **(B)** Temporal irreversibility computed from the asymmetry of time-lagged covariances in the fitted linear stochastic model. **(C)** Coupling asymmetry, quantified by irreciprocity, derived from the fitted effective coupling matrix. **(D)** FDT violations were positively associated with EPR (Spearman's ρ = 0.72, $p < 0.001$; Pearson's r = 0.66, $p < 0.001$). **(E)** FDT violations were positively associated with temporal irreversibility (Spearman's ρ = 0.72, $p < 0.001$; Pearson's r = 0.78, $p < 0.001$). **(F)** FDT violations were positively associated with coupling asymmetry (Spearman's ρ = 0.77, $p < 0.001$; Pearson's r = 0.70, $p < 0.001$). Across measures, deeper anesthesia was associated with reduced non-equilibrium dynamics. Pairwise comparisons were performed using Wilcoxon signed-rank tests with Holm-Bonferroni correction. * $p < 0.05$

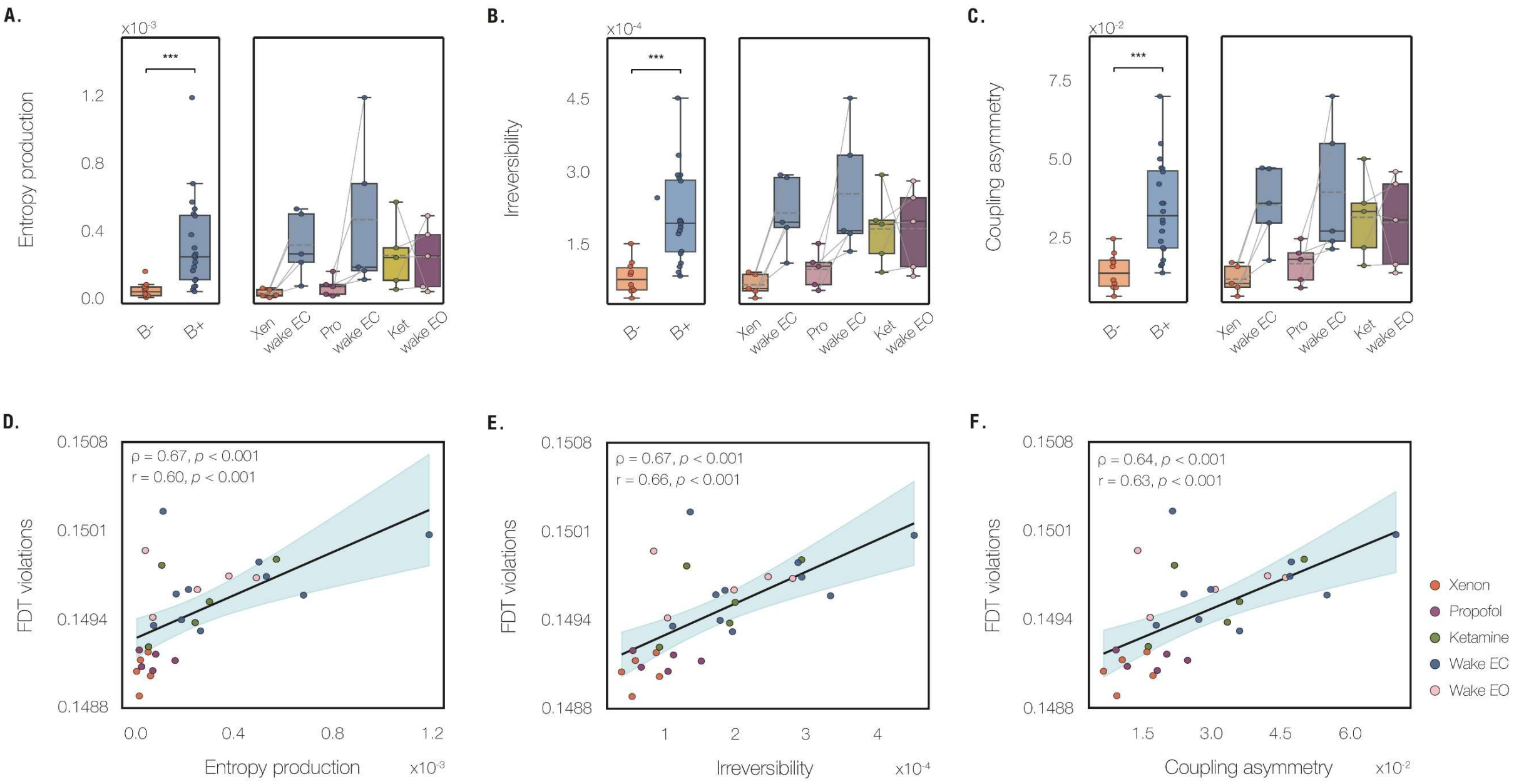

Figure S5. **FDT violations and non-equilibrium measures across wakefulness and anesthesia in humans. (A)** Entropy production rate across anesthetic conditions and grouped based on participants that reported conscious experience (wakefulness and ketamine; B+) and those who did not (xenon and propofol; B-). **(B)** Temporal irreversibility computed from the fitted linear stochastic model across conditions. **(C)** Coupling asymmetry, quantified by irreciprocity, across conditions. **(D)** FDT violations were positively associated with EPR (Spearman's ρ

= 0.67, $p < 0.001$; Pearson's r = 0.60, $p < 0.001$). **(E)** FDT violations were positively associated with temporal irreversibility (Spearman's ρ = 0.67, $p < 0.001$; Pearson's r = 0.66, $p < 0.001$). **(F)** FDT violations were positively associated with coupling asymmetry (Spearman's ρ = 0.64, $p < 0.001$; Pearson's r = 0.63, $p < 0.001$). Xenon and propofol showed reduced non-equilibrium dynamics relative to wakefulness, whereas ketamine remained closer to wakefulness, in line with the main manuscript results for PCI and FDT violations. *** $p < 0.001$

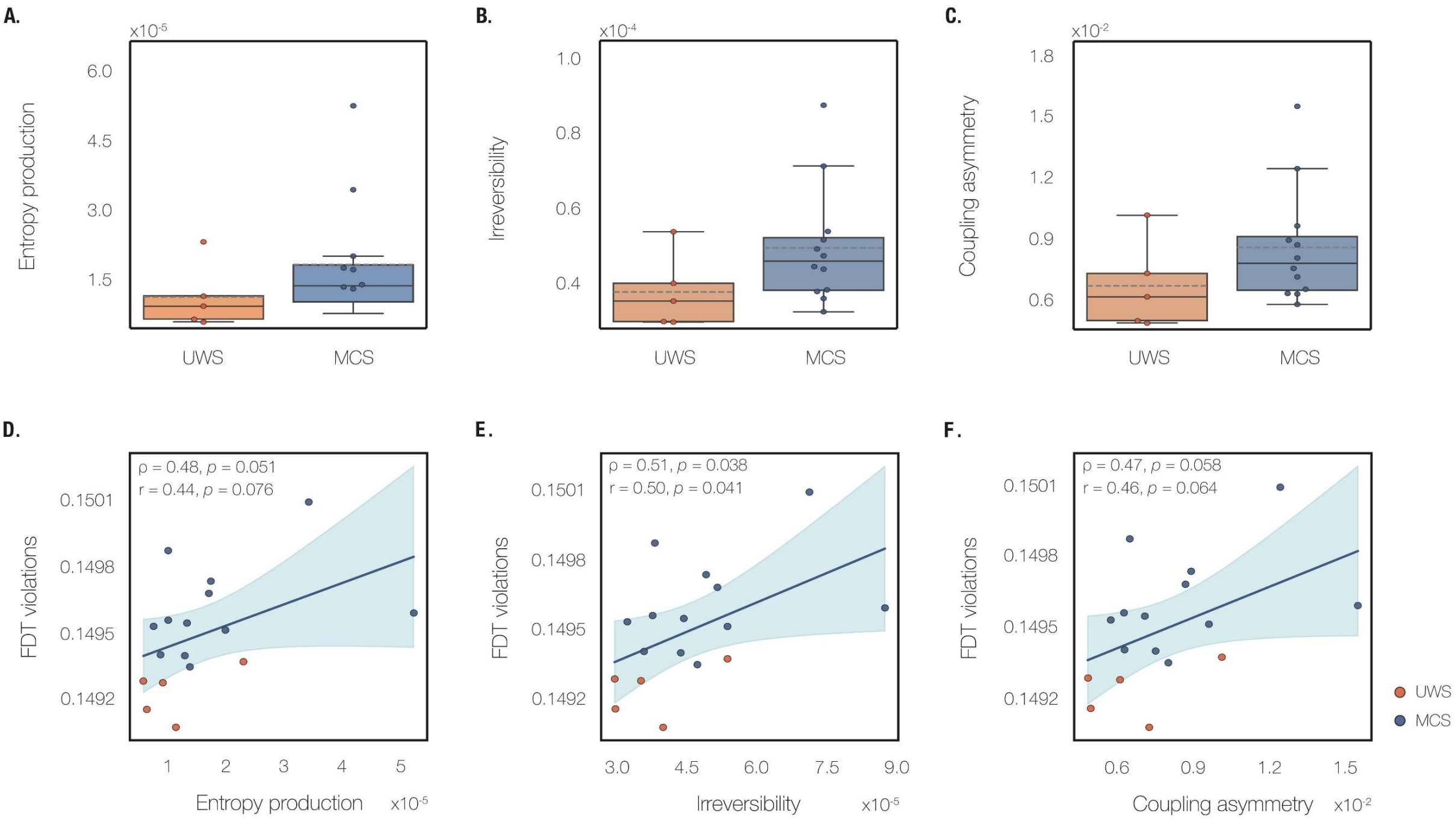

Figure S6. **FDT violations and non-equilibrium measures in disorders of consciousness. (A)** Entropy production rate (EPR) in patients with and without behavioral signs of consciousness. **(B)** Temporal irreversibility across diagnostic groups. **(C)** Coupling asymmetry, quantified by irreciprocity, across diagnostic groups. **(D)** Association between FDT violations and EPR (Spearman's ρ = 0.48, $p = 0.051$; Pearson's r = 0.44, $p = 0.0762$). **(E)** Association between FDT violations and temporal irreversibility (Spearman's ρ = 0.51, $p = 0.038$; Pearson's r = 0.50, $p = 0.0412$). **(F)** Association between FDT violations and coupling asymmetry (Spearman's ρ = 0.47, $p = 0.0583$; Pearson's r = 0.46, $p = 0.0643$). All three non-equilibrium measures showed lower values in UWS than in MCS−/MCS+, mirroring the pattern observed for PCI and FDT violations. However, only temporal irreversibility showed a significant positive association with FDT violations in the pooled DoC cohort, whereas EPR and coupling asymmetry showed positive trend-level associations.

Supplementary Note 5. Extension of human EEG experimental design and data curation

Electrode layouts and montages

Figure S7 illustrates the electrode layouts and digitized montages used for the human EEG recordings in two experimental setups. Figure S7A shows the digitized electrode positions for the Nexstim EEG system (Nexstim Plc.), which consists of 60 channels. Figure S7B displays the digitized layout for the BrainAmp EEG system (Brain Products GmbH), comprising 64 channels and used in a small subset of DoC recordings.

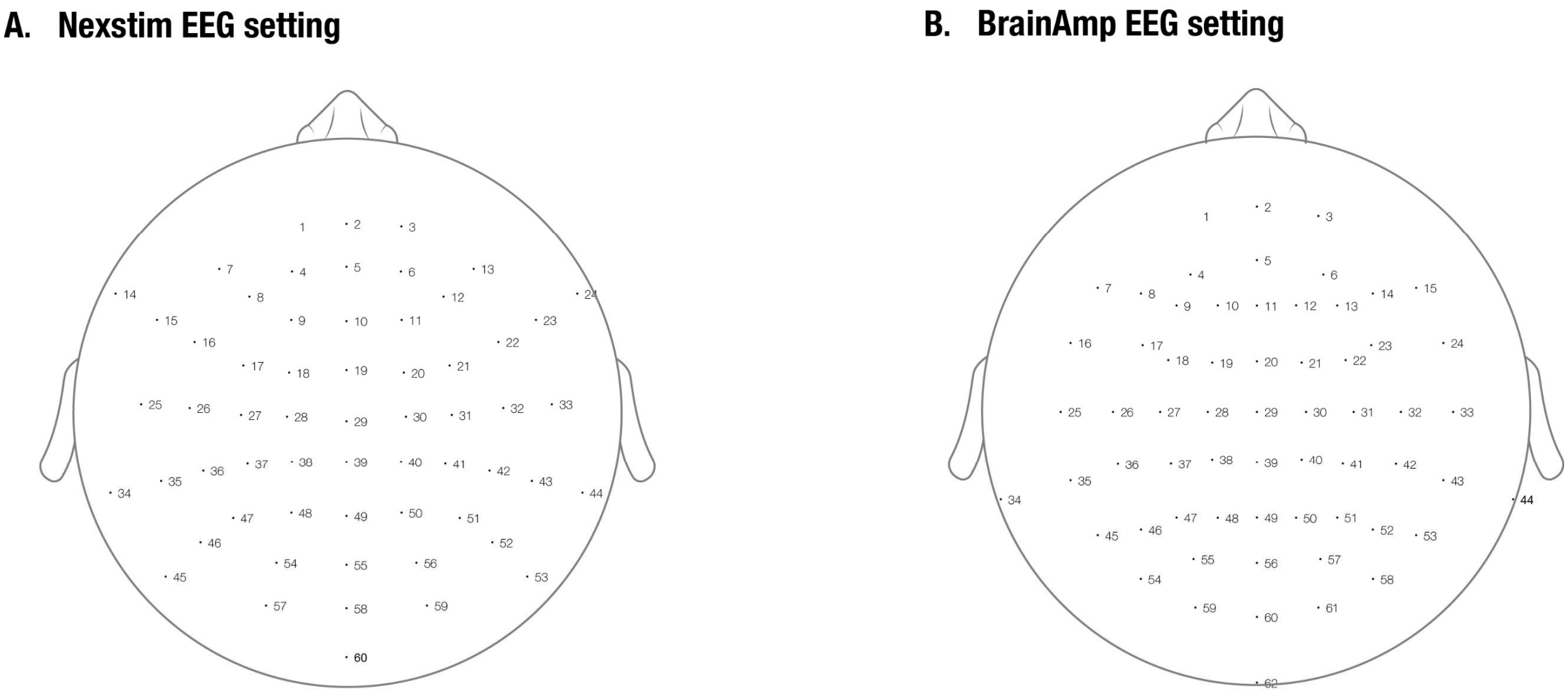

Figure S7. **Human EEG electrode layouts and montages. (A)** Digitization of the electrode positions for the Nexstim EEG system (Nexstim Plc., 60 channels). (**B)** Digitization of the electrode positions for the BrainAmp EEG system (Brain Products GmbH, 64 channels). Note the use of an external reference electrode on the forehead.

Drugs and exclusion criteria

Patients with DoC continued their standard pharmacological treatments during the EEG recording period. Specifically, antiepileptic medications were prescribed to 8 of the 17 patients. All patients in a chronic or persistent DoC state were free from sedative medications, while acute patients had been off sedatives for at least seven days prior to recording. Patients in a comatose state were excluded. Accordingly, no patient exhibited a flat or burst-suppression EEG pattern. Inclusion required at least one valid TMS-EEG session acquired either concurrently with, or in close temporal proximity to, a resting-state EEG recording.

Independent component analysis for the identification and removal of physiological artifacts

To ensure the stability of the Independent Component Analysis (ICA), we first reduced the dimensionality of the EEG data using eigenvalue decomposition, taking into account the number of rejected electrodes. Artifactual components were identified through visual inspection of each component's time series, power spectral density (PSD), and topographical distribution derived from the forward model. Ocular artifacts were typically characterized by pronounced temporal transients resembling eye movements or blinks, a PSD with elevated power at low frequencies and a steep spectral decay, and a prefrontal topography—often showing bilateral asymmetry for horizontal movements and symmetry for vertical movements and blinks. Muscular artifacts were identified by their fast, spiky temporal dynamics, occasionally organized into bursts, a broad high-frequency elevation in the PSD, and a topography indicative of localized superficial dipoles. Cardiac components were recognized by the periodic appearance of R-wave-like deflections in the time domain, a saw-tooth spectral shape associated with non-sinusoidal activity around 1 Hz, and, in some cases, a temporo-occipital distribution.

ICA-based cleaning was essential to reduce potential biases. Electrooculographic and sweat artifacts can increase low-frequency power, mimicking pathological slowing of brain activity, while electromyographic artifacts can artificially elevate power in the beta and gamma ranges, giving the false impression of increased fast cortical activity. Further details can be found in the original publications describing these datasets [S11, S12, S18, S19].

Post-anoxic subgroup: clinical and recording characteristics

The post-anoxic subgroup included 9 patients, of whom 5 were diagnosed with UWS and 4 with MCS, and underwent the same acquisition, preprocessing, PCI computation, and FDT estimation procedures as the primary non-anoxic DoC cohort. Short spontaneous EEG recordings were obtained either during the same TMS-EEG session (N = 8) or, when necessary, during a closely timed session (N = 1 within 2 weeks). Estimation of FDT violations was performed blind to behavioral diagnosis, PCI values, and other clinical information. EEG was recorded using Ag/AgCl electrodes and TMS-compatible amplifiers (Nexstim Plc., 60 channels, N = 9). When applicable, patients remained on their standard clinical treatments, including antiepileptic medication in 4 cases. This subgroup is reported separately because severe post-anoxic injury is frequently associated with diffuse cortical damage and low-voltage EEG, features that are important for interpreting both PCI and FDT violations.

Supplementary Note 6. FDT violations in the broadband range in animal models

To validate the focus on SO in the main analysis, which accounts for up to 95% of the spectral power, we repeated the analyses using broadband LFP signals within the same frequency range used for human EEG data. Overall, the results were qualitatively consistent with those obtained in the SO range, although the associations were somewhat noisier.

Broadband FDT violations also significantly distinguished all three levels of anesthesia (Figure S8A), as supported by a strong monotonic relationship with anesthesia depth (Spearman's $\rho = 0.870$, $p < 0.001$).

Broadband FDT violations were also significantly correlated with PCI (Spearman's $\rho = 0.69$, $p < 0.001$; Pearson's $r = 0.62$, $p = 0.00132$; Figure S8B). An ANCOVA including the condition interaction term revealed no evidence of slope heterogeneity between groups, indicating that the relationship was conserved across conditions ($F(2,18) = 0.114$, $p = 0.8933$; Freedman–Lane permutation $p_{perm} = 0.8945$). Leave-one-subject-out cross-validation further supported out-of-sample generalization (Spearman's $\rho = 0.6435$, $p < 0.001$; Pearson's $r = 0.541$, $p = 0.0063$). These results indicate that restricting the main analysis to SO does not create the observed relationship but rather enhances its sensitivity and interpretability in this dataset.

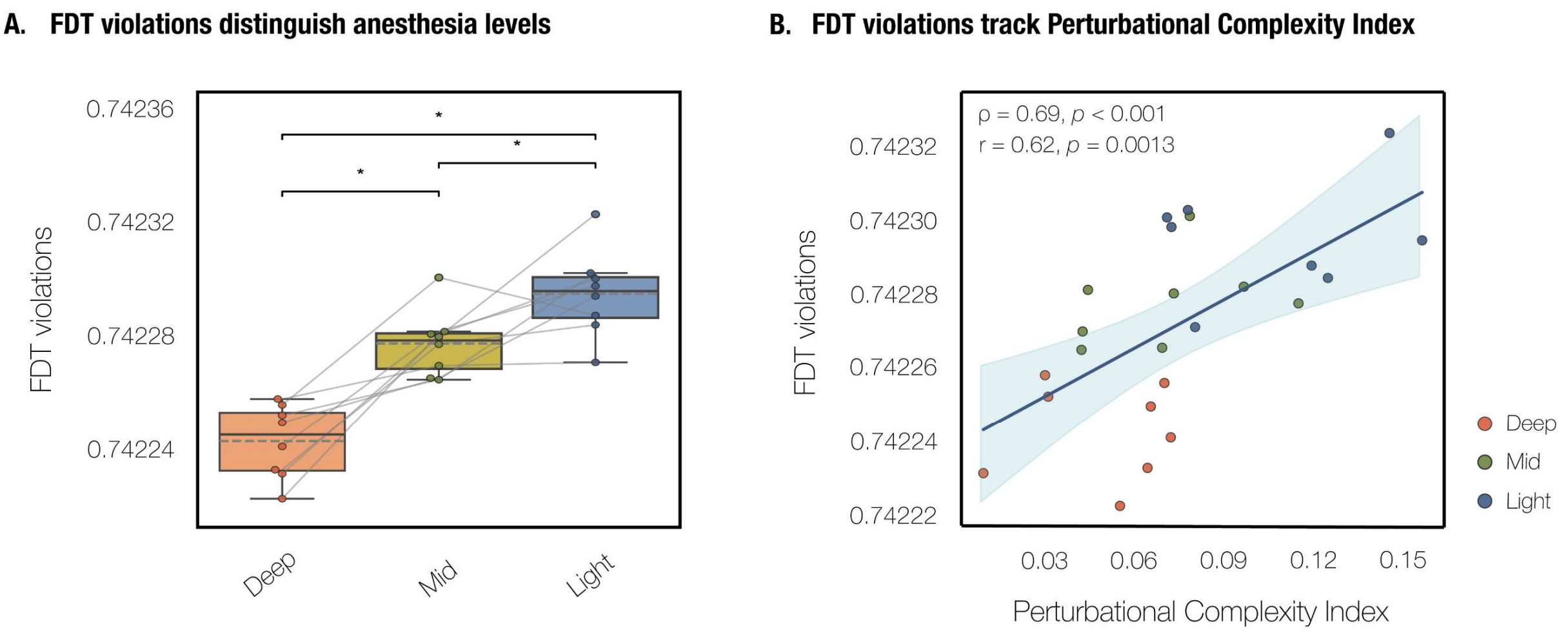

Figure S8. **FDT violations track levels of anesthesia in mice and correlate with the PCI in the broadband range. (A)** Broadband FDT violations, calculated from the whole-brain model fitted to empirical spontaneous activity, discriminated among all the conditions, as indicated by a strong monotonic relationship (Spearman's $\rho = 0.870$, $p < 0.001$). **(B)** Broadband FDT violations significantly correlated with PCI (Spearman's $\rho = 0.69$, $p < 0.001$; Pearson's $r = 0.62$, $p = 0.00132$). An ANCOVA including the condition interaction term revealed no evidence of slope heterogeneity between groups, indicating that the relationship was conserved across conditions ($F(2,18) = 0.114$, $p = 0.8933$; Freedman–Lane permutation $p_{perm} = 0.8945$). Leave-one-subject-out cross-validation supported out-of-sample generalization (Spearman's $\rho = 0.6435$, $p < 0.001$; Pearson's $r = 0.541$, $p = 0.0063$). All the pairwise comparisons were performed using Wilcoxon signed-rank tests and corrected post hoc with Holm-Bonferroni. * $p < 0.05$

Supplementary Note 7. Illustrative TMS-EEG traces for PCI computation

PCI values were obtained from previous studies [S11, S12, S20, S21]. Here, we show two representative TMS–EEG responses used for PCI computation (Figure S9). One example corresponds to a high PCI value, reflecting rich and differentiated evoked responses, whereas the other corresponds to a low PCI value, indicating a simpler and more stereotyped spatiotemporal response pattern.

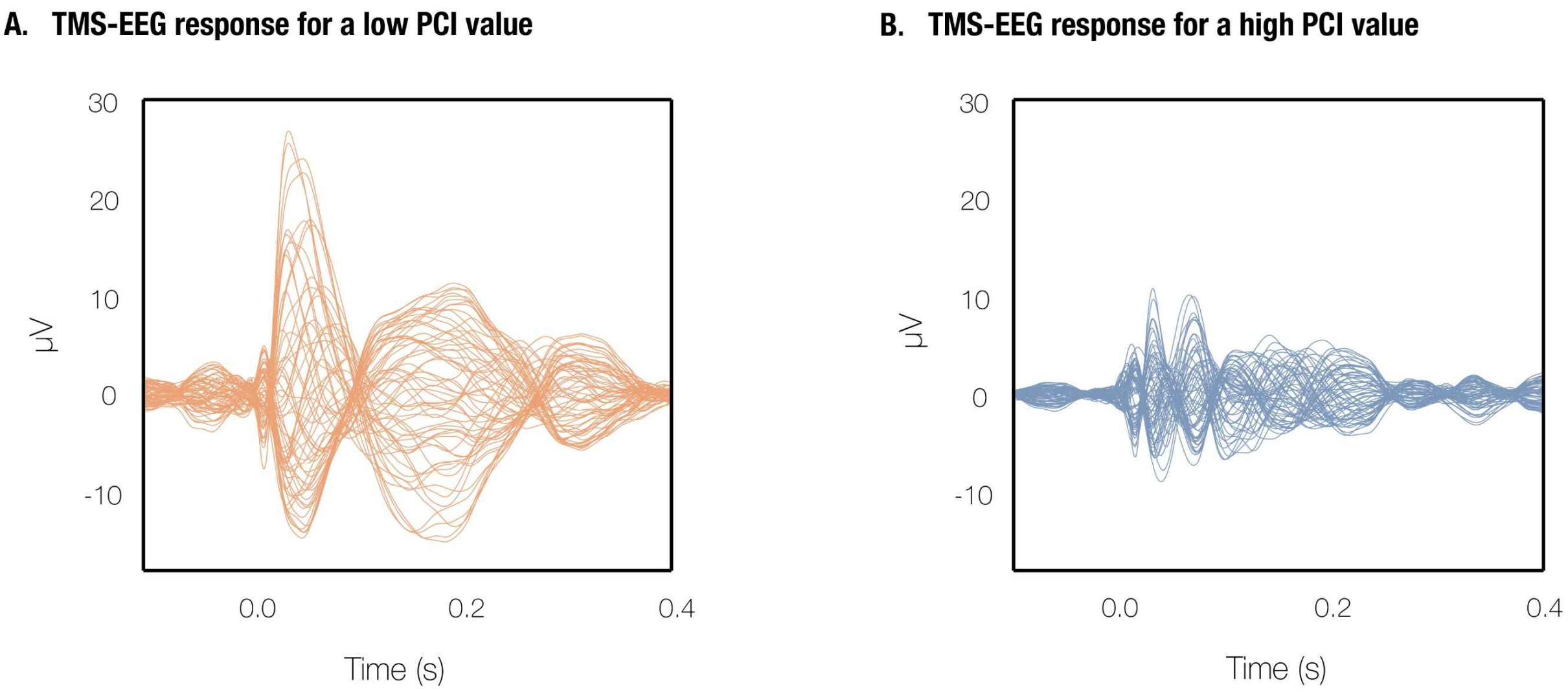

Figure S9. **TMS-EEG responses for PCI computation. (A)** Example of a TMS–EEG response with low complexity and a more stereotyped spatiotemporal pattern, corresponding to a low PCI value. **(B)** Example of a TMS–EEG response with rich and differentiated spatiotemporal dynamics, corresponding to a high PCI value.

Supplementary Note 8. Local weak stationarity control analyses

To evaluate the stationarity assumption underlying the multivariate Ornstein-Uhlenbeck framework, we performed additional epoch-based control analyses on signals subjected to the same core preprocessing used for the main FDT violation analysis. After preprocessing, recordings were segmented into 10-s windows with 50% overlap. Within each epoch, the mean and linear trend were removed. Local weak stationarity was then assessed using complementary Augmented Dickey-Fuller (ADF) and Kwiatkowski–Phillips–Schmidt–Shin (KPSS) tests, both evaluated at $\alpha = 0.05$. Epochs were classified as quasi-stationary when the ADF test rejected the null hypothesis of a unit root and the KPSS test failed to reject the null hypothesis of stationarity. These quantities were then summarized at the subject level, rather than treating overlapping epochs as independent observations.

Across the main datasets, the proportion of channel-epochs classified as quasi-stationary was high. In the mouse LFP dataset, the median (IQR) percentage of quasi-stationary channel-epochs was 100.0 (100.0–100.0) % in light anesthesia, 100.0 (99.9–100.0) % in mid anesthesia, and 99.4 (98.9–99.8) % in deep anesthesia. In the human anesthesia EEG dataset, the corresponding values were 100.0 (100.0–100.0) % in wakefulness with eyes closed, 100.0 (100.0–100.0) % in wakefulness with eyes open, 98.3 (90.8–100.0) % in xenon, 98.4 (97.9–99.5) % in propofol, and 99.8 (99.7–100.0) % in ketamine. In the non-anoxic DoC dataset, the values were 99.9 (99.7–100.0) % in MCS and 99.3 (97.6–99.7) % in UWS.

In the post-anoxic subgroup, the percentage of quasi-stationary channel-epochs was likewise high, reaching 99.8 (99.5–100.0) % in MCS and 99.7 (98.9–99.9) % in UWS. These values are reported for completeness, but this subgroup should be interpreted cautiously as a boundary condition given the low-voltage EEG patterns, limited sample size, and physiologically constrained nature of the recordings.

Overall, these results indicate that, after detrending and epoching, the large majority of analyzed segments were compatible with a local weak stationary approximation across all datasets. At the same time, these tests do not imply that the recordings form a single perfectly stationary regime over their full duration, nor do they exclude transient metastability or hidden state transitions. Rather, they support the use of a local weak stationarity approximation for the effective linear model.

Supplementary Note 9. Homogeneous noise in a linear Langevin system

In the case of spatially homogeneous noise, the normalized stationary-state autocorrelation does not depend on the value of the noise amplitude σ [S13]. Therefore, this parameter does not need to be fitted, as it does not provide additional information under the present modeling assumptions [S22]. In what follows, we provide proof of this.

Consider that the stationary-state autocorrelation function is given by:

$$C(\tau) = \langle x(t+\tau)x^T(t)\rangle \qquad (S6)$$

Under stationary conditions, we have:

$$x(t) = \int_{-\infty}^{t} e^{-B(t-s)}\eta(s)ds \qquad (S7)$$

$$x(t+\tau) = \int_{-\infty}^{t+\tau} e^{-B(t+\tau-s)}\eta(s)ds \qquad (S8)$$

From $C(\tau)$, the normalized stationary-state autocorrelation function for each node or brain region i can be derived as:

$$\frac{C_{ii}(\tau)}{C_{ii}(0)} = \frac{[e^{-B\tau}K]_{ii}}{K_{ii}} \qquad (S9)$$

When the noise amplitude is identical across all brain regions or nodes i (i.e. $\sigma_i = \sigma$), and denoting the identity matrix by I, the noise covariance is:

$$Q = 2\sigma^2 I \qquad (S10)$$

At stationarity, the covariance matrix K satisfies the continuous-time Lyapunov equation:

$$BK + KB^T = Q \qquad (S11)$$

Since this equation is linear in the noise variance, its solution can be written as $K = \sigma^2 K_0$, where K_0 satisfies $BK_0 + K_0B^T = 2I$. Thus, the normalized autocorrelation function for component i becomes:

$$\frac{C_{ii}(\tau)}{C_{ii}(0)} = \frac{[e^{-B\tau}\sigma^2 K_0]_{ii}}{[\sigma^2 K_0]_{ii}} = \frac{[e^{-B\tau}K_0]_{ii}}{[K_0]_{ii}} \qquad (S12)$$

From Equation S12, we can clearly see that in the case of homogeneous noise, the normalized stationary-state autocorrelation does not depend on the noise amplitude. Therefore, there is no need to fit the noise amplitude in the model, as all relevant information is embedded in the generative effective connectivity matrix B.

SUPPLEMENTAL REFERENCES

[S1] Massimini, M., Ferrarelli, F., Huber, R., Esser, S.K., Singh, H., and Tononi, G. (2005). Breakdown of Cortical Effective Connectivity during Sleep. Science *309*, 2228–2232.

[S2] Forgacs, P.B., Conte, M.M., Fridman, E.A., Voss, H.U., Victor, J.D., and Schiff, N.D. (2014). Preservation of electroencephalographic organization in patients with impaired consciousness and imaging-based evidence of command-following. Ann. Neurol. *76*, 869–879. https://doi.org/10.1002/ana.24283.

[S3] Andrillon, T., Poulsen, A.T., Hansen, L.K., Léger, D., and Kouider, S. (2016). Neural Markers of Responsiveness to the Environment in Human Sleep. J. Neurosci. *36*, 6583–6596. https://doi.org/10.1523/JNEUROSCI.0902-16.2016.

[S4] Edlow, B.L., Claassen, J., Schiff, N.D., and Greer, D.M. (2021). Recovery from disorders of consciousness: mechanisms, prognosis and emerging therapies. Nat. Rev. Neurol. *17*, 135–156. https://doi.org/10.1038/s41582-020-00428-x.

[S5] Sanchez-Vives, M.V., Massimini, M., and Mattia, M. (2017). Shaping the Default Activity Pattern of the Cortical Network. Neuron *94*, 993–1001. https://doi.org/10.1016/j.neuron.2017.05.015.

[S6] D'Andola, M., Rebollo, B., Casali, A.G., Weinert, J.F., Pigorini, A., Villa, R., Massimini, M., and Sanchez-Vives, M.V. (2018). Bistability, Causality, and Complexity in Cortical Networks: An In Vitro Perturbational Study. Cereb. Cortex *28*, 2233–2242. https://doi.org/10.1093/cercor/bhx122.

[S7] Cavelli, M.L., Mao, R., Findlay, G., Driessen, K., Bugnon, T., Tononi, G., and Cirelli, C. (2023). Sleep/wake changes in perturbational complexity in rats and mice. iScience *26*, 106186. https://doi.org/10.1016/j.isci.2023.106186.

[S8] Pigorini, A., Sarasso, S., Proserpio, P., Szymanski, C., Arnulfo, G., Casarotto, S., Fecchio, M., Rosanova, M., Mariotti, M., Lo Russo, G., et al. (2015). Bistability breaks-off deterministic responses to intracortical stimulation during non-REM sleep. NeuroImage *112*, 105–113. https://doi.org/10.1016/j.neuroimage.2015.02.056.

[S9] Rosanova, M., Fecchio, M., Casarotto, S., Sarasso, S., Casali, A.G., Pigorini, A., Comanducci, A., Seregni, F., Devalle, G., Citerio, G., et al. (2018). Sleep-like cortical OFF-periods disrupt causality and complexity in the brain of unresponsive wakefulness syndrome patients. Nat. Commun. *9*, 4427. https://doi.org/10.1038/s41467-018-06871-1.

[S10] Massimini, M., Corbetta, M., Sanchez-Vives, M.V., Andrillon, T., Deco, G., Rosanova, M., and Sarasso, S. (2024). Sleep-like cortical dynamics during wakefulness and their network effects following brain injury. Nat. Commun. *15*, 7207. https://doi.org/10.1038/s41467-024-51586-1.

[S11] Colombo, M.A., Comanducci, A., Casarotto, S., Derchi, C.-C., Annen, J., Viganò, A., Mazza, A., Trimarchi, P.D., Boly, M., Fecchio, M., et al. (2023). Beyond alpha power: EEG spatial and spectral gradients robustly stratify disorders of consciousness. Cereb. Cortex *33*, 7193–7210. https://doi.org/10.1093/cercor/bhad031.

[S12] Casarotto, S., Comanducci, A., Rosanova, M., Sarasso, S., Fecchio, M., Napolitani, M., Pigorini, A., G. Casali, A., Trimarchi, P.D., Boly, M., et al. (2016). Stratification of unresponsive patients by an independently validated index of brain complexity. Ann. Neurol. *80*, 718–729. https://doi.org/10.1002/ana.24779.

[S13] Godrèche, C., and Luck, J.-M. (2019). Characterising the nonequilibrium stationary states of Ornstein-Uhlenbeck processes. J. Phys. Math. Theor. *52*, 035002. https://doi.org/10.1088/1751-8121/aaf190.

[S14] Deco, G., Sanz Perl, Y., Bocaccio, H., Tagliazucchi, E., and Kringelbach, M.L. (2022). The INSIDEOUT framework provides precise signatures of the balance of intrinsic and extrinsic dynamics in brain states. Commun. Biol. *5*. https://doi.org/10.1038/s42003-022-03505-7.

[S15] Ohga, N., Ito, S., and Kolchinsky, A. (2023). Thermodynamic Bound on the Asymmetry of Cross-Correlations. Phys. Rev. Lett. *131*, 077101. https://doi.org/10.1103/PhysRevLett.131.077101.

[S16] Deco, G., Lynn, C.W., Sanz Perl, Y., and Kringelbach, M.L. (2023). Violations of the fluctuation-dissipation theorem reveal distinct nonequilibrium dynamics of brain states. Phys. Rev. E *108*, 064410. https://doi.org/10.1103/PhysRevE.108.064410.

[S17] Nartallo-Kaluarachchi, R., Asllani, M., Deco, G., Kringelbach, M.L., Goriely, A., and Lambiotte, R. (2024). Broken detailed balance and entropy production in directed networks. Phys. Rev. E *110*, 034313. https://doi.org/10.1103/PhysRevE.110.034313.

[S18] Colombo, M.A., Napolitani, M., Boly, M., Gosseries, O., Casarotto, S., Rosanova, M., Brichant, J.-F., Boveroux, P., Rex, S., Laureys, S., et al. (2019). The spectral exponent of the resting EEG indexes the presence of consciousness during unresponsiveness induced by propofol, xenon, and ketamine. NeuroImage *189*, 631–644. https://doi.org/10.1016/j.neuroimage.2019.01.024.

[S19] Massimini, M., and Laureys, S. (2017). Rest EEG recordings in healthy subjects during wakefulness, sleep and anesthesia with ketamine, propofol, and xenon. (Zenodo). https://doi.org/10.5281/ZENODO.806176.

[S20] Sarasso, S., Boly, M., Napolitani, M., Gosseries, O., Charland-Verville, V., Casarotto, S., Rosanova, M., Casali, A.G., Brichant, J.-F., Boveroux, P., et al. (2015). Consciousness and Complexity during Unresponsiveness Induced by Propofol, Xenon, and Ketamine. Curr. Biol. *25*, 3099–3105. https://doi.org/10.1016/j.cub.2015.10.014.

[S21] Dasilva, M., Camassa, A., Navarro-Guzman, A., Pazienti, A., Perez-Mendez, L., Zamora-López, G., Mattia, M., and Sanchez-Vives, M.V. (2021). Modulation of cortical slow oscillations and complexity across anesthesia levels. NeuroImage *224*, 117415. https://doi.org/10.1016/j.neuroimage.2020.117415.

[S22] Monti, J.M., Perl, Y.S., Tagliazucchi, E., Kringelbach, M.L., and Deco, G. (2025). Fluctuation-dissipation theorem and the discovery of distinctive off-equilibrium signatures of brain states. Phys. Rev. Res. *7*, 013301. https://doi.org/10.1103/PhysRevResearch.7.013301.
